# How Narcissism Shapes Responses to Antisocial and Prosocial Behavior: Hypo-Responsiveness or Hyper-Responsiveness?

**DOI:** 10.1177/01461672211007293

**Published:** 2021-04-15

**Authors:** Jiafang Chen, Barbara Nevicka, Astrid C. Homan, Gerben A. van Kleef

**Affiliations:** 1 University of Amsterdam, The Netherlands

**Keywords:** narcissism, social perception, responsiveness, moral character evaluation, reward/punishment

## Abstract

Narcissists have a relatively higher proclivity for displaying antisocial rather than prosocial behaviors, suggesting a comparatively higher tendency for unfavorably impacting societies. However, maintenance of social order also depends on appropriate responses to others’ social behavior. Once we focus on narcissists as observers rather than actors, their impact on social functioning becomes less clear-cut. Theoretical arguments suggest that narcissists could be either hypo-responsive or hyper-responsive to others’ social behavior. Across four studies, we examined narcissists’ responsiveness to variations in others’ antisocial and prosocial behaviors. Results showed that narcissists differentiated less between others’ antisociality/prosociality, as reflected in their subsequent moral character evaluations (Studies 1–4) and reward and punishment (Studies 3 and 4). These results suggest that narcissists are hypo-responsive to others’ social behaviors. Implications and directions for future research are discussed.

Narcissistic individuals, characterized by a grandiose sense of self, feelings of superiority, and a need for power (Carroll, 1987), tend to aspire to and indeed often attain powerful positions (Grijalva et al., 2015; Nevicka et al., 2011), which implies that social interactions across domains of life are often governed by narcissistic individuals. Given narcissists’ penchant for behaving antisocially (Bettencourt et al., 2006; Reidy et al., 2010), their predominance at higher levels of society seems disconcerting. However, societal functioning does not merely depend upon people’s own behavior but also on how they respond to others’ behavior. Over the course of history, societies have developed implicit norms that serve to consolidate principles of proper conduct (Fehr & Gächter, 2002), and discriminative responses to others’ antisocial and prosocial behaviors are essential for this purpose by discouraging antisocial behaviors and stimulating prosocial behaviors (Fehr & Fischbacher, 2004). Although it is well established that narcissists may negatively impact social order due to their high antisocial and low prosocial tendencies, it remains unclear how narcissism shapes responses to other people’s behavior. Here, we examined this question to illuminate how narcissism is related to responsiveness to others’ behavior, which can potentially affect social order.

Extant research on narcissism points to two alternative patterns of narcissists’ responsiveness. On one hand, narcissists’ motivation for maintaining a positive self-concept in the agentic domain (Grijalva & Zhang, 2016), which is associated with self-advancement (e.g., power, success; Trapnell & Paulhus, 2012), combined with a lack of interest in others (Morf & Rhodewalt, 2001) might make them less responsive to variations in other’s social behavior. On the other hand, given that deviance signals power and individuality (Bellezza et al., 2014; Van Kleef et al., 2011), others’ antisocial behavior might constitute a threat to narcissists’ power and uniqueness strivings (Morf & Rhodewalt, 2001), and thereby make them more strongly negatively responsive to others’ antisocial behavior. Thus, narcissists may be either apathetic to what happens around them or they may be active agents in admonishing antisocial others. In the present research, we examine these two competing hypotheses to provide insight into whether narcissists are hypo- or hyper-responsive to unknown others’ prosocial and antisocial behaviors.

## Theoretical Background

### Responses to Social Behaviors

Usually people respond distinctively to different kinds of behaviors, which in turn provides a clear code of conduct for others. For example, people may well respond punitively toward antisocial behaviors, such as with condemnation, blame, or punishment, signaling that these behaviors are unacceptable, and they respond with praise, credit, admiration, or respect to prosocial behaviors, signaling that these behaviors are appreciated (Brambilla et al., 2013; Hamilton et al., 1988). Through social learning, actors and observers can adjust their future behaviors based on this feedback, and consequently discourage future antisocial behaviors while reinforcing prosocial behaviors (Fehr & Fischbacher, 2004; Henrich et al., 2005).

In the current research, we focused on observers’ moral character evaluation of actors as the primary response to actors’ social behaviors, because this evaluation is most formative when individuals are forming overall impressions of others in social situations (vs. sociable and competent character evaluations; Brambilla & Leach, 2014; Goodwin et al., 2014). We also examined individuals’ behavioral reward and punishment responses that are more observable and therefore may more explicitly influence social order.

We examined the extent to which people recognize others’ behavior as being antisocial or prosocial as the underlying mechanism shaping observers’ responses. According to Jones’s (1991) and Schwartz’s (2016) ethical decision-making models, recognition, awareness, or interpretation of the situation and moral evaluation represent two distinct stages of the ethical decision-making process, with recognition preceding evaluation. This two-stage process also applies to person perception. The realistic accuracy model (Funder, 1995) posits that personality judgments hinge on the availability, detection, and utilization of behavioral cues indicative of that trait. Thus, the recognition of behavioral cues of a particular trait is distinct from, and an important prerequisite for, subsequent personality evaluation. Therefore, a sensitive observer should be able to first perceptually discriminate relevant behavioral cues in their surroundings and then interpret these cues when making character inferences about a person (Bernieri, 2001).

Usually, individuals displaying prosocial behaviors are evaluated as moral, and those committing antisocial behaviors as immoral (Batson et al., 2002; De Groot & Steg, 2009). Responsiveness refers to the degree to which individuals evaluate an actor behaving prosocially as moral, and an actor behaving antisocially as immoral. That is, the higher the evaluated moral (or immoral) character attributed to a target who behaves prosocially (or antisocially) relative to a target who behaves antisocially (or prosocially), the higher an observer’s responsiveness. Similarly, the higher the reward and the lower the punishment directed at prosocial versus antisocial others, the higher an observer’s responsiveness.

### Narcissism

Narcissism is defined as a pervasive pattern of grandiosity and self-importance (*Diagnostic and Statistical Manual of Mental Disorders* [4th ed.; *DSM-IV*]; American Psychiatric Association, 1994). We focused on grandiose agentic narcissism as a subclinical personality trait that distributes individuals on a continuum from low to high. Generally, narcissists (i.e., those scoring higher on narcissism) believe they are powerful, unique, and superior to others; show low interest in others (Morf & Rhodewalt, 2001); exhibit low empathy (Burgmer et al., 2019); display low interest in intimacy (Carroll, 1987); and are predisposed to exhibit antisocial behaviors such as aggression (Reidy et al., 2010).

The configuration of characteristics and motivations that typify narcissistic individuals could theoretically make them either more or less responsive to variations in other’s behavior. On one hand, narcissists’ motivation for maintaining a positive self-concept in the agentic domain and their low interest in others suggests a lower responsiveness to others’ behaviors—*hypo-responsiveness*. On the other hand, narcissists’ aggressive tendencies in response to ego threats point toward a possible overreaction to others’ antisocial behaviors—*hyper-responsiveness*.

#### Narcissism and hypo-responsiveness

Narcissistic people might show lower responsiveness to others’ social behaviors because of an information processing bias. According to the Iterative Reprocessing Model (van Bavel et al., 2012), motivations may lead to a rapid pre-appraisal of situational stimuli to be motivation-relevant or motivation-irrelevant and then sensitize individuals to motivation-relevant stimuli. For example, neuroscience research demonstrated that the amygdala processes motivation-relevant information more actively than motivation-irrelevant information (Cunningham et al., 2008). People high (vs. low) in narcissism show higher strivings for power, competence, and uniqueness (Gebauer et al., 2012), while they are simultaneously relatively less concerned about others (Burgmer et al., 2019) and have lower interest in intimacy (Carroll, 1987). Corresponding with these goals, narcissists are predominantly motivated to enhance their positive self-concept in the agentic domain, which emphasizes advancement in social hierarchies and involves pursuit of success and power, rather than in the communal domain, which emphasizes positive relationships, conformity, and benevolence (Grijalva & Zhang, 2016; Trapnell & Paulhus, 2012). Consequently, motivation-irrelevant information in the communal domain may be less likely to catch narcissists’ attention and thus be less recognizable because such information would be relatively less salient for them (Jones, 1991). Therefore, narcissists might be less likely to differentially respond to communal information, such as others’ antisocial and prosocial behaviors because the harmful/beneficial consequences of these behaviors for someone else are irrelevant to narcissists’ self-concern and personal motivation.

The situated focus theory of power (Guinote, 2007), which proposes that power promotes processing of goal-relevant information and inhibits processing of goal-irrelevant information, lends further support to the idea that narcissists may inhibit processing of morality-relevant information. Narcissists are likely to experience a heightened sense of power because they have a strong need for power (Carroll, 1987), are high in self- and other-reported dominance (Raskin et al., 1991), and are more likely to actually occupy powerful positions (Grijalva et al., 2015; Nevicka et al., 2011). Thus, narcissists may inhibit their processing of goal- or motivation-irrelevant information in the communal domain (e.g., others’ antisocial or prosocial behavior) and show lower responsiveness to such information.

There is some preliminary support for this proposition. For instance, high (vs. low) narcissistic individuals were less responsive to differences between others’ non-narcissistic and narcissistic profiles (Wallace et al., 2015). In addition, observers’ psychological communion (vs. agency that narcissists are attuned to) was positively related to their accuracy in rating targets’ personality characteristics (Vogt & Colvin, 2003). Moreover, narcissists were less willing to sanction integrity-norm violators (O’Reilly et al., 2018).

We build on this work to propose that narcissists may be hypo-responsive to others’ antisocial and prosocial behaviors, such that they show less differentiation in moral character evaluations and behavioral responses. Furthermore, following this reasoning, narcissists’ hypo-responsiveness would be mediated by their lower recognition of others’ behaviors as being antisocial or prosocial, given narcissists’ disproportionate attention to self-enhancing information in the agentic domain, and their comparatively low interest in others in the communal domain.

#### Narcissism and hyper-responsiveness

Alternatively, narcissists might show a more pronounced responsiveness to others’ antisocial behavior, because such behavior could be conceived as a threat to their power and uniqueness strivings. Previous research demonstrated that norm violators are seen as higher in power, status, and competence than people who obey social norms (Bellezza et al., 2014; Van Kleef et al., 2011), because of their apparent freedom to act at will (Stamkou et al., 2020; Van Kleef et al., 2011). Observers with a higher need for uniqueness are especially predisposed to infer more status and competence from others’ nonconforming versus conforming behaviors (Bellezza et al., 2014). Given narcissists’ fundamental need to attain power and status and to show off their superiority (Morf & Rhodewalt, 2001), others’ norm violations may be perceived as competing bids for power and status and thus could be construed as a threat and fuel narcissists’ aggression to protect their threatened self-concept—their normal modus operandi when reacting to ego threats (Bushman & Baumeister, 1998).

Consistent with this reasoning, several studies suggest that people higher (vs. lower) on narcissism-related characteristics, such as sense of power, dominance, socioeconomic status, and entitlement, respond more harshly to others’ antisocial behaviors (Stamkou et al., 2020). Based on these arguments, one might expect narcissists to be hyper-responsive to others’ antisocial behaviors. Moreover, given that narcissists are motivated to maintain their inflated self-concept, they should be more sensitive and accurate in recognizing antisocial behaviors that could threaten their powerful and unique status, resulting in hyper-responsiveness. Therefore, narcissists’ hyper-responsiveness might be mediated by their enhanced recognition of antisocial behavior.

While prosocial behavior can also constitute a route to power and status in certain situations (e.g., elevating social status by presenting generosity; Flynn et al., 2006), such prosocial behaviors are less likely to pose a threat to narcissists’ power and uniqueness strivings. This is because narcissists are generally disinterested in communal features (e.g., kindness) that underlie prosocial behaviors and instead are highly focused on enhancing their agentic self-concept (e.g., power; Gebauer et al., 2012), which is more closely linked with antisocial behaviors (Bargh et al., 1995; Lammers et al., 2010). This may explain why narcissism is positively related to antisocial behaviors (Reidy et al., 2010), generally negatively associated with prosocial behavior, and unrelated to prosocial self-enhancement (Nehrlich et al., 2019). Given that agency rather than communion is the preferred tool for narcissists’ self-presentation, narcissists would be unlikely to register others’ prosocial behaviors as a potential threat. Accordingly, narcissists’ hyper-responsiveness to others’ social behavior, if observed, may be limited to antisocial (vs. prosocial) behavior.

## Summary of Predictions and Overview of Studies

We examined the effect of observers’ narcissism on their responsiveness to others’ social behaviors across four studies. In Study 1, we contrasted antisocial and control behaviors to test whether narcissists display hypo- or hyper-responsiveness to antisocial behaviors. If the hypo-responsiveness hypothesis is true, we would expect high (vs. low) narcissists to show a smaller difference in moral character evaluations between the two conditions. Conversely, if the hyper-responsiveness hypothesis is true, we would expect a larger difference for high narcissists. In Study 2, we manipulated prosocial versus control behaviors to examine the effect of narcissism on observers’ responsiveness on moral character evaluations. In Study 3, we contrasted antisocial and prosocial tendencies, and added behavioral measures of reward and punishment. Study 4 was a pre-registered replication study of Study 3. We examined the mediating role of recognized antisociality/prosociality in all studies.

## Statistical Power

G*Power (Faul et al., 2009) analysis with a small-to-medium effect size, *f*^2^ = .085, a significance level of α = .05, and a power of .80, recommended sample sizes of 146 (Study 1) and 133 (Studies 2, 3, and 4). For all studies, the minimum sample sizes were exceeded to account for possible participant dropout and to maximize statistical power, particularly for Study 1 which included an additional predictor (i.e., self-relevance) and necessitated testing of a three-way interaction and thus required a larger sample (Dawson & Richter, 2006).

## Study 1

Study 1 provides a first test of our hypo- and hyper-responsiveness hypotheses. In addition, we examined the potential influence of the self-relevance of others’ behavior on narcissists’ responsiveness to others’ antisocial behavior. Highly self-relevant antisocial behaviors are relatively proximate and thus more salient and likely to catch observers’ attention (Jones, 1991) and can indirectly harm observers, who may therefore be more sensitive and respond negatively (Stein et al., 2016). Indeed, Back et al. (2013) illustrated that narcissists showed revenge-oriented reactions when they imagined to be or were harmed by close others. Therefore, high self-relevance might attenuate hypo-responsiveness, such that individuals high (vs. low) in narcissism may respond similarly or even more negatively to others’ highly self-relevant antisocial behavior. In other words, hypo-responsiveness may only manifest in the low self-relevance situation. Alternatively, regarding the hyper-responsiveness hypothesis, self-relevance may further amplify narcissists’ responsiveness due to the potential indirect harm from the antisocial behavior. Therefore, hyper-responsiveness may exist in the low self-relevant situation because of the perceived competing threat to narcissists’ power and status by the antisocial actor but be enhanced further in the highly self-relevant situation. Testing the moderating role of self-relevance allows us to further differentiate between the competing hypo- and hyper-hypotheses, as self-relevance would either attenuate the effect (as for hypo-hypothesis) or further amplify it (as for hyper-hypothesis).

### Method

#### Participants

In total, 549 participants (45.4% female; *M*_age_ = 36.45, *SD*_age_ = 11.19) from the United States were recruited via Amazon Mechanical Turk (MTurk) to complete the survey for US$3. Five participants were excluded for exceeding the maximum given time (1 hr) to complete the study.

#### Procedure

Participants were randomly assigned to the conditions of a 2 (antisocial behavior vs. control behavior) × 2 (high self-relevance vs. low self-relevance) full-factorial design. Participants completed two measures of trait narcissism followed by a buffer measure NEO-Five Factor Invertory (NEO-FFI; Costa & McCrae, 1992). Next, participants were presented with a scenario, which described an actor’s behavior (see below). To enhance psychological realism, participants were asked to immerse themselves in this scenario for 2 min and then evaluate the moral character of the actor. Finally, they completed the measure of recognized antisociality and manipulation checks.

#### Materials

##### Narcissism

We focused on the global construct of grandiose narcissism rather than its underlying dimensions, like admiration and rivalry (Back et al., 2013). As such, we employed the Narcissistic Personality Inventory (NPI; Miller et al., 2012; Raskin & Terry, 1988) as a global measure of participants’ grandiose narcissism given its high validity and wide use. Participants rated whether each of 40 items applied to them (e.g., “I have a natural talent for influencing people”; 1 = *true*, 0 = *false*; α = .94). We also included the Narcissistic Admiration and Rivalry Questionnaire (NARQ; Back et al., 2013) to exploratorily examine the potential differences between the NPI and the NARQ dimensions regarding responsiveness.^1^

##### Manipulation of actor’s behavior

**Figure 1.**
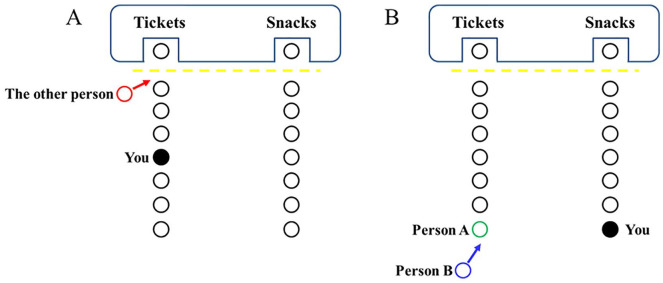
Diagrams of the manipulation (Studies 1 and 2): (A) actor’s behavior in the antisocial and high self-relevance conditions (Study 1), and (B) both the control and prosocial conditions (Study 2).

Actor’s behavior was manipulated by describing a scenario in which the actor pushed into a queue at the cinema (antisocial behavior) or lined up at the back of a queue (control behavior). Self-relevance was manipulated by indicating that the actor joined the tickets queue in which participants were standing (high self-relevance) or the adjacent snacks queue (low self-relevance; see Figure 1A for a visualization of the antisocial and high self-relevance condition, and Supplemental Materials for other conditions).

##### Moral character evaluation

Participants indicated their evaluations of the actor’s moral character by rating three adjectives (i.e., “honest,” “sincere,” “trustworthy”; α = .96; 1 = *not at all*, 7 = *very much so*; Leach et al., 2007).^2^

##### Recognized antisociality

Three statements were used to assess the degree to which participants recognized that the actor behaved antisocially (e.g., “I think this person behaved inappropriately”; α = .95; 1 = *strongly disagree*, 7 = *strongly agree*).

##### Manipulation checks

As a check of the antisocial behavior manipulation, participants indicated whether the actor “jumped in at the front of the queue” (1) or “lined up at the end of the queue” (0). As a check of the self-relevance manipulation, participants indicated whether the actor joined in the “tickets line” (1) or the “snacks line” (0).

### Results

Descriptive statistics and correlations are presented in Table 1.

**Table 1.** Means, Standard Deviations, and Correlations Between Variables (Studies 1 and 2).

Variable	*M*_S1_	*SD*_S1_	1	2	3	4	*M*_S2_	*SD*_S2_
1. Actor’s behavior	0.50	0.50	—	−.01	.76**	.48**	0.49	0.50
2. Narcissism	0.37	0.25	.05	—	−.08	.08	0.48	0.29
3. Recognized antisociality/prosociality	4.12	2.39	.89**	.10*	—	.61**	4.21	1.98
4. Moral character evaluation	3.33	1.77	−.72**	.01	−.77**	—	4.92	1.52
5. Self-relevance	0.49	0.50	−.004	.08	−.01	.04	—	—

*Note.* Study 1 (*N* = 444) correlations are presented below the diagonal and Study 2 (*N* = 249) correlations are presented above the diagonal. In Study 1, actor’s behavior and self-relevance were dummy coded (for actor’s behavior, control = 0, antisocial = 1; for self-relevance, low self-relevance = 0, high self-relevance = 1). In Study 2, actor’s behavior was dummy coded (control = 0, prosocial = 1).

* *p* < .05. **p < .01.

#### Manipulation checks

A chi-square test showed that participants in the antisocial condition (97.32%, *n* = 218 out of 224) were more likely to report that the actor jumped the queue than were those in the control condition (4.09%, *n* = 9 out of 220), χ^2^(1, *N* = 444) = 386.09, *p* < .001, ϕ = .93, 95% confidence interval [CI] = [0.90, 0.96]. Furthermore, participants in the high self-relevance condition (97.70%, *n* = 212 out of 217) were more likely to report that the actor joined in the same queue (tickets line) as themselves than were those in the low self-relevance condition (8.37%, *n* = 19 out of 227), χ^2^(1, *N* = 444) = 354.68, *p* < .001, ϕ = .89, 95% CI = [0.85, 0.93]. Thus, the manipulations were successful.^3^

#### Moral character evaluation

**Figure 2.**
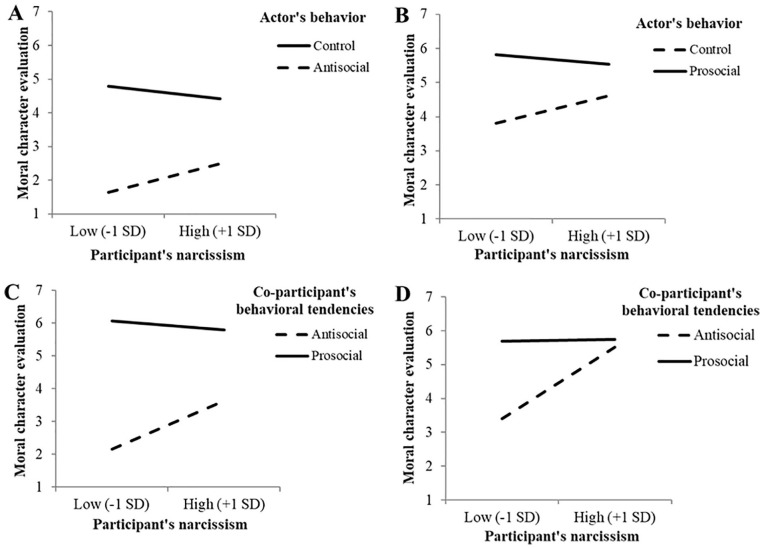
Responsiveness on moral character evaluation (Studies 1–4): Interaction between actor’s behavior and participant’s narcissism on moral character evaluation of the actor (A, Study 1; B, Study 2), and interaction between co-participant’s behavioral tendencies and participant’s narcissism on moral character evaluation of the co-participant (C, Study 3; D, Study 4); “high” and “low” narcissism refer to scores on the NPI scale that were 1 *SD* above the mean or 1 *SD* below the mean, respectively. *Note.* NPI = Narcissistic Personality Inventory.

We ran a regression analysis using Hayes’s (2013) Model 3 in PROCESS to examine the effects of narcissism on participants’ moral character evaluations (Table 2). The results yielded a significant main effect of actor’s behavior, with participants evaluating the actor in the antisocial condition (*M* = 2.07, *SD* = 1.36) as less moral than the actor in the control condition (*M* = 4.62, *SD* = 1.08). There was no significant main effect of narcissism or self-relevance. The three-way interactions and two-way interactions between actor’s behavior and self-relevance, and between self-relevance and narcissism were not significant. However, the anticipated two-way interaction between actor’s behavior and narcissism was significant (Figure 2A). Simple effect results revealed that both low (–1 *SD* on the NPI), *B* = −3.11, *t*(440) = −19.36, *p* < .001, *r* = .68, 95% CI = [–3.43, –2.80] and high (+1 *SD* on the NPI), *B* = −1.99, *t*(440) = −12.43, *p* < .001, *r* = .51, 95% CI = [–2.31, –1.68] narcissists rated the actor in the antisocial condition as less moral than the one in the control condition. However, the effect was significantly smaller for high narcissists.

**Table 2.** Regression Results on Moral Character Evaluation and Recognized Antisociality (Study 1).

Predictors	Moral character evaluation				Recognized antisociality			
	*B* [95% CI]	*t* (*df*)	*p*	*r*	*B* [95% CI]	*t* (*df*)	*p*	*r*
Actor’s behavior	−2.56 [–2.78, –2.33]	−22.36 (436)	<.001	.73	4.24 [4.05, 4.44]	43.02 (436)	<.001	.90
Narcissism	0.42 [–0.03, 0.87]	1.81 (436)	.071	.09	0.35 [–0.04, 0.74]	1.76 (436)	.079	.08
Self-relevance	0.06 [–0.16, 0.29]	0.56 (436)	.576	.03	0.01 [–0.19, 0.20]	0.07 (436)	.946	.003
Actor’s Behavior × Narcissism	2.19 [1.29, 3.10]	4.75 (436)	<.001	.22	−2.90 [–3.68, –2.11]	−7.28 (436)	<.001	.33
Actor’s Behavior × Self-Relevance	0.15 [–0.30, 0.60]	0.65 (436)	.513	.03	0.07 [–0.32, 0.46]	0.34 (436)	.734	.02
Narcissism × Self-Relevance	0.13 [–1.34, 2.29]	0.29 (436)	.772	.01	−0.01 [–0.79, 0.77]	−0.03 (436)	.977	.001
Actor’s Behavior × Narcissism × Self-Relevance	0.47 [–1.34, 2.29]	0.51 (436)	.609	.02	−0.91 [–2.47, 0.65]	−1.15 (436)	.252	.05

*Note.* 95% CIs are shown in brackets. The effect size is represented by Pearson’s *r*. For actor’s behavior, control = 0, antisocial = 1; for self-relevance, low self-relevance = 0, high self-relevance = 1. CI = confidence interval; *df* = degrees of freedom.

#### Mediated moderation model

**Figure 3.**
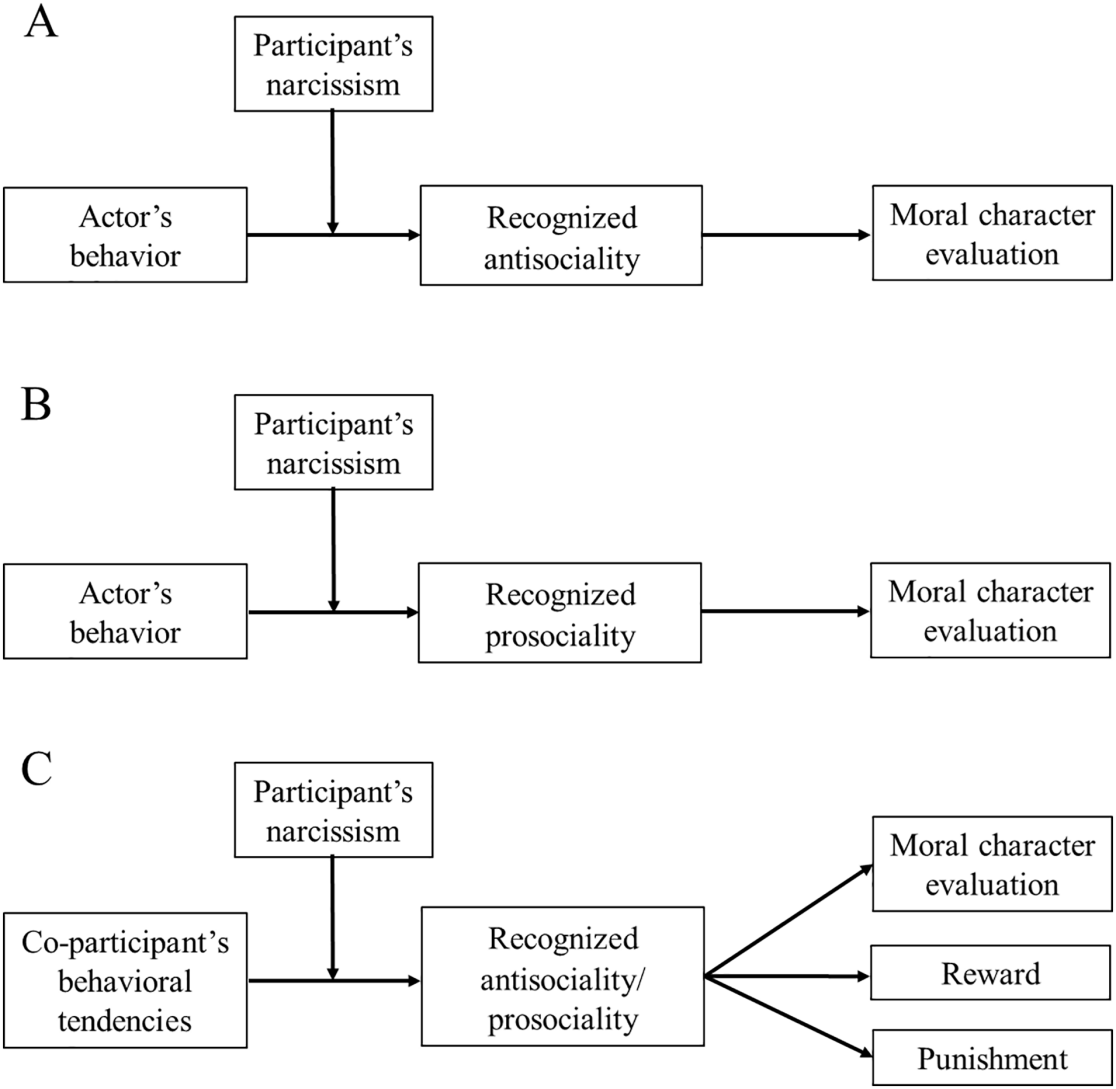
Mediated moderation models (Studies 1–4): Proposed mediated moderation models (A, Study 1; B, Study 2; C, Studies 3 and 4); actor’s behavior was either control or antisocial in Study 1 and was either control or prosocial in Study 2, and co-participant’s behavioral tendencies were either antisocial or prosocial in Studies 3 and 4.

**Figure 4.**
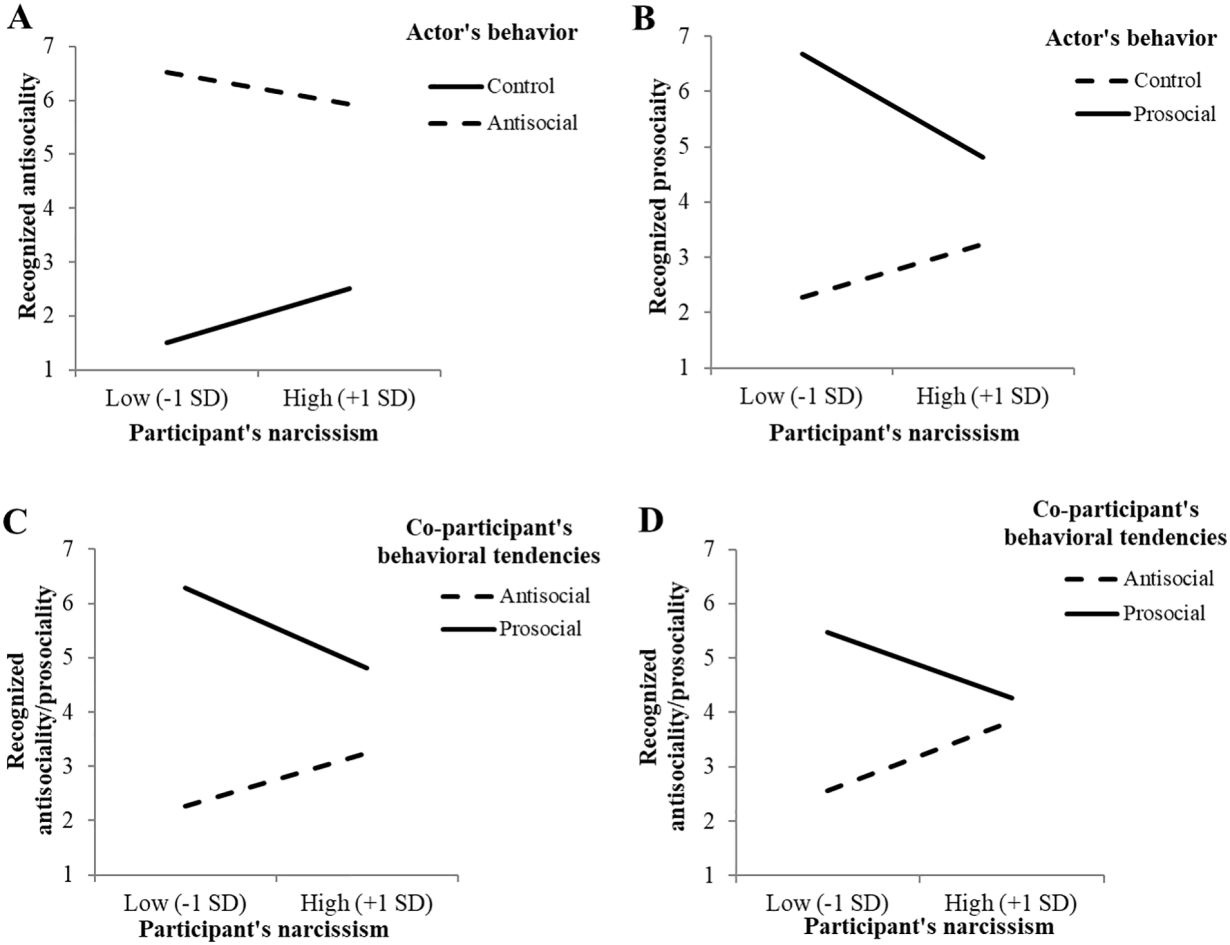
Effects on recognized antisociality/prosociality (Studies 1–4): Interaction between actor’s behavior and participant’s narcissism on observer’s recognized antisociality (A, Study 1) or recognized prosociality (B, Study 2), and interaction between co-participant’s behavioral tendencies and participant’s narcissism on observer’s recognized antisociality/prosociality (C, Study 3; D, Study 4); “high” and “low” narcissism refer to scores on the NPI scale that were 1 *SD* above the mean or 1 *SD* below the mean, respectively. Note. NPI = Narcissistic Personality Inventory.

Because self-relevance showed no significant effects, we excluded it in the following analyses. To test a mediated moderation model with recognized antisociality as the mediator (Figure 3A), we followed the procedure proposed by Preacher et al. (2007) in PROCESS. First. we tested the interaction between actor’s behavior and narcissism on recognized antisociality, which was found to be significant (Figure 4A). Compared with low narcissists, *B* = 4.96, *t*(440) = 35.76, *p* < .001, *r* = .86, 95% CI = [4.69, 5.23], high narcissists showed a smaller difference in recognized antisociality between the two conditions, *B* = 3.51, *t*(440) = 25.38, *p* < .001, *r* = .77, 95% CI = [3.23, 3.78].

Second, we examined the effect of recognized antisociality on moral character evaluation while controlling for actor’s behavior, narcissism, and their interaction. As expected, recognized antisociality negatively predicted moral character evaluation, *B* = −0.42, *t*(439) = −8.22, *p* < .001, *r* = .37, 95% CI = [–0.53, –0.32]. Third, we examined the indirect effect of actor’s behavior on moral character evaluation via recognized antisociality as a function of narcissism (Model 8), which was supported, *B* = 1.23, 95% CI = [0.78, 1.78].

## Discussion and Introduction to Study 2

The results of Study 1 provide support for the hypo-responsiveness hypothesis and the mediating effect of recognized antisociality. High (vs. low) narcissists recognized less antisocial behavior, which in turn led them to differentiate less between the antisocial and the control actor when making moral character evaluations. We did not find support for the competing hyper-responsiveness hypothesis and the moderating role of self-relevance. In Study 2, we aimed to conceptually replicate these findings for prosocial behavior. Considering that self-relevance showed no effects in Study 1, we excluded it in Study 2.

### Method

#### Participants

In total, 250 individuals from the United States (38.8% female; *M*_age_ = 33.89, *SD*_age_ = 10.76) were recruited through MTurk for US$2. One participant was excluded for exceeding the maximum given time (45 min).

#### Procedure

Participants were randomly allocated to either the control or the prosocial condition. The procedure was the same as in Study 1, except that the buffer measure only included the agreeableness and neuroticism components of the NEO-FFI.

#### Materials

##### Narcissism

We used the same NPI scale as in Study 1 (α = .96).

##### Manipulation of actor’s behavior

Actor’s behavior was manipulated by describing a scenario in which the actor (Person A) offered their place for the last movie ticket to another person (Person B) who had traveled from afar to see the movie (prosocial behavior) or did nothing (control behavior; see Figure 1B for a visualization and Supplemental Materials for further details).

##### Moral character evaluation

The same items were used as in Study 1 (α = .92).

##### Recognized prosociality

Four items, adapted from two altruism-related measures (Grant, 2008; International Personality Item Pool, 2001), were used to measure the degree to which participants recognized that the actor’s behavior was prosocial (e.g., “I think Person A was kind to Person B”; α = .92; 1 = *strongly disagree*, 7 = *strongly agree*).

##### Manipulation check

We used one item to assess the effectiveness of the manipulation, asking participants whether the actor (Person A) “offered Person B their place in the line to buy the last ticket” (1) or “did nothing and went back to talk with others” (0).

### Results

Descriptive statistics and correlations are presented in Table 1.

#### Manipulation check

A chi-square test showed that participants were more likely to indicate that the actor displayed a prosocial act in the prosocial condition (94.21%, *n* = 114 out of 121) than in the control condition (25.00%, *n* = 32 out of 128), χ^2^(1, *N* = 249) = 122.86, *p* < .001, ϕ = .70, 95% CI = [0.62, 0.79]. Thus, the manipulation was successful.^4^

#### Moral character evaluation

We used Model 1 in PROCESS to test the effects of actor’s behavior and narcissism on moral character evaluation (Table 3). The results revealed a significant main effect of actor’s behavior, with participants in the prosocial condition (*M* = 5.68, *SD* = 1.20) reporting higher moral character evaluations of the actor than those in the control condition (*M* = 4.21, *SD* = 1.46). Narcissism did not significantly predict moral character evaluation; however, the anticipated interaction was significant (Figure 2B). Both low narcissists, *B* = 1.93, *t*(245) = 8.17, *p* < .001, *r* = .46, 95% CI = [1.47, 2.40], and high narcissists, *B* = 1.02, *t*(245) = 4.29, *p* < .001, *r* = .26, 95% CI = [0.55, 1.48], rated the actor in the prosocial condition as more moral than the one in the control condition, but this difference was again smaller for high narcissists.

**Table 3.** Regression Results on Moral Character Evaluation and Recognized Antisociality/Prosociality (Studies 2–4).

Predictors	Moral character evaluation				Recognized antisociality/prosociality			
	*B* [95% CI]	*t* (*df*)	*p*	*r*	*B* [95% CI]	*t* (*df*)	*p*	*r*
Study 2								
Actor’s behavior	1.47 [1.14, 1.80]	8.82 (245)	<.001	.49	2.98 [2.69, 3.27]	20.43 (245)	<.001	.79
Narcissism	0.39 [–0.17, 0.95]	1.37 (245)	.172	.09	0.59 [–1.08, 0.10]	−2.36 (245)	.019	.15
Actor’s Behavior × Narcissism	−1.56 [–2.69, –0.44]	−2.73 (245)	.006	.17	−4.05 [–5.04, –3.06]	−8.09 (245)	<.001	.46
Study 3								
Co-participant’s behavioral tendencies	3.04 [2.68, 3.41]	16.33 (245)	<.001	.72	3.12 [2.84. 3.39]	22.43 (245)	<.001	.82
Narcissism	0.98 [0.32, 1.65]	2.93 (245)	.004	.18	−0.19 [–0.68, 0.30]	−0.76 (245)	.448	.05
Co-participant’s Behavioral Tendencies × Narcissism	−2.81 [–4.14, –1.49]	−4.19 (245)	<.001	.26	−4.20 [–5.19, –3.21]	−8.37 (245)	<.001	.47
Study 4								
Co-participant’s behavioral tendencies	1.17 [0.84, 1.49]	7.07 (238)	<.001	.42	1.67 [1.43, 1.91]	13.73 (238)	<.001	.66
Narcissism	1.61 [1.05, 2.17]	5.64 (238)	<.001	.34	0.07 [–0.34, 0.48]	0.34 (238)	.733	.02
Co-participant’s Behavioral Tendencies × Narcissism	−3.05 [–4.17, –1.93]	−5.36 (238)	<.001	.33	−4.29 [–5.11, –3.46]	−10.23 (238)	<.001	.55

*Note.* 95% CIs are shown in brackets. The effect size is represented by Pearson’s *r*. In Study 2, for actor’s behavior, control = 0, prosocial = 1; the mediator label was recognized prosociality. In Studies 3 and 4, for co-participant’s behavioral tendencies, antisocial tendencies = 0, prosocial tendencies = 1; the mediator label was recognized antisociality/prosociality. CI = confidence interval; *df* = degrees of freedom.

#### Mediated moderation model

We followed the same procedure as in Study 1 to test mediated moderation (Figure 3B). There was a significant interaction between actor’s behavior and narcissism on recognized prosociality (Table 3). Specifically, compared with low narcissists, *B* = 4.17, *t*(245) = 20.15, *p* < .001, *r* = .79, 95% CI = [3.76, 4.57], high narcissists displayed a smaller difference in recognized prosociality between the two conditions, *B* = 1.80, *t*(245) = 8.68, *p* < .001, *r* = .48, 95% CI = [1.39, 2.20] (Figure 4B).

The relationship between recognized prosociality and moral character evaluation when controlling for actor’s behavior, narcissism, and their interaction, was significant, *B* = 0.46, *t*(244) = 7.01, *p* < .001, *r* = .41, 95% CI = [0.34, 0.60]. The indirect effect of actor’s behavior on moral character evaluation through recognized prosociality as a function of narcissism was significant, *B* = −1.90, 95% CI = [–2.54, –1.28].

## Discussion and Introduction to Study 3

Study 2 findings were consistent with findings of Study 1. High (vs. low) narcissists showed less recognition of prosocial behavior versus control behavior, which explained their lower responsiveness in moral character evaluations. Thus, support for the hypo-responsiveness hypothesis extended from responses to antisocial behavior to responses to prosocial behavior.

So far, we focused on moral character evaluations to operationalize observers’ responsiveness to actors’ social behavior. In Study 3, we examine whether narcissists’ dampened responsiveness also manifests itself in behavior. To test this, we employed a Dictator Game (DG; Forsythe et al., 1994) to measure reward and a Voodoo Doll Task (VDT; DeWall et al., 2013) to measure punishment.

Finally, considering that in Studies 1 and 2 participants might think that one specific behavior was not sufficient to infer others’ moral character, in Study 3 we operationalized the actor’s behavioral tendencies as either antisocial or prosocial based on a series of behaviors, which also enabled a direct comparison of participants’ responsiveness toward antisocial and prosocial actors in one study. Moreover, we examined the mediating effect of recognized antisociality/prosociality on all three types of responses.

### Method

#### Participants

In total, 250 participants from the United States (42.8% female; *M*_age_ = 36.18, *SD*_age_ = 11.37) were recruited online via MTurk for US$2. One participant was excluded for spending longer than the given 45 min.

#### Procedure

Participants were randomly allocated to the antisocial or prosocial condition. Participants first completed several questionnaires, including demographics, NPI, self-report behavioral tendency (SRBT), and a buffer measure (same as in Study 2). Next, participants were informed that they would play a computer-mediated game with another randomly matched participant. To enhance psychosocial realism, participants were told that to help them get acquainted, one of the three questionnaires they had just completed would be randomly selected and exchanged with their co-participant. In fact, all participants were shown a profile of their alleged co-participant based only on the SRBT, indicating either antisocial or prosocial tendencies. Afterward, participants completed the measurements of moral character evaluations, reward, and punishment, followed by the manipulation check.

#### Materials

##### Narcissism

We used the same scale as in Studies 1 and 2 (α = .95).

##### SRBT questionnaire

This questionnaire was developed to manipulate the co-participant’s prosocial versus antisocial tendencies and included 10 items adapted from the self-report altruism scale (Rushton et al., 1981) to indicate the extent to which individuals would engage in various behaviors (1 = *extremely unlikely*, 5 = *extremely likely*). Five items contained antisocial (e.g., “I will gossip about people I don’t like”) and five items contained prosocial (e.g., “I will donate money to a charity for the homeless”) behavioral tendencies.

##### Manipulation of co-participant’s behavioral tendencies

In the antisocial condition, the co-participant scored higher on the SRBT antisocial items and lower on the prosocial items, whereas the co-participant in the prosocial condition had the opposite scoring trend. The scores were balanced to ensure that the degree of prosocial tendencies was the same as that of antisocial tendencies.

##### Recognized antisociality/prosociality

Eight items were adapted from Studies 1 and 2 to measure the degree to which participants perceived the co-participant’s behavioral tendencies to be antisocial or prosocial (e.g., “I think my co-participant is helpful to others”; α = .95; 1 = *strongly disagree*, 7 = *strongly agree*). Recognized antisociality/prosociality was calculated as an average after reverse-coding the antisocial items, with higher scores indicating recognized prosociality (vs. antisociality).

##### Moral character evaluation

The same items were used as in previous studies (α = .96).

##### Reward

The widely used DG was employed to assess participants’ reward behavior (Ruffle, 1998). Participants’ task was to divide 20 lottery tokens between themselves and the co-participant. The more lottery tokens one ended up with, the greater one’s chances of winning a prize. Thus, giving more tickets to the co-participant reflects greater reward behavior. Because the distribution of the given lottery tokens was bimodal, with the majority of participants giving either 10 tokens (34.14%) or 0 (29.72%), following previous research the number of given tokens was dichotomized into high reward (giving 10 or more tokens = 1) and low reward (giving fewer than 10 tokens = 0; for example, Fetchenhauer & Huang, 2004), with 50.20% and 49.80% participants falling in the high- and low-reward categories, respectively.

##### Punishment

The broadly used and highly reliable and valid VDT, which allows participants to stick pins into a doll representing someone else, was administrated to measure punishment behavior (DeWall et al., 2013; Øverup et al., 2017). The law of similarity (Rozin et al., 1986) suggests that the process of harming a voodoo doll by sticking pins into it is psychologically similar to the process of actually harming the person the doll represents. Therefore, despite that pin insertion does not directly inflict harm on others and captures symbolic aggression (Golec de Zavala et al., 2019), it is associated with various indicators of actual aggression, like trait physical and psychological aggression (DeWall et al., 2013).

Participants could choose to stick between 0 and 51 pins into an outline of a doll representing their co-participant, with more pins representing greater punishment. Given that pin usage constituted a count variable that was over-dispersed (*M* = 10.38 < variance = 285.09) and zero-inflated (60.64% of participants chose zero pins), a zero-inflated negative binomial (ZINB) regression model was used in the analysis (Atkins & Gallop, 2007). This model showed a good fit^5^ and has been used in previous research employing the VDT (Øverup et al., 2017). The ZINB regression model is comprised of two stages. The first model is a binary logistic (BL) regression model that predicts the occurrence of zero pins versus other outcomes (i.e., 0 pins = 0, non-punishment vs. 1–51 pins = 1, punishment); the second model is a negative binomial (NB) regression model, which predicts the frequency of pins among participants who chose to stick at least one pin (i.e., ranging from 1 to 51). As such, it essentially divides participants’ responses into two components: (a) whether they punished the co-participant or not, and (b) the degree of punishment among those who chose to punish.

##### Manipulation check

Participants indicated their general impression of their co-participant by choosing between two options: “Egoistic, unhelpful, and unconcerned with the welfare of others” (0) or “Altruistic, helpful, and concerned with the welfare of others” (1).

### Results

Descriptive statistics and correlations are presented in Table 4.

**Table 4.** Means, Standard Deviations, and Correlations Between Variables (Studies 3 and 4).

Variable	*M*_S3_	*SD*_S3_	1	2	3	4	5	6	*M*_S4_	*SD*_S4_
1. Co-participant’s behavioral tendencies	0.49	0.50	—	.002	.60**	.38**	.08	−.14*	0.50	0.50
2. Narcissism	0.43	0.28	.06	—	−.03	.28*	.23**	.45**	0.57	0.29
3. Recognized antisociality/prosociality	3.92	1.99	.78**	.01	—	.57**	.12^a^	−.21**	4.03	1.41
4. Moral character evaluation	4.35	2.17	.71**	.16*	.85**	—	.42**	.10	5.14	1.53
5. Reward	6.73	5.71	.34**	.32**	.35**	.49**	—	.46**	11.00	5.60
6. Punishment^b^	10.38	16.89	−.12	.47**	−.26**	−.11	.31**	—	18.74	17.53

*Note.* Study 3 (*N* = 249) correlations are presented below the diagonal and Study 4 (*N* = 242) correlations are presented above the diagonal. In both studies, co-participant’s behavioral tendencies were dummy coded (antisocial tendencies = 0, prosocial tendencies = 1).

a In Study 4, recognized antisociality/prosociality was positively correlated with dichotomous reward (*r* = .20, *p* = .001). ^b^In both studies, the correlation coefficients between punishment and other variables are Spearman’s rank correlation coefficients due to the non-normal distribution of punishment.

* *p* < .05. **p < .01.

#### Manipulation check

A chi-square test showed that participants in the prosocial condition (96.69%, *n* = 117 out of 121) were more likely to indicate that their co-participant was prosocial/altruistic than were those in the antisocial condition (20.31%, *n* = 26 out of 128), χ^2^(1, *N* = 249) = 148.43, *p* < .001, ϕ = .77, 95% CI = [0.70, 0.85]. Thus, the manipulation was successful.^6^

#### Moral character evaluation

We used Model 1 in PROCESS to test the effects of the co-participant’s behavioral tendencies and narcissism on moral character evaluation (Table 3). This revealed a significant main effect of behavioral tendencies, with participants in the prosocial condition (*M* = 5.93, *SD* = 1.09) reporting higher moral character of the co-participant than those in the antisocial condition (*M* = 2.85, *SD* = 1.86). Narcissism was positively related to moral character evaluation. Importantly, the anticipated interaction was significant (Figure 2C). Compared with low narcissists, *B* = 3.82, *t*(245) = 14.48, *p* < .001, *r* = .68, 95% CI = [3.30, 4.34], high narcissists differentiated less between the prosocial and antisocial conditions in their moral character evaluations, *B* = 2.26, *t*(245) = 8.59, *p* < .001, *r* = .48, 95% CI = [1.74, 2.78].

#### Reward

**Figure 5.**
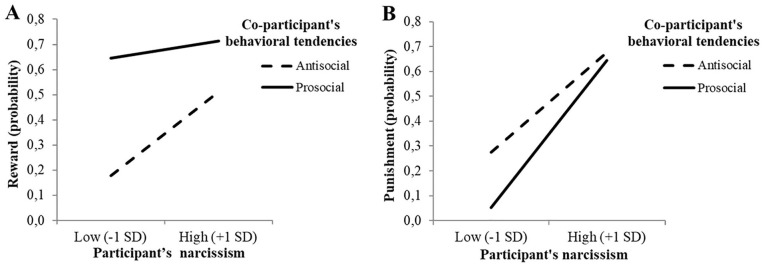
Responsiveness on reward and punishment (Study 3): Interaction between co-participant’s behavioral tendencies and participant’s narcissism on reward to (A) and punishment of (B) the co-participant (Study 3); “high” and “low” narcissism refer to scores on the NPI scale that were 1 *SD* above the mean or 1 *SD* below the mean, respectively. *Note.* NPI = Narcissistic Personality Inventory.

The results of Model 1 (PROCESS) revealed a significant main effect of behavioral tendencies on reward (Table 5), such that participants in the prosocial condition (68.60%, *n* = 83 out of 121) were 4.53 times more likely to offer a high (vs. low) reward to the co-participant than those in the antisocial condition (32.81%, *n* = 42 out of 128). Narcissism was found to positively predict reward, which was qualified by a significant interaction (Figure 5A). The odds ratio showed that low narcissists were 8.32 times more likely to offer their co-participant a high (vs. low) reward in the prosocial condition than in the antisocial condition, *B* = 2.12, *p* < .001, 95% CI = [1.30, 2.94], whereas high narcissists were only 2.57 times more likely to do so, *B* = 0.94,*p* = .014, 95% CI = [0.19, 1.70].

**Table 5.** Regression Results on Reward and Punishment (Studies 3 and 4).

Predictors	Reward			Punishment (BL)			Punishment (NB)		
	*B* [95% CI]	*p*	OR	*B* [95% CI]	*p*	OR	*B* [95% CI]	*p*	RR
Study 3									
Co-participant’s behavioral tendencies	1.51 [0.97, 2.06]	<.001	4.53	−0.87 [−1.46, −0.28]	.004	0.42	0.01 [−0.42, 0.44]	.958	0.99
Narcissism	1.49 [0.48, 2.49]	.004	1.51	3.89 [2.74, 5.03]	<.001	2.94	1.06 [0.22, 1.91]	.014	2.90
Co-participant’s Behavioral Tendencies × Narcissism	−2.12 [−4.13, −0.10]	.042	0.56	2.89 [0.41, 5.37]	.022	2.23	−0.11 [−2.05, 1.83]	.909	0.89
Study 4									
Co-participant’s behavioral tendencies	0.59 [0.01, 1.17]	.045	1.81	−0.95 [−1.63, −0.27]	.006	0.39	−0.03 [0.34, −0.29]	.862	0.97
Narcissism	0.17 [0.81, 1.15]	.729	1.05	4.96 [3.65, 6.28]	<.001	4.27	0.27 [−0.42, 0.96]	.444	1.31
Co-participant’s Behavioral Tendencies × Narcissism	−1.00 [−2.98, 0.98]	.320	0.75	0.70 [−1.94, 3.34]	.604	1.23	−0.15 [−1.53, 1.23]	.833	0.86

*Note.* 95% CIs are shown in brackets. The effect size is represented by either OR or RR. In Studies 3 and 4, for co-participant’s behavioral tendencies, antisocial tendencies = 0, prosocial tendencies = 1. Punishment (BL) was based on the BL regression model, while punishment (NB) was based on the NB regression model of the ZINB regression model. Because ORs are scale dependent, the ORs were calculated using standardized predictors. BL = binary logistic; NB = negative binomial; CI = confidence interval; OR = odds ratio; RR = rate ratio; ZINB = zero-inflated negative binomial.

#### Punishment

We ran the ZINB regression in R to examine the effects of the co-participant’s behavioral tendencies and narcissism on punishment (Table 5). The BL regression, dichotomizing punishment, revealed a significant main effect of the behavioral tendencies, with participants in the antisocial condition (46.09%, *n* = 59 out of 128) being 2.39 times^7^ more likely to punish the co-participant than participants in the prosocial condition (32.23%, *n* = 39 out of 121). The main effect of narcissism was also significant, which was again qualified by a significant interaction (Figure 5B). Low narcissists were 6.41 times more likely to punish the co-participant in the antisocial condition than in the prosocial condition, *B* = −1.86, *p* = .001, 95% CI = [–2.94, –0.77], whereas high narcissists displayed no difference in punishment likelihood between the two conditions, *B* = −0.26, *p* = .525, 95% CI = [–1.04, 0.53].

The NB regression model, with participants who punished (i.e., sticking 1–51 pins, *n* = 98), revealed that narcissism positively predicted punishment. The main effect of behavioral tendencies and the interaction effect were not significant (Table 5). The non-significant main effect of behavioral tendencies could be due to the fact that the majority of the 98 participants were high narcissists who indiscriminately punished both antisocial and prosocial co-participants.

Taken together, high and low narcissists’ difference in responsiveness regarding punishment was mainly reflected in their decision to punish or not (BL regression model) rather than in the degree of punishment (NB regression model).

#### Mediated moderation models

We investigated whether participants’ recognized antisociality/prosociality could explain their moral character evaluation, reward, and punishment responses using Model 8 in PROCESS (Figure 3C).^8^ A significant interaction effect was found between behavioral tendencies and narcissism on recognized antisociality/prosociality (Table 3; Figure 4C). Compared with low narcissists, *B* = 4.28, *t*(245) = 21.75, *p* < .001, *r* = .81, 95% CI = [3.89, 4.67], high narcissists displayed a smaller difference in recognized antisociality/prosociality between the two conditions, *B* = 1.95, *t*(245) = 9.93, *p* < .001, *r* = .54, 95% CI = [1.56, 2.34]. Recognized antisociality/prosociality positively predicted moral character evaluation, *B* = 0.90, *t*(244) = 14.20, *p* < .001, *r* = .67, 95% CI = [0.78, 1.03], and reward (*B* = 0.37, *p* = .004, odds ratio = 1.45, 95% CI = [0.12, 0.63]), and negatively predicted punishment (*B* = −0.60, *p* < .001, odds ratio = 0.55, 95% CI = [–0.88, –0.32]) when controlling for co-participants’ behavioral tendencies, narcissism, and their interaction. The indirect effect of behavioral tendencies on the three responses through recognized antisociality/prosociality as a function of narcissism was significant for moral character evaluation (*B* = −3.79, 95% CI = [–5.00, –2.66]), reward (*B* = −1.57, 95% CI = [–3.00, –0.45]), and punishment (*B* = 2.51, 95% CI = [1.21, 4.26]), supporting mediated moderation.

## Discussion and Introduction to Study 4

Study 3 replicated the results of the previous studies regarding moral character evaluation and extended the responsiveness effects to behavioral indices of reward and punishment. Further supporting the hypo-responsiveness hypothesis, narcissists were less responsive to antisocial versus prosocial others when evaluating their moral character as well as in their reward behavior, and they showed no apparent discrimination between antisocial and prosocial others in their punishment, which was explained by their lower discrimination on recognized antisociality/prosociality.

Despite the consistent findings across three studies, a potential alternative explanation could be that narcissists’ hypo-responsiveness stemmed from their inattentiveness to study instructions. To exclude this explanation, we conducted a pre-registered study replicating Study 3, in which we (a) added a monetary incentive to motivate participants to read the manipulation carefully, (b) recorded the time participants spent on the manipulation page as a proxy of attention devoted to the manipulation,^9^ and (c) added an attention check question to enable removing inattentive participants. The pre-registered document can be found at http://aspredicted.org/blind.php?x=v8ku55.

### Method

#### Participants

In total, 253 participants from the United States (34.8% female; *M*_age_ = 35.52, *SD*_age_ = 9.19) were recruited online via MTurk for US$3. All participants completed the study within the given 60 min, which was longer than the limit in Study 3 due to the inclusion of additional measures for exploratory purposes. Eleven participants were excluded for indicating that we should not use their data on the attention check.

#### Procedure

The procedure was the same as in Study 3, except that we included the NARQ to explore the potentially different effects of its two dimensions on participants’ behavioral responses.^10^ Furthermore, we used a cognitive task as the buffer task instead of the NEO-FFI, because the agreeableness and extraversion dimensions of NEO-FFI are correlated with narcissism (Paulhus & Williams, 2002) and may therefore not be fully effective as a buffer. Finally, participants completed the attention check.

#### Materials

All materials were the same as in Study 3.

##### Narcissism

The NPI was reliable (α = .95).

##### Buffer task

Participants were asked to count backward by subtracting three for 30 s, beginning with the number 101 (i.e., 101, 98, 95, etc.; Stavrinos et al., 2011).

##### Manipulation of co-participant’s behavioral tendencies

This was the same as in Study 3, except that the instruction included a monetary incentive, which provided a chance of receiving $5 for good performance on a quiz about the manipulation content.

##### Recognized antisociality/prosociality

A shortened scale including four out of eight items from Study 3 was used to compensate for additional measures included for exploratory purposes (α = .71).^11^

##### Moral character evaluation

The scale was reliable (α = .89).

##### Reward

As in Study 3, the number of given tokens was dichotomized into low reward (0; 27.27% of participants) and high reward (1; 72.73% of participants).

##### Punishment

We again used the ZINB regression model due to over-dispersion (*M* = 18.74 < variance = 307.46) and zero-inflation (31.40% participants stuck no pins) of the data, and its better fit with the data.^12^

##### Manipulation check

Same as in Study 3.

##### Attention check

After completing two questions about their effort and attention paid to the study (used to help them answer the final question), participants answered the attention check question: “In your honest opinion, should we use your data in our analyses in this study” (“No” or “Yes”), with participants responding “No” being excluded from data analysis (Meade & Craig, 2012).

### Results

Table 4 presents descriptive statistics and correlations. Unless indicated, the analyses used are the same as in Study 3.

#### Manipulation check

Participants in the prosocial condition (94.21%, *n* = 114 out of 121) were more likely to indicate that their co-participant was prosocial/altruistic than were those in the antisocial condition (57.02%, *n* = 69 out of 121), χ^2^(1, *N* = 242) = 45.39, *p* < .001, ϕ = .43, 95% CI = [0.34, 0.53]. Thus, the manipulation was successful.

#### Moral character evaluation

The results (Table 3) showed a significant main effect of behavioral tendencies, with participants in the prosocial condition rating their co-participant as more moral (*M* = 5.73, *SD* = 1.00) than those in the antisocial condition (*M* = 4.56, *SD* = 1.74). The main effect of narcissism was also significant and qualified by a significant interaction (Figure 2D). Low narcissists rated their co-participant in the prosocial condition as more moral than the one in the antisocial condition, *B* = 2.06, *t*(238) = 8.79, *p* < .001, *r* = .50, 95% CI = [1.60, 2.53], while the difference was not significant for high narcissists, *B* = 0.28, *t*(238) = 1.18, *p* = .241, *r* = .08, 95% CI = [–0.19, 0.74].

#### Reward

The results revealed a significant main effect of behavioral tendencies, such that participants in the prosocial condition (78.51%, *n* = 95 out of 121) were 1.80 times more likely to offer a high (vs. low) reward to their co-participant than those in the antisocial condition (66.94%, *n* = 81 out of 121). The main effect of narcissism and the interaction were non-significant (Table 5).

#### Punishment

The results of the BL regression model (Table 5) revealed a significant main effect of behavioral tendencies, with participants in the antisocial condition (76.03%, *n* = 92 out of 121) being 2.58 times more likely to punish their co-participant than those in the prosocial condition (61.16%,*n* = 74 out of 121). Narcissism positively predicted punishment. However, the interaction was not significant. The results of the NB regression model (Table 5) showed that the main effects of behavioral tendencies and narcissism and their interaction were not significant.

These results indicate that the interaction between narcissism and other’s behavioral tendencies on moral character evaluation replicated again, but the interaction on reward and punishment did not. Although there was no significant overall interaction effect on reward and punishment, there could still be an indirect effect via recognized antisociality/prosociality (Zhao et al., 2010).

#### Mediated moderation models

We tested the indirect effects using Model 8 in PROCESS with moral character evaluation, reward, and punishment as the outcomes (Figure 3C).^13^ The first step yielded a significant interaction effect between the co-participant’s behavioral tendencies and narcissism on recognized antisociality/prosociality (Table 3; Figure 4D). Specifically, compared with low narcissists, *B* = 2.93, *t*(238) = 16.93, *p* < .001, *r* = .74, 95% CI = [2.59, 3.27], high narcissists displayed a smaller difference in recognized antisociality/prosociality between the two conditions, *B* = 0.42, *t*(238) = 2.41, *p* = .017, *r* = .15, 95% CI = [0.08, 0.76].

Recognized antisociality/prosociality positively predicted moral character evaluation, *B* = 0.54, *t*(237) = 6.57, *p* < .001, *r* = .39, 95% CI = [0.37, 0.69], and reward (*B* = 0.36, *p* = .024, odds ratio = 1.43, 95% CI = [0.05, 0.67]), and negatively predicted punishment (*B* = −0.66, *p* < .001, odds ratio = 0.51, 95% CI = [–1.02, –0.31]) when controlling for co-participants’ behavioral tendencies, narcissism, and their interaction. The indirect effect of behavioral tendencies on three types of responses through recognized antisociality/prosociality as a function of narcissism was significant for moral character evaluation (*B* = −2.29, 95% CI = [–3.22, –1.47]), reward (*B* = −1.54, 95% CI = [–3.18, –0.12]), and punishment (*B* = 2.85, 95% CI = [0.78, 5.77]). This indicates that the interaction between narcissism and co-participant’s behavioral tendencies indirectly affected moral character evaluations, reward, and punishment via recognized antisociality/prosociality.

### Discussion of Study 4

Pre-registered Study 4 replicated the findings of the previous three studies regarding moral character evaluation, further confirming narcissists’ hypo-responsiveness. Although narcissists’ hypo-responsiveness did not become manifest in overall effects on reward and punishment, we found an indirect effect via attenuated recognition of antisociality/prosociality. Specifically, narcissists’ dampened recognition of behavioral tendencies as either prosocial or antisocial explained their lower responsiveness in terms of moral character evaluation, reward, and punishment. Importantly, after including the monetary incentive and removing potentially inattentive participants, the results of Study 4 help to rule out the possibility that narcissists’ hypo-responsiveness stemmed from their inattentiveness.

## General Discussion

We examined how observers’ narcissism shapes their responsiveness to others’ social behavior. Across four studies, narcissists were consistently less responsive to variations in actors’ antisocial or prosocial behavior, providing evidence for a hypo-responsiveness rather than a hyper-responsiveness hypothesis. Specifically, narcissists differentiated less between others’ antisocial versus control behavior (Study 1), others’ prosocial versus control behavior (Study 2), and others’ antisocial versus prosocial tendencies (Studies 3 and 4), which was reflected in their subsequent moral character evaluations (Studies 1–4), and reward and punishment behavior (Studies 3 and 4).

### Theoretical and Practical Implications

The present research has several theoretical implications. First, it extends prior research on narcissists’ responses to others’ behavior by switching from the perspective of a direct target or victim of (close) others’ behavior (Back et al., 2013; Bushman & Baumeister, 1998) to an indirect target or third-party observer perspective, examining responses to both antisocial and prosocial behaviors, and identifying downstream consequences of narcissists’ hyposensitivity mainly for moral character evaluations and also indirectly for reward and punishment. Therefore, our findings improve our understanding of narcissists’ dynamic self-regulatory processing in interpersonal situations (Morf & Rhodewalt, 2001) from more inclusive perspective.

Previous work has shown that, to maintain a positive self-concept in the agentic (vs. communal) domain (e.g., power, status; Grijalva & Zhang, 2016), narcissists are hyper-sensitive and vigilant to external cues related to status or power (Grapsas et al., 2020). Our findings on the mediation effects of recognized antisociality/prosociality complement this work by illuminating narcissists’ lower sensitivity to or recognition of communal information. Moreover, our exploratory results showing narcissists’ differentiation in perceived similarity to a successful/unsuccessful target (agentic information) provided further evidence for narcissists’ higher sensitivity to agentic than communal information (see detailed results in Supplemental Materials). Thus, it does not appear that narcissists are indiscriminately less sensitive to all contexts.

Alternatively, narcissists’ hypo-responsiveness could stem from their awareness of others’ underlying motivations for antisocial and prosocial behaviors. Both antisocial and prosocial behaviors can constitute a route to positive self-presentation (Flynn et al., 2006; Van Kleef et al., 2011), with antisocial behaviors being more commonly adopted by narcissists to gain status or attention (Adams et al., 2014). Although narcissism is unrelated to self-enhancement through prosocial behaviors (Nehrlich et al., 2019), narcissists sometimes present prosocial behaviors for selfish reasons, like gaining career experience (Brunell et al., 2014), or for praise and attention (Konrath et al., 2016). Thus, it is possible that narcissists are less responsive to others’ prosocial behaviors because they are aware of others’ potentially selfish motivations, and show greater tolerance for others’ antisocial behaviors which they themselves use to gain attention or status. Our exploratory results (see Supplemental Materials) showed that narcissists’ hypo-responsiveness on moral character evaluation was related to their lower self-reported antisociality/prosociality. One might posit that narcissists’ hypo-responsiveness resulted from them perceiving relatively lower (higher) similarity with the prosocial (antisocial) target. However, we found that narcissists showed no difference in perceived similarity with the two targets, which could be another manifestation of their insensitivity. Nonetheless, further examining the role of similarity in the scope of narcissists’ responses to others is a fruitful avenue for future research.

Interestingly, self-relevance was not found to play a role in affecting narcissists’ responsiveness in Study 1, with narcissists’ hypo-responsiveness being observed across both high and low self-relevance conditions. The fact that the antisocial actor pushed in at the front of the queue rather than immediately in front of participants might have rendered this behavior less psychologically proximate and less salient despite being relatively self-relevant, removing it from narcissists’ radar and reducing the need to allocate cognitive resources to encode this behavior (Wise et al., 2009). Consequently, such behavior may not have been perceived as a personal affront by narcissists (Lustman et al., 2010), reducing its perceived threat to their self-concept. Thus, this finding suggests that narcissists ignore threatening information that is not explicitly directed at them. Given that Back et al. (2013) did report that narcissists show revenge-related reactions when directly harmed by close others (i.e., friends), future research could examine the degree to which the anti- or prosocial behavior is directly aimed toward the narcissist while also considering the specific relationship between the narcissist and the protagonist.

Our findings that narcissists punished more overall regardless of their co-participant’s behavioral tendencies also contribute to research on narcissists’ unprovoked aggression (Park & Colvin, 2015; Reidy et al., 2010). Narcissists’ greater punishment of others might reflect their desire to assert their dominance vis-à-vis the other participant.

In terms of practical implications, our findings indicate that narcissists respond less discriminately on rewarding and punishing antisocial versus prosocial behaviors, which may over time lead to an increase in antisocial behaviors and a decrease in prosocial behaviors (Fehr & Fischbacher, 2004; Henrich et al., 2005). Such potential adverse influences may be particularly disconcerting when narcissists occupy influential positions. Recent research showed that narcissistic leaders sanctioned integrity-norm violators less and were associated with organizational cultures that devalued integrity (O’Reilly et al., 2018). Considering that narcissists have a higher chance of rising to powerful positions (Nevicka et al., 2011), organizations should introduce clear principles of conduct combined with incentives and penalties that are independent of leaders’ decisions to reduce the potentially detrimental impact of such leaders on organizations’ moral climate.

### Strengths, Limitations, and Suggestions for Future Research

Our research has several strengths. We used different antisocial and prosocial behaviors and tendencies to demonstrate the generalizability of narcissists’ hypo-responsiveness to others’ social behavior and consistently found narcissists’ hypo-responsiveness in moral character evaluation. Furthermore, our findings show a similar effect for reward and punishment in Study 3, further lending some support for narcissists’ hypo-responsiveness. Finally, we illuminated underlying mechanisms by establishing recognition of others’ antisociality/prosociality as a mediator of narcissists’ moral character evaluations, reward, and punishment.

This research also has some limitations. Despite the validity and wide usage of the VDT (DeWall et al., 2013; Øverup et al., 2017), participants’ engagement in punishing may be affected by not seeing the consequences of their punishment behavior. Therefore, it would be helpful to enhance participants’ engagement in behavioral responses by adopting more direct punishment measures, such as noise blasts (Bushman & Baumeister, 1998). Moreover, because this was a one-shot study and there was little reason for participants to believe that the responses would affect their co-participant’s future behaviors, participants’ behavior toward their co-participant was unlikely to involve their conscious desire to regulate the co-participant’s future behavior. Future research could examine situations where punishment and reward behavior can be seen to have more observable impact on others over time.

While our research focused on im(morality) in the communal domain, future research could examine how narcissists, as third-party observers, respond to others’ (in)justices in the agentic domain that could harm or benefit someone else’s striving for status or power. For example, how would narcissists respond to seeing someone cheating in an examination, or seeing someone giving a classmate a leg up? Because narcissists’ higher feelings of power may allow them to better distinguish goal-relevant versus goal-irrelevant information (Guinote, 2007), they may categorize status- or power-related information as irrelevant if such information does not affect their own status or power. Therefore, they may be less responsive to such irrelevant information in spite of its status or power component. Thus, narcissists as a third party may likewise demonstrate hypo-responsiveness to others’ (in)justices in the agentic domain.

## Conclusion

The current research revealed that individuals high in narcissism are less responsive to variations in others’ social behaviors than are their low-narcissistic counterparts. This novel finding complements previous research by illuminating how narcissists respond to others’ antisocial and prosocial behaviors in terms of moral character evaluations and associated tendencies to punish or reward others. Given that narcissists are apparently inclined to respond less discriminately on evaluating, rewarding, and punishing antisocial versus prosocial behaviors, narcissists (especially in leadership positions) may contribute to the erosion of social norms that sustain community functioning.

## Supplemental Material

sj-docx-1-psp-10.1177_01461672211007293 – Supplemental material for How Narcissism Shapes Responses to Antisocial and Prosocial Behavior: Hypo-Responsiveness or Hyper-Responsiveness? Click here for additional data file. Supplemental material, sj-docx-1-psp-10.1177_01461672211007293 for How Narcissism Shapes Responses to Antisocial and Prosocial Behavior: Hypo-Responsiveness or Hyper-Responsiveness? by Jiafang Chen, Barbara Nevicka, Astrid C. Homan and Gerben A. van Kleef in Personality and Social Psychology Bulletin

sj-docx-2-psp-10.1177_01461672211007293 – Supplemental material for How Narcissism Shapes Responses to Antisocial and Prosocial Behavior: Hypo-Responsiveness or Hyper-Responsiveness? Click here for additional data file. Supplemental material, sj-docx-2-psp-10.1177_01461672211007293 for How Narcissism Shapes Responses to Antisocial and Prosocial Behavior: Hypo-Responsiveness or Hyper-Responsiveness? by Jiafang Chen, Barbara Nevicka, Astrid C. Homan and Gerben A. van Kleef in Personality and Social Psychology Bulletin

## Notes

1. The results of the Narcissistic Admiration and Rivalry Questionnaire (NARQ) dimensions and the Narcissistic Personality Inventory (NPI) showed similar patterns on responsiveness. See detailed results in the Supplemental Materials.

2. To keep the whole scale together and allow for exploratory analyses, we also included measures of sociable (i.e., “likable,” “warm,” “friendly”) and competent character evaluations (i.e., “competent,” “intelligent,” “skilled”). Results for all studies are reported in the Supplemental Materials.

3. The results remain the same when analyzing the data without participants who failed the manipulation check (see Supplemental Materials).

4. After excluding participants who failed the manipulation check, the interaction effect between actor’s behavior and observer’s narcissism on moral character evaluation became non-significant. However, the indirect effect on moral character evaluation through recognized prosociality remained significant. As such, the pattern of results remains generally consistent. See detailed results in Supplemental Materials.

5. The Vuong non-nested hypothesis test showed that a zero-inflated negative binomial (ZINB) regression model (θ = 1.45, log likelihood = −545.40) fitted the data better than a comparable negative binomial regression model (θ = 0.16, log likelihood = −1,183.91), *z* = 4.28, *p* < .001.

6. In Studies 3 and 4, the manipulation check constituted a more subjective evaluation and as such was not used for excluding participants.

7. The odds ratio was 0.42 (i.e., odds of punishment in the prosocial condition compared with the antisocial condition) and was translated to its reciprocal 1/0.42 (i.e., odds of punishment in the antisocial condition compared with the prosocial condition) for interpretation convenience. The same translation applies to other results for punishment.

8. We only focused on dichotomous punishment (i.e., binary logistic [BL] regression model) and not on count punishment (i.e., NB regression model) as the latter was not significantly correlated with recognized antisociality/prosociality, *r* = .10, *p* = .339, *n* = 98.

9. Narcissism was not significantly correlated with time spent on the manipulation page, *r* = −.02, *p* = .753, providing no evidence that narcissists paid less attention to the instructions.

10. The results of the NARQ and the NPI regarding three types of responses were similar (see Supplemental Materials).

11. We also measured participants’ perceived similarity with their co-participant as an alternative mediator. Results showed that high narcissists perceived no differentiation in similarity across two conditions, suggesting their insensitivity in perceived similarity (but not in an agentic context, which we also included as an exploratory condition). See results in the Supplemental Materials.

12. The Vuong non-nested hypothesis test results showed that a ZINB regression model (θ = 2.50, log likelihood = −799.20) fitted the data better than a negative binomial regression model (θ = 0.45, log likelihood = −1,785.51), *z* = 6.85, *p* < .001.

13. We only focused on dichotomous punishment (i.e., BL regression model) and not on count punishment (i.e., NB regression model) because the latter did not correlate with recognized antisociality/prosociality, *r* = .01, *p* = .856, *n* = 166.
