# Supplementary material for: How Narcissism Shapes Responses to Antisocial and Prosocial Behavior: Hypo-Responsiveness or Hyper-Responsiveness?
Source: Pers Soc Psychol Bull. 2021 Apr 15;48(3):363–81. doi: 10.1177/01461672211007293 (PMC8855397; doi:10.1177/01461672211007293)
Supplement: sj-docx-1-psp-10.1177_01461672211007293 – Supplemental material for How Narcissism Shapes Responses to Antisocial and Prosocial Behavior: Hypo-Responsiveness or Hyper-Responsiveness? [file sj-docx-1-psp-10.1177_01461672211007293.docx]

**Methods Reporting**

**Study 1**

The text reported in **bold** below has been added for explanatory purpose and was not presented to the participants.

**Independent Variables**

**Narcissism**: Please indicate whether the following statements apply to you by indicating either 'True' or 'False'. Work quickly through the questions and give the first response that comes to mind for each one.

**Narcissistic Personality Inventory (NPI; full scale available here:** <http://dx.doi.org/10.1037/0022-3514.54.5.890>)

**Manipulation of actor’s behavior (antisocial behavior vs. control behavior) and self-relevance (high self-relevance vs. low self-relevance):** Now we will provide you with a description of a hypothetical situation. As you are reading this description, try to imagine that you are actually in that situation. Visualize what this situation would look like…

Imagine that you have waited long and eagerly for a popular movie to come out in cinemas. You decide to watch the movie on its release day. Unfortunately, you arrive at the cinema late. There are still many people standing in a queue waiting to buy tickets, and there is another queue for snacks. The movie tickets may have been sold out when it is your turn because of its popularity, but you still want to see whether you are lucky enough to get one. You line up at the end of the queue for tickets and hope that it moves quickly. A few minutes later, you are pleased to see that there are only 4 people left in front of you. Looks like you will make the movie!

***Antisocial and high self-relevance condition***: At this moment, you see a person (indicated by the red circle) pushing in at the front of your queue for tickets (see below).


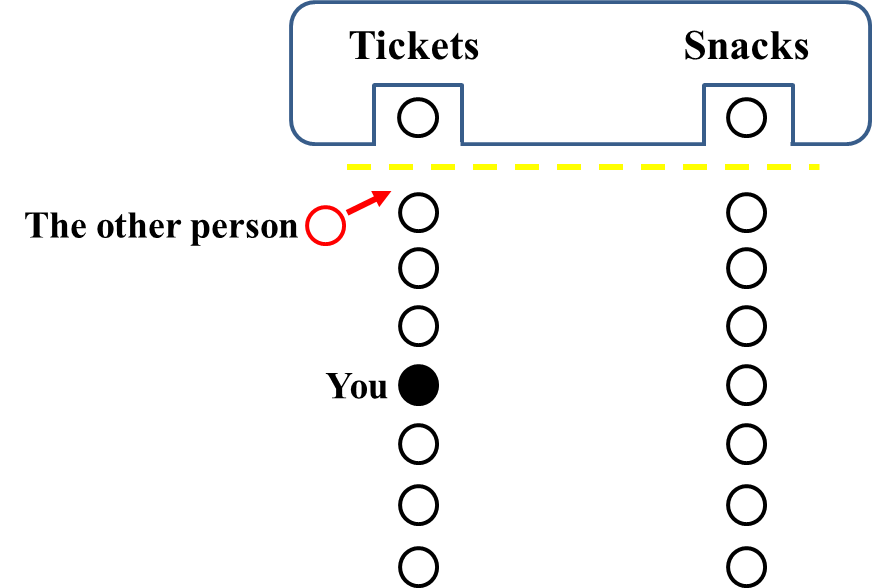


***Antisocial and low self-relevance condition***: At this moment, you see a person (indicated by the red circle) pushing in at the front of the queue for snacks (see below).


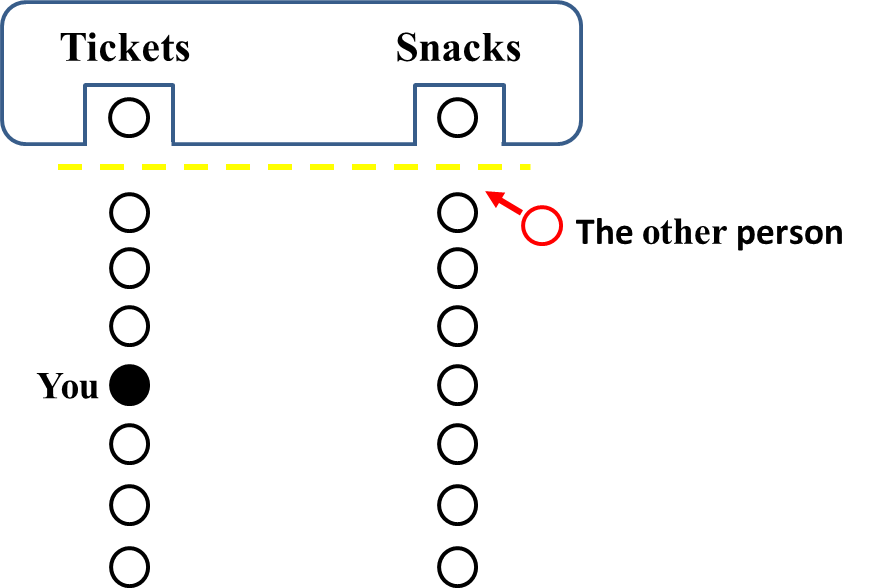


***Control and high self-relevance condition*:** At this moment, you see a person (indicated by the red circle) lining up at the end of your queue for tickets (see below).
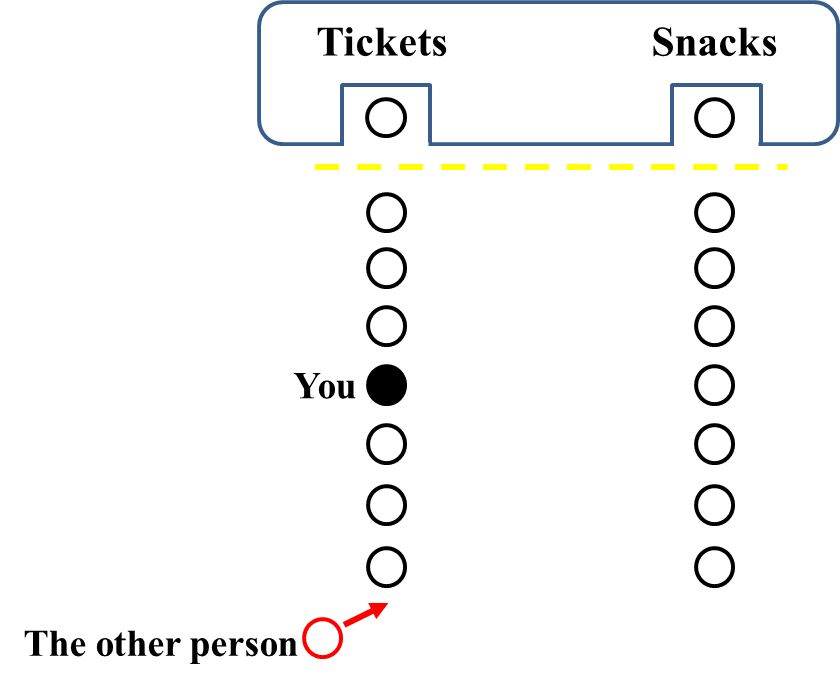


***Control and low self-relevance condition*:** At this moment, you see a person (indicated by the red circle) lining up at the end of the queue for snacks (see below).


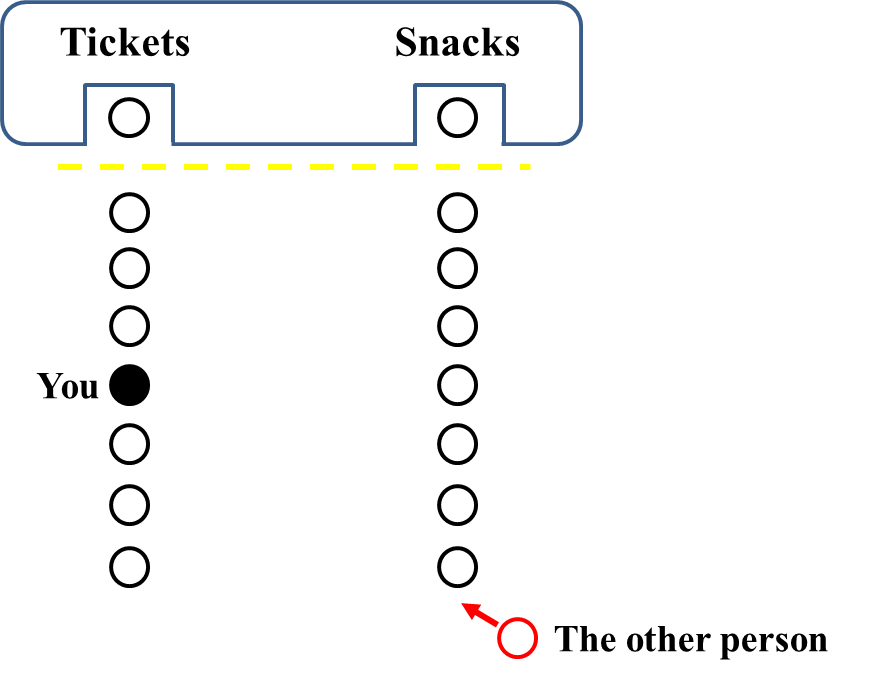


Please imagine this situation for 2 minutes. Then an arrow button will appear and you can click it and go to the next page.

**Dependent Variable**

**Instruction:** We would like to ask you some questions about this person who last joined the line, indicated by the red circle in the scenario. (**The example below is the *Antisocial and high self-relevance condition***)


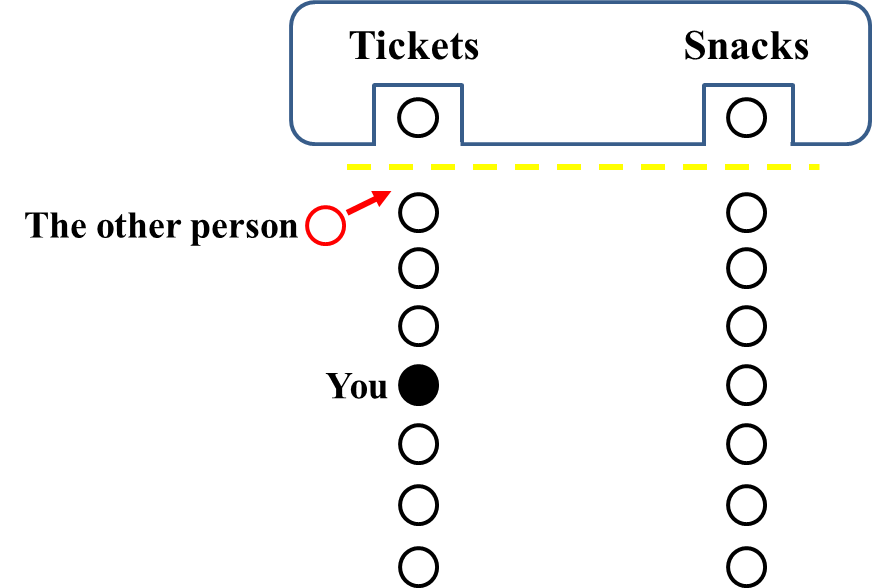


**Moral character evaluation:** Please indicate how much you perceive the following characteristics to be applicable to this person in daily life by choosing from 1 (Not at all) to 7 (Very much).

**The whole scale is available here**: <https://psycnet.apa.org/record/2007-11111-006>

**Mediating variable**

**Recognized antisocial behavior:** Please indicate the degree to which you agree with the following statements about your opinions on this person (the red circle) by choosing from 1 (Strongly disagree) to 7 (Strongly agree). (**Below example is the *Antisocial and high self-relevance condition*)**


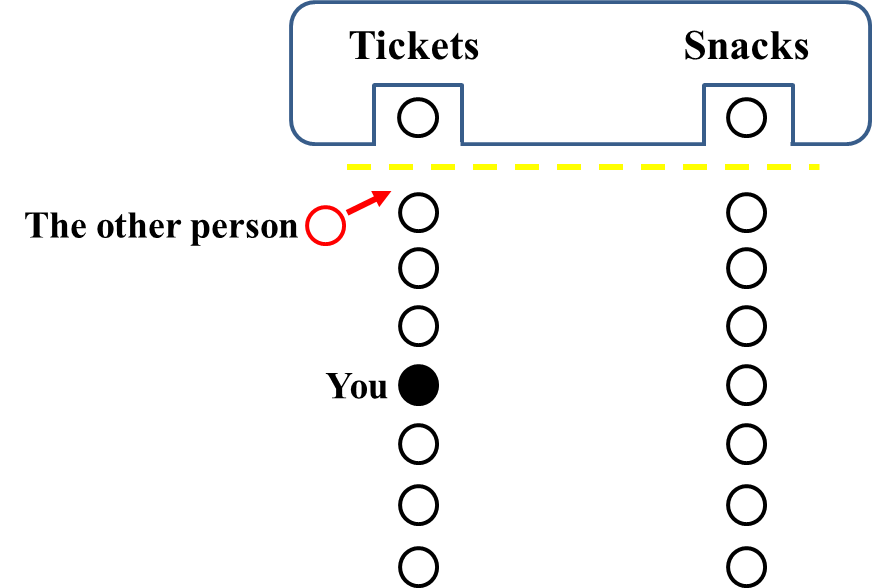


1= Strongly disagree, 2= Disagree, 3= Disagree a little,

4= Neither agree nor disagree

5= Agree a little, 6= Agree, 7= Strongly agree

______1. I think this person behaved inappropriately.

______2. I think this person broke social rules.

______3. I think this person complied with social rules.

**Study 2**

**Independent Variables**

**Narcissism**: The Narcissistic Personality Inventory scale (see Study 1).

**Manipulation of actor’s behavior (control behavior vs. prosocial behavior)**: Now we will provide you with a description of a hypothetical situation. As you are reading this description, try to imagine that you are actually in that situation. Visualize what this situation would look like…

Imagine that you have waited long and eagerly for a popular movie to come out in cinemas. You decide to watch the movie on its release day. After buying a ticket for the movie you line up at the end of the line for snacks. At this moment you notice that there is an electronic screen showing that there are only 8 movie tickets left. Then you overhear a person (person A, indicated by the green circle) beside you in the ticket line talking to another person in front of them saying that they are so lucky to get the last ticket after only a 5 minute walk from home to the movie theater. Shortly thereafter another person (person B, indicated by the blue circle) arrives, looks at the electronic screen, and notices that they are too late for the last ticket. Person B sighs sadly and says aloud that they just took a bus for 1 hour to get here to see this movie and found no ticket left. Just like you, person A can also hear person B.

*
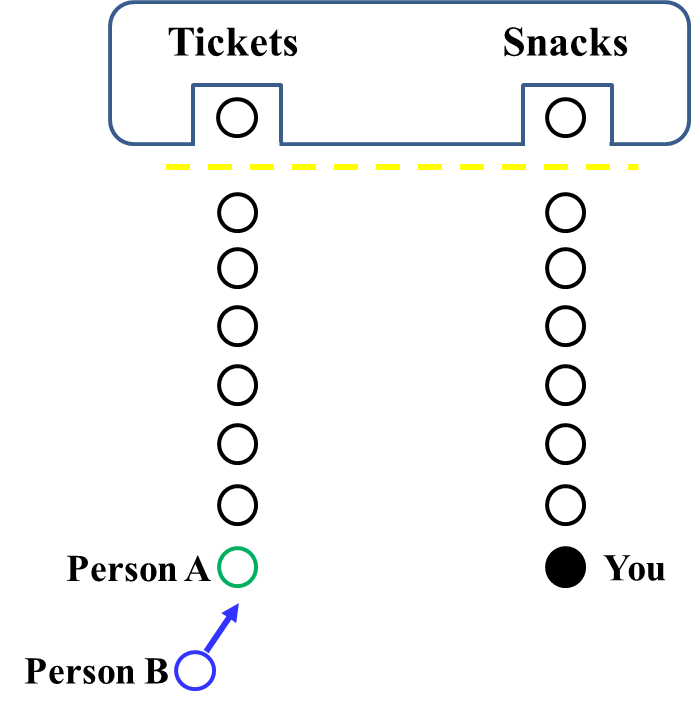
*

***Prosocial condition*:** Then person A steps out of the line and offers their place in the line to person B saying that they themselves can come by tomorrow since they live very close.

***Control condition*:** Then person A turns back and continues to talk with the person in front of them.

Please imagine yourself in this situation for 2 minutes. After these 2 minutes, an arrow button will appear, which you can click to go to the next page.

**Dependent Variable**

**Instruction:** We would like to ask you some questions about person A indicated by the green circle in the scenario.

*
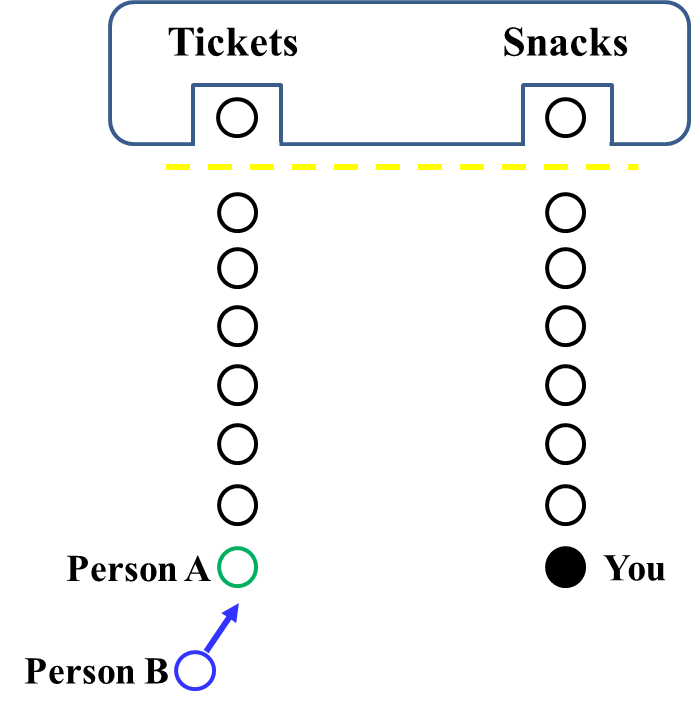
*

**Moral character evaluation:** see Study 1.

**Mediating variable**

**Recognized prosocial behavior:** Please indicate the degree to which you agree with the following statements about your opinion on person A by choosing from 1 (Strongly disagree) to 7 (Strongly agree).

*
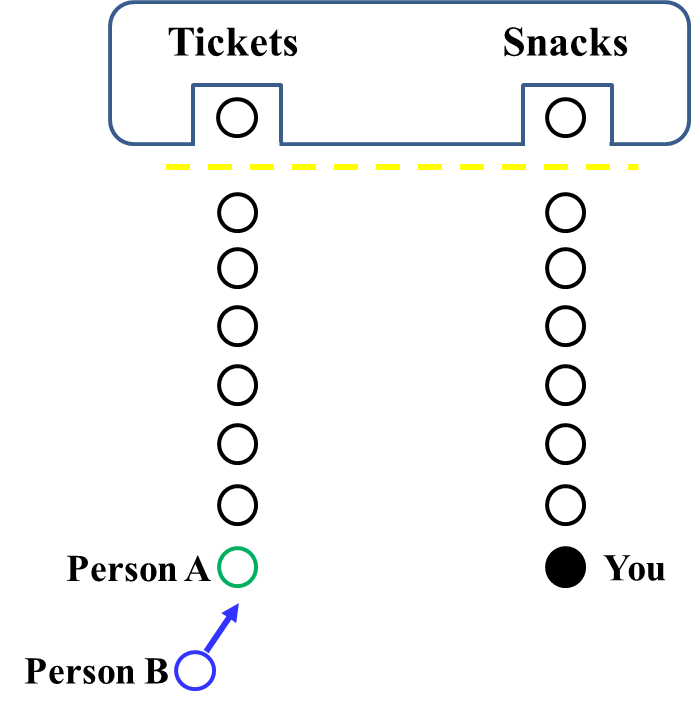
*

1= Strongly disagree, 2= Disagree, 3= Somewhat disagree

4= Neither agree nor disagree

5= Somewhat agree, 6= Agree, 7= Strongly agree

______1. I think person A was kind to person B.

______2. I think person A was helpful to person B.

______3. I think person A was indifferent to person B’s feelings.

______4. I think person A was not concerned about person B’s needs.

**Study 3**

**Independent Variables**

**Manipulation of co-participant’s behavioral tendencies (antisocial vs. prosocial)**: Next, you will play a computer-mediated game with another participant (your co-participant). This participant is currently also taking this online survey somewhere in the USA, and he or she will be matched with you randomly. Before the game starts, we like for you to get to know each other a little bit by exchanging some information about yourselves. To do this, your answers from one of the questionnaires that you just completed will be randomly selected and automatically sent to your co-participant. You will also receive your co-participant's completed answers from one of their randomly selected questionnaires. Please note that no identifying information will be shared with your co-participant. You will remain completely anonymous to each other. When you understand this introduction, please click the arrow button to send out your questionnaire to your co-participant.

**…(Participants waited for 13 seconds to receive their co-participant’s questionnaire that showed either antisocial or prosocial tendencies of the co-participant)**

Below is one of your co-participant's questionnaires that they completed. Please read over their answers carefully one by one, because we will ask you some questions about your co-participant's answers later.

**Antisocial tendencies:**


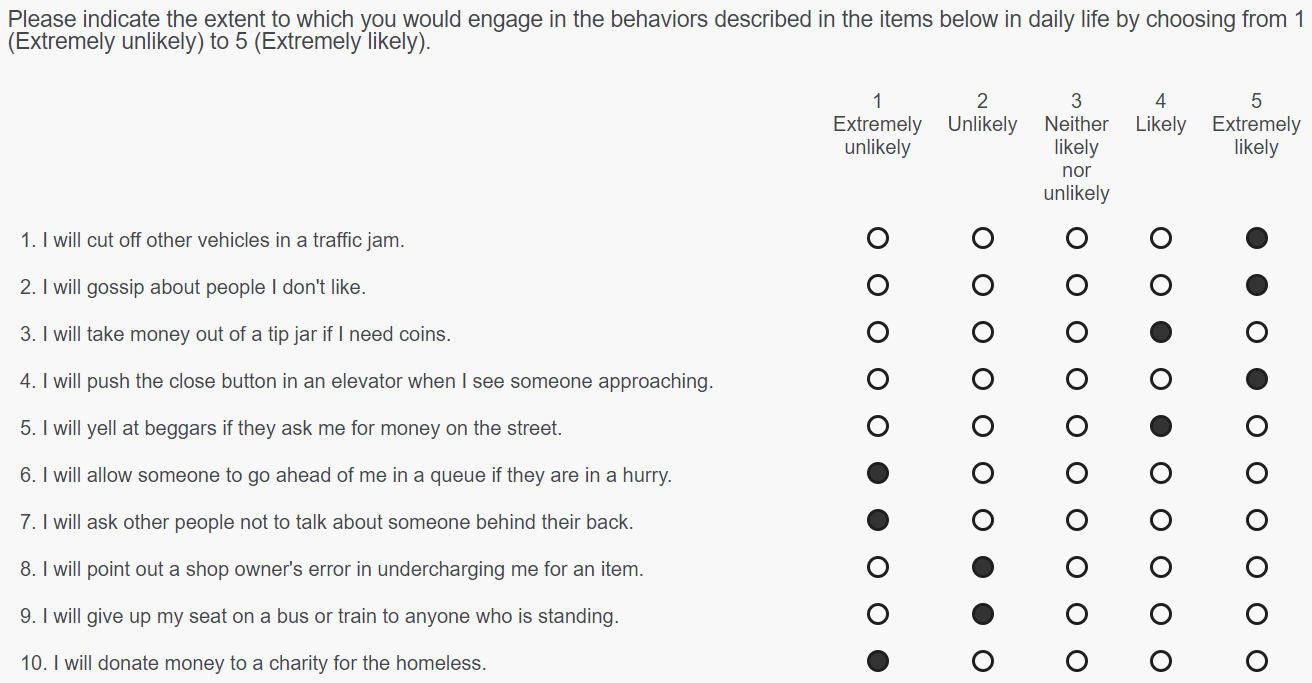


**Prosocial tendencies:**
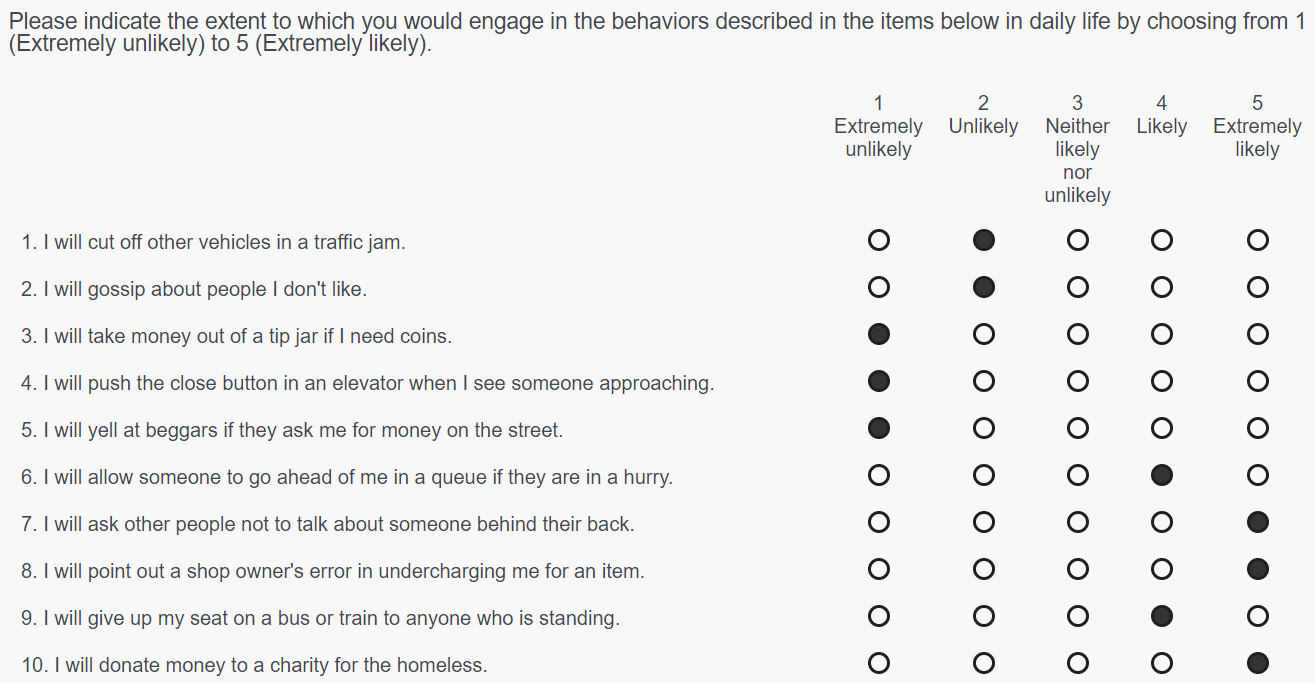


**Narcissism**: The Narcissistic Personality Inventory scale (see Study 1)

**Dependent Variables**

**Moral character evaluation:** See Study 1.

**Reward:** Now it is time for the game! This is a one-shot game involving a sender role and a receiver role. You and your co-participant will be randomly allocated to either the sender or the receiver role to play for lottery tokens. In total, there are 20 lottery tokens. The sender has the option to give any number out of 20 lottery tokens (including 0) to the receiver and take the remaining lottery tokens for themselves. The receiver has to accept the lottery tokens that are offered. After the study, we will put all the lottery tokens from all the participants into a pool. We will then randomly draw 3 tokens to determine the winners of the lottery (each winner gets 10 dollars). The greater the number of lottery tokens you end up with, the greater your chances of getting a prize. Please click the arrow button to continue.

Now, please click the arrow button to start the random role allocation.

**…(Participants waited for 8 seconds to receive the result of role assignment)**

You have been selected to be the SENDER! As you just read, you will now get to decide how many lottery tokens out of 20 to give to your co-participant (the receiver) and how many to keep for yourself:
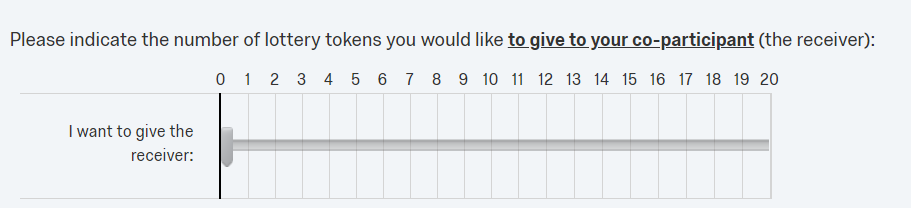


**Punishment:** Imagine that you are in a world where sticking pins into a doll that represents someone will really make that person uncomfortable.


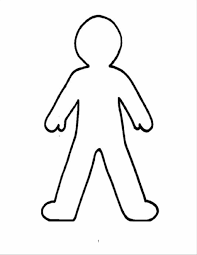


Now please imagine that the doll above represents your co-participant and indicate in the box below how many pins (ranging from 0 to 51) you would like to stick into this doll representing your co-participant.


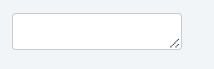


**Mediating variable**

**Recognized social behavior:** Please indicate the degree to which you agree with the following statements about your opinions on what your co-participant is like based on their answers to the questionnaire by choosing from 1 (Strongly disagree) to 7 (Strongly agree).

1= Strongly disagree, 2= Disagree, 3= Somewhat disagree

4= Neither agree nor disagree

5= Somewhat agree, 6= Agree, 7= Strongly agree

______1. I think my co-participant is helpful to others.

______2. I think my co-participant is indifferent to others.

______3. I think my co-participant likes complying with social rules.

______4. I think my co-participant is not concerned about others' needs.

______5. I think my co-participant is considerate to others.

______6. I think my co-participant likes violating rules.

______7. I think my co-participant is kind to others.

______8. I think my co-participant is aggressive towards others.

**Study 4**

**Study 4 was a replication of Study 3, therefore the manipulation of the independent variable and measurements of other variables were almost the same in these two studies, except that in Study 4 we added a monetary incentive in the manipulation instructions and selected four out of eight items from Study 3 to measure the mediator.**

**Independent Variables**

**Manipulation of co-participant’s behavioral tendencies (antisocial vs. prosocial)**: See Study 3.

**…(Participants waited for 13 seconds to receive their co-participant’s questionnaire that showed either antisocial or prosocial tendencies of the co-participant)**

Below is one of your co-participant's questionnaires that they completed. Please read over their answers carefully one by one, because we will ask you some questions about your co-participant based on their answers. This short quiz will appear towards the end of the study and will be clearly announced before it starts. The top 10 performing participants will receive a bonus of $5.

**Antisocial tendencies:** See Study 3

**Prosocial tendencies:** See Study 3

**Narcissism**: The Narcissistic Personality Inventory scale (see Study 1)

**Dependent Variables**

**Moral character evaluation:** See Study 1.

**Reward:** See Study 3

**Punishment:** See Study 3

**Mediating variable**

**Recognized social behavior:** Please indicate the degree to which you agree with the following statements about your opinions on what your co-participant is like based on their answers to the questionnaire by choosing from 1 (Strongly disagree) to 7 (Strongly agree).

1= Strongly disagree, 2= Disagree, 3= Somewhat disagree

4= Neither agree nor disagree

5= Somewhat agree, 6= Agree, 7= Strongly agree

______1. I think my co-participant is helpful to others.

______2. I think my co-participant is indifferent to others.

______3. I think my co-participant likes complying with social rules.

______4. I think my co-participant likes violating rules.
