# Supplementary material for: How Narcissism Shapes Responses to Antisocial and Prosocial Behavior: Hypo-Responsiveness or Hyper-Responsiveness?
Source: Pers Soc Psychol Bull. 2021 Apr 15;48(3):363–81. doi: 10.1177/01461672211007293 (PMC8855397; doi:10.1177/01461672211007293)
Supplement: sj-docx-2-psp-10.1177_01461672211007293 – Supplemental material for How Narcissism Shapes Responses to Antisocial and Prosocial Behavior: Hypo-Responsiveness or Hyper-Responsiveness? [file sj-docx-2-psp-10.1177_01461672211007293.docx]

**Supplemental Materials** (**Results)**

These supplemental materials include the following results:

[1. Results Pertaining to Sociable and Competent Character Evaluations 1](#_Toc63105845)

[2. Effects of Subscales of the NPI (Leadership/Authority, Grandiose Exhibitionism, Entitlement/Exploitativeness) on Participants’ Responsiveness 9](#_Toc63105846)

[3. Effects of the NARQ (Admiration, Rivalry) on Participants’ Responsiveness 27](#_Toc63105847)

[4. Results When Controlling for Participants’ Big Five Personalities 34](#_Toc63105848)

[5. Additional Simple Slopes: Interpretation of Interaction Effects on Responsiveness by Looking at the Effect of Narcissism Within Each of the Conditions 41](#_Toc63105849)

[6. Results After Removing Participants Who Failed on the Manipulation Check 44](#_Toc63105850)

[7. Participants’ Difference in Perceived Similarity with Others 47](#_Toc63105851)

[8. Effects of Self-reported Anti/prosociality of Participants in Explaining Narcissists’ Responsiveness 51](#_Toc63105852)

# Results Pertaining to Sociable and Competent Character Evaluations

The sociable and competent character evaluation measurements were reliable in each study: α = .96, α =.92, in Study 1; α = .94, α =.88, in Study 2; α = .97, α =.95, in Study 3; α = .93, α = .84, in Study 4.

## Study 1

### Sociable Character Evaluation

We used Model 3 in PROCESS to test the effects of actor’s behavior, narcissism, and self-relevance on sociable character evaluation. The results yielded a significant main effect of actor’s behavior, *B* = -2.35, *t*(436) = -21.58, *p* ˂ .001, *r* = .72, 95%CI [-2.56, -2.13], specifically, participants in the antisocial condition (*M* = 2.24, *SD* = 1.27) rated the actor to be less sociable than those in the control condition (*M* = 4.58, *SD* = 1.05). There was no significant main effect of narcissism, *B* = 0.36, *t*(436) = 1.63, *p* = .103, *r* = .08, 95%CI [-0.07, 0.79], nor of self-relevance, *B* = 0.12, *t*(436) = 1.14, *p* = .255, *r* = .05, 95%CI [-0.09, 0.34]. The three-way interaction, *B* = -0.26, *t*(436) = -0.30, *p* = .768, *r* = .01, 95%CI [-1.98, 1.46], and the two-way interactions between self-relevance and actor’s behavior, *B* = 0.36, *t*(436) = 1.66, *p* = .098, *r* = .08, 95%CI [-0.07, 0.79], and between self-relevance and narcissism, *B* = 0.02, *t*(436) = 0.06, *p* = .956, *r* = .003, 95%CI [-0.84, 0.89], were not significant. However, the two-way interaction between actor’s behavior and narcissism was significant, *B* = 2.10, *t*(436) = 4.77, *p* ˂ .001, *r* = .22, 95%CI [1.23, 2.96]. Results of simple slope analysis showed that both low, *B* = -2.94, *t*(440) = -18.40, *p* ˂ .001, *r* = .66, 95%CI [-3.26, -2.63], and high narcissists, *B* = -1.74, *t*(440) = -10.73, *p* ˂ .001, *r* = .46, 95%CI [-2.06, -1.42], rated the actor in the control condition as more sociable than the one in the antisocial condition, but the difference was smaller for high narcissists (see Figure S1A).

### Competent Character Evaluation

We used Model 3 in PROCESS to examine the effects of actor’s behavior, narcissism and self-relevance on competent character evaluation. The results revealed a significant main effect of actor’s behavior, *B* = -0.83, *t*(436) = -6.91, *p* ˂ .001, *r* = .31, 95%CI [-1.06, -0.59], with participants in the antisocial condition (*M* = 3.46, *SD* = 1.47) rating the actor to be less competent than those in the control condition (*M* = 4.28, *SD* = 1.04). Narcissism was found to be positively related to competent character evaluation, *B* = 0.53, *t*(436) = 2.18, *p* = .030, *r* = .10, 95%CI [0.05, 1.00], while there was no significant main effect of self-relevance, *B* = 0.13, *t*(436) = 1.12, *p* = .263, *r* = .05, 95%CI [-0.10, 0.37]. The three-way interaction, *B* = -0.52, *t*(436) = -0.54, *p* = .590, *r* = .03, 95%CI [-2.42, 1.38], and the two-way interaction between self-relevance and narcissism, *B* = -0.19, *t*(436) = -0.39, *p* = .697, *r* = .02, 95%CI [-1.14, 0.76], were not significant. However the two-way interaction between actor’s behavior and narcissism was significant, *B* = 1.50, *t*(436) = 3.10, *p* =.002, *r* = .14, 95%CI [0.55, 2.45]. The results of simple slopes analysis showed that low narcissists rated the actor in the antisocial condition as less competent than the one in the control condition, *B* = -1.28, *t*(440) = -7.24, *p* ˂ .001, *r* = .33, 95%CI [-1.63, -0.93], while the difference between the two conditions was smaller for high narcissists, *B* = -0.38, *t*(440) = -2.15, *p* = .032, *r* = .10, 95%CI [-0.74, -0.03] (see Figure S2A). The two-way interaction between actor’s behavior and self-relevance was also significant, *B* = 0.51, *t*(436) = 2.14, *p* =.033, *r* = .10, 95%CI [0.04, 0.99]. The results of simple slope showed that participants in both low, *B* = -1.12, *t*(440) = -6.66, *p* ˂ .001, *r* = .30, 95%CI [-1.45, -0.79], and high self-relevance condition, *B* = -0.51, *t*(440) = -2.98, *p* = .003, *r* = .14, 95%CI [-0.85, -0.17], indicated that the actor in the antisocial condition to be less competent than the one in the control condition, but the difference between the two conditions was smaller for participants in the high self-relevant condition (see Figure S2B).

## Study 2

### Sociable Character Evaluation

We used Model 1 in PROCESS to examine the effects of actor’s behavior and narcissism on the evaluation of the actor’s sociable character. The results revealed a significant main effect of actor’s behavior, *B* =2.13, *t*(245) = 12.47, *p* ˂ .001, *r* = .62, 95%CI [1.79, 2.47], with participants in the prosocial condition (*M* = 5.99, *SD* = 1.26) evaluating the actor as more sociable than in the control condition (*M* = 3.86, *SD* = 1.53). Narcissism did not significantly predict sociable character evaluation, *B* = 0.57, *t*(245) = 1.96, *p* = .051, *r* = .12, 95%CI [-0.003, 1.15]. The interaction effect was significant, *B* = -2.52, *t*(245) = -4.31, *p* ˂ .001, *r* = .27, 95%CI [-3.67, -1.37], such that low narcissistic participants rated the actor in the prosocial condition as more sociable than the one in the control condition, *B* = 3.01, *t*(245) = 11.29, *p* ˂ .001, *r* = .58, 95%CI [2.49, 3.54], but the difference between the two conditions was smaller for participants with high narcissism, *B* = 1.25, *t*(245) = 4.68, *p* ˂ .001, *r* = .29, 95%CI [0.72, 1.77] (see Figure S1B).

### Competent Character Evaluation

We employed Model 1 in PROCESS to test the effects of actor’s behavior and narcissism on competent character evaluation of the actor. The results showed a significant main effect of actor’s behavior, *B* = 0.78, *t*(245) = 5.04, *p* ˂.001, *r* = .31, 95%CI [0.48, 1.09], with participants in the prosocial condition (*M* = 5.17, *SD* = 1.15) evaluating the actor as more competent than those in control condition (*M* = 4.39, *SD* = 1.31). Narcissism significantly predicted competent character evaluation, *B* = 0.76, *t*(245) = 2.87, *p* = .004, *r* = .18, 95%CI [0.24, 1.28]. However, the interaction was not significant, *B* = -0.31, *t*(245) = -0.59, *p* = .553, *r* = .04, 95%CI [-1.36, 0.73].

## Study 3

### Sociable Character Evaluation

We examined the effects of co-participant’s behavioral tendencies and narcissism on sociable character evaluation, using Model 1 in PROCESS. The results yielded a significant main effect of the behavioral tendencies, *B* = 3.50, *t*(245) = 19.16, *p* ˂ .001, *r* = .77, 95%CI [3.14, 3.86], such that the co-participant in the prosocial condition was rated to be more sociable (*M* = 5.91, *SD* = 1.21) than that in the antisocial condition (*M* = 2.38, *SD* = 1.82). Narcissism significantly predicted sociable character evaluation, *B* = 1.20, *t*(245) = 3.64, *p* ˂ .001, *r* = .23, 95%CI [0.55, 1.85]. The interaction effect was also significant, *B* = -3.62, *t*(245) = -5.49, *p* ˂ .001, *r* = .33, 95%CI [-4.92, -2.32]. Simple slope results showed that low narcissistic participants evaluated the co-participant in the prosocial condition as more sociable than the one in the antisocial condition, *B* = 4.62, *t*(245) = 116.87, *p* ˂ .001, *r* = .73, 95%CI [4.08, 5.16], while the difference between the two conditions was smaller for high narcissists, *B* = 2.36, *t*(245) = 8.51, *p* ˂ .001, *r* = .48, 95%CI [1.81, 2.90] (see Figure S1C).

### Competent Character Evaluation

We used Model 1 in PROCESS to test the effects of co-participant’s behavioral tendencies and narcissism on the evaluation of the co-participant’s competent character. The results revealed a significant main effect of the behavioral tendencies, *B* = 1.70, *t*(245) = 10.34, *p* ˂ .001, *r* = .55, 95%CI [1.38, 2.03], such that participants in the prosocial condition (*M* = 5.56, *SD* = 1.06) evaluated the co-participant as more competent than those in the antisocial condition (*M* = 3.84, *SD* = 1.52). The main effect of narcissism was also significant, revealing a positive relationship, *B* = 0.63, *t*(245) = 2.11, *p* = .035, *r* = .13, 95%CI [.04, 1.21]. The interaction effect was also significant, *B* = -1.41, *t*(245) = -2.38, *p* = .018, *r* = .15, 95%CI [-2.59, -0.24]. Simple slope results showed that participants with both low, *B* = 2.14, *t*(245) = 8.67, *p* ˂ .001, *r* = .48, 95%CI [1.65, 2.63], and high narcissism, *B* = 1.26, *t*(245) = 5.03, *p* ˂ .001, *r* = .31, 95%CI [0.76, 1.75], rated the co-participant in the prosocial condition as being more competent than the actor in the antisocial condition, with the difference between the two conditions being smaller for high narcissistic participants (see Figure S2C).

## Study 4

### Sociable Character Evaluation

We examined the effects of co-participant’s behavioral tendencies and narcissism on sociable character evaluation, using Model 1 in PROCESS. The results yielded a significant main effect of the behavioral tendencies, *B* = 1.58, *t*(238) = 8.64, *p* ˂ .001, *r* = .49, 95%CI [1.22, 1.95], such that the co-participant in the prosocial condition was rated to be more sociable (*M* = 5.75, *SD* = 0.99) than that in the antisocial condition (*M* = 4.16, *SD* = 2.07). Narcissism significantly predicted sociable character evaluation, *B* = 1.96, *t*(238) = 6.22, *p* ˂ .001, *r* = .37, 95%CI [1.34, 2.59]. The interaction effect was also significant, *B* = -4.10, *t*(238) = -6.50, *p* ˂ .001, *r* = .39, 95%CI [-5.35, -2.86]. Simple slope results showed that low narcissistic participants evaluated the co-participant in the prosocial condition as more sociable than the one in the antisocial condition, *B* = 2.78, *t*(238) = 10.70, *p* ˂ .001, *r* = .57, 95%CI [2.27, 3.30], while the difference between the two conditions was not significant for high narcissists, *B* = 0.38, *t*(238) = 1.48, *p* = .141, *r* = .10, 95%CI [-0.13, 0.90] (see Figure S1D).

### Competent Character Evaluation

We used Model 1 in PROCESS to test the effects of co-participant’s behavioral tendencies and narcissism on the evaluation of the co-participant’s competent character. The results revealed a significant main effect of the behavioral tendencies, *B* = 0.77, *t*(238) = 5.30, *p* ˂ .001, *r* = .32, 95%CI [0.48, 1.06], such that participants in the prosocial condition (*M* = 5.67, *SD* = 0.81) evaluated the co-participant as more competent than those in the antisocial condition (*M* = 4.90, *SD* = 1.48). The main effect of narcissism was also significant, revealing a positive relationship, *B* = 1.10, *t*(238) = 4.39, *p* ˂ .001, *r* = .27, 95%CI [0.61, 1.59]. The interaction effect was also significant, *B* = -1.82, *t*(238) = -3.63, *p* ˂ .001, *r* = .23, 95%CI [-2.80, -0.83]. Simple slope results showed that participants with low narcissism rated the co-participant in the prosocial condition as more competent than the one in the antisocial condition, *B* = 1.30, *t*(238) = 6.31, *p* ˂ .001, *r* = .38, 95%CI [0.89, 1.71], while the difference was not significant for participants with high narcissism, *B* = 0.24, *t*(238) = 1.16, *p* = .248, *r* = .07, 95%CI [-0.17, 0.65] (see Figure S2D).

## Conclusion

Participants showed similar responding patterns on sociable character evaluation with moral character evaluation in four studies, while competent character evaluation shows a similar pattern except for in Study 2. In addition, in Study 1, participants in the high self-relevance condition seemed to differentiate less between the actor in the control and antisocial conditions on competent character evaluation.

**Figure S1**

*Responsiveness on Sociable Character Evaluation (Studies 1-4)*


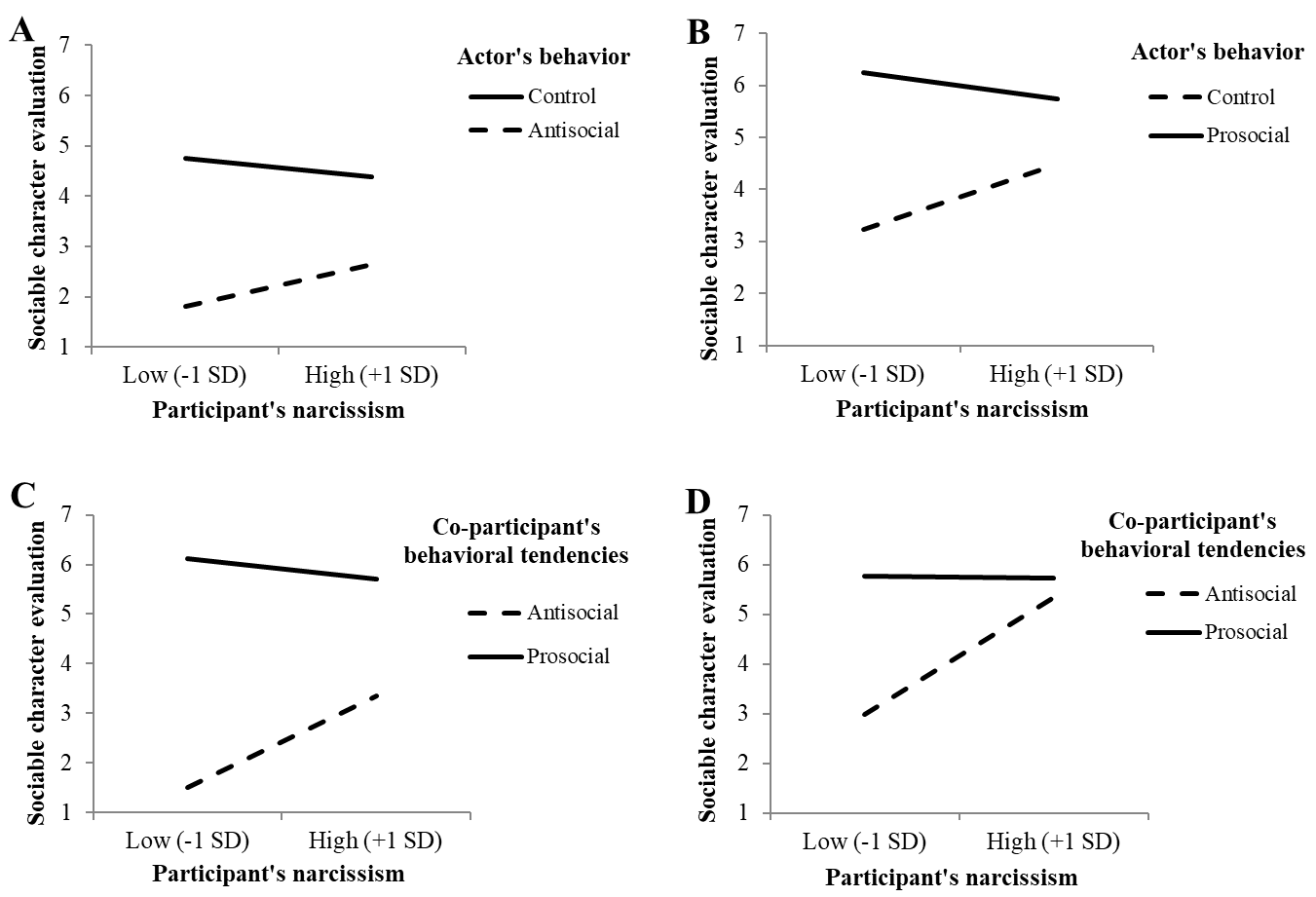


*Note.* Interaction between actor’s behavior and participant’s narcissism on sociable character evaluation of the actor (A, Study 1; B, Study 2); Interaction between co-participant’s behavioral tendencies and participant’s narcissism on sociable character evaluation of the co-participant (C, Study 3; D, Study 4). “High” and “low” narcissism refer to scores on the NPI scale that were one standard deviation above the mean or one standard deviation below the mean, respectively.

**Figure S2**

*Responsiveness on Competent Character Evaluation (Studies 1, 3, 4)*

*
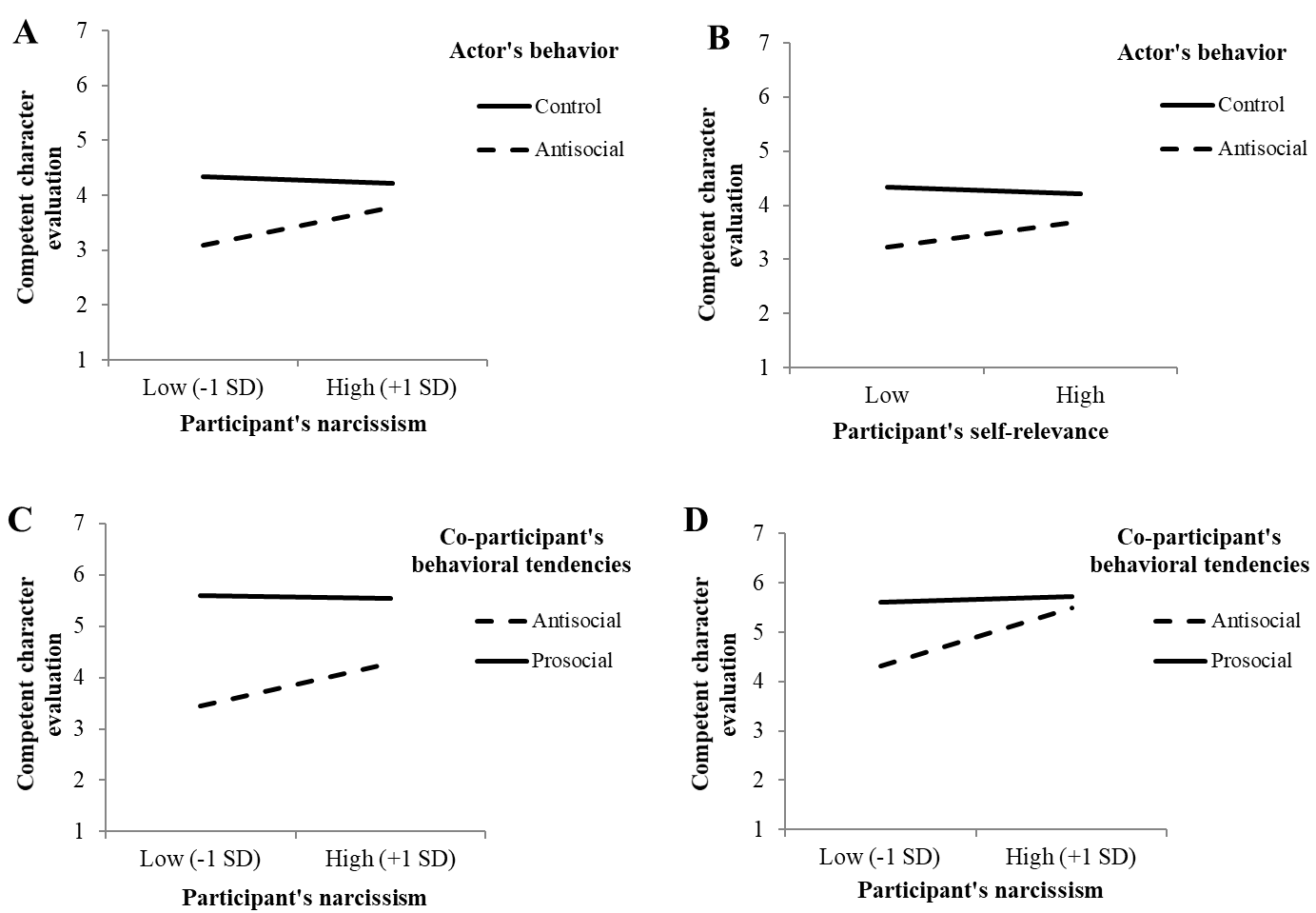
*

*Note.* Interaction between actor’s behavior and participant’s narcissism on competent character evaluation of the actor (A, Study 1); Interaction between actor’s behavior and participant’s self-relevance on competent character evaluation of the actor (B, Study 1); Interaction between co-participant’s behavioral tendencies and participant’s narcissism on competent character evaluation of the co-participant (B, Study 3; C, Study 4). “High” and “low” narcissism refer to scores on the NPI scale that were one standard deviation above the mean or one standard deviation below the mean, respectively.

# Effects of Subscales of the NPI (Leadership/Authority, Grandiose Exhibitionism, Entitlement/Exploitativeness) on Participants’ Responsiveness

The measurements of leadership/authority, grandiose exhibitionism, entitlement/exploitativeness showed the following reliabilities: α = .89, α = .85, α = .58, in Study 1; α = .89, α = .88, α = .70, in Study 2; α = .89, α = .88, α = .69, in Study 3; α = .87, α = .88, α = .70, in Study 4.

## Study 1

### Moral Character Evaluation

Effects of Leadership/Authority on Moral Character Evaluation. We used Model 3 in PROCESS to test the effects of actor’s behavior, leadership/authority, and self-relevance on moral character evaluation. The results yielded a significant main effect of actor’s behavior, *B* = -2.54, *t*(436) = -22.03, *p* ˂ .001, *r* = .73, 95%CI [-2.77, -2.32], specifically, participants in the antisocial condition (*M* = 2.07, *SD* = 1.36) rated the actor to be less moral than those in the control condition (*M* = 4.62, *SD* = 1.08). There was no significant main effect of leadership/authority, *B* = 0.11, *t*(436) = 0.62, *p* = .540, *r* = .03, 95%CI [-0.24, 0.45], nor of self-relevance, *B* = 0.10, *t*(436) = 0.86, *p* = .388, *r* = .04, 95%CI [-0.13, 0.33]. The three-way interaction, *B* = -0.16, *t*(436) = -0.22, *p* = .824, *r* = .01, 95%CI [-1.53, 1.22], and the two-way interactions between self-relevance and actor’s behavior, *B* = 0.22, *t*(436) = 0.95, *p* = .341, *r* = .05, 95%CI [-0.23, 0.67], and between self-relevance and leadership/authority, *B* = -0.09, *t*(436) = -0.26, *p* = .796, *r* = .01, 95%CI [-0.78, 0.60], were not significant. However, the two-way interaction between actor’s behavior and leadership/authority was significant, *B* = 1.32, *t*(436) = 3.79, *p* ˂ .001, *r* = .18, 95%CI [0.64, 2.01]. Results of simple slope analysis showed that both participants with low, *B* = -3.00, *t*(440) = -18.47, *p* ˂ .001, *r* = .66, 95%CI [-3.32, -2.68], and high leadership/authority, *B* = -2.10, *t*(440) = -12.90, *p* ˂ .001, *r* = .52, 95%CI [-2.42, -1.78], rated the actor in the control condition as more moral than the one in the antisocial condition, but the difference was smaller for those with high leadership/authority.

**Effects of Grandiose Exhibitionism on** Moral Character Evaluation**.** We used Model 3 in PROCESS to test the effects of actor’s behavior, grandiose exhibitionism, and self-relevance on moral character evaluation. The results yielded a significant main effect of actor’s behavior, *B* = -2.56, *t*(436) = -22.83, *p* ˂ .001, *r* = .74, 95%CI [-2.78, -2.34], specifically, participants in the antisocial condition (*M* = 2.07, *SD* = 1.36) rated the actor to be less moral than those in the control condition (*M* = 4.62, *SD* = 1.08). The main effect of grandiose exhibitionism was also significant, *B* = 0.64, *t*(436) = 3.05, *p* = .002, *r* = .14, 95%CI [0.23, 1.06], while the main effect of self-relevance was not significant, *B* = 0.07, *t*(436) = 0.65, *p* = .535, *r* = .03, 95%CI [-0.15, 0.29]. The three-way interaction, *B* = 1.04, *t*(436) = 1.23, *p* = .218, *r* = .06, 95%CI [-0.62, 2.69], and the two-way interactions between self-relevance and actor’s behavior, *B* = 0.14, *t*(436) = 0.62, *p* = .533, *r* = .03, 95%CI [-0.30, 0.58], and between self-relevance and grandiose exhibitionism, *B* = 0.32, *t*(436) = 0.76, *p* = .446, *r* = .04, 95%CI [-0.51, 1.15], were not significant. However, the two-way interaction between actor’s behavior and grandiose exhibitionism was significant, *B* = 2.36, *t*(436) = 5.60, *p* ˂ .001, *r* = .26, 95%CI [1.53, 3.19]. Results of simple slope analysis showed that both participants with low, *B* = -3.17, *t*(440) = -20.51, *p* ˂ .001, *r* = .70, 95%CI [-3.47, -2.86], and high grandiose exhibitionism, *B* = -1.89, *t*(440) = -11.99, *p* ˂ .001, *r* = .48, 95%CI [-2.20, -1.58], rated the actor in the control condition as more moral than the one in the antisocial condition, but the difference was smaller for those with high grandiose exhibitionism.

**Effects of Entitlement/Exploitativeness on** Moral Character Evaluation**.** We used Model 3 in PROCESS to test the effects of actor’s behavior, entitlement/exploitativeness, and self-relevance on moral character evaluation. The results yielded a significant main effect of actor’s behavior, *B* = -2.55, *t*(436) = -22.36, *p* ˂ .001, *r* = .73, 95%CI [-2.78, -2.33], specifically, participants in the antisocial condition (*M* = 2.07, *SD* = 1.36) rated the actor to be less moral than those in the control condition (*M* = 4.62, *SD* = 1.08). There was no significant main effect of entitlement/exploitativeness, *B* = 0.05, *t*(436) = 0.27, *p* = .783, *r* = .01, 95%CI [-0.32, 0.42], nor of self-relevance, *B* = 0.10, *t*(436) = 0.86, *p* = .391, *r* = .04, 95%CI [-0.13, 0.32]. The three-way interaction, *B* = 0.92, *t*(436) = 1.23, *p* = .221, *r* = .06, 95%CI [-0.55, 2.39], and the two-way interactions between self-relevance and actor’s behavior, *B* = 0.18, *t*(436) = 0.82, *p* = .423, *r* = .04, 95%CI [-0.27, 0.63], and between self-relevance and entitlement/exploitativeness, *B* = 0.19, *t*(436) = 0.50, *p* = .629, *r* = .02, 95%CI [-0.92, 0.55], were not significant. However, the two-way interaction between actor’s behavior and entitlement/exploitativeness was significant, *B* = 1.76, *t*(436) = 4.72, *p* ˂ .001, *r* = .22, 95%CI [1.03, 2.50]. Results of simple slope analysis showed that both participants with low, *B* = -3.10, *t*(440) = -19.29, *p* ˂ .001, *r* = .68, 95%CI [-3.41, -2.78], and high entitlement/exploitativeness, *B* = -1.99, *t*(440) = -12.36, *p* ˂ .001, *r* = .51, 95%CI [-2.31, -1.67], rated the actor in the control condition as more moral than the one in the antisocial condition, but the difference was smaller for those with high entitlement/exploitativeness.

## Study 2

### Moral Character Evaluation

Effects of Leadership/Authority on Moral Character Evaluation. We used Model 1 in PROCESS to test the effects of actor’s behavior and leadership/authority on moral character evaluation. The results yielded a significant main effect of actor’s behavior, *B* = 1.47, *t*(245) = 8.81, *p* ˂ .001, *r* = .49, 95%CI [1.14, 1.80], specifically, participants in the prosocial condition (*M* = 5.68, *SD* = 1.20) rated the actor to be more moral than those in the control condition (*M* = 4.21, *SD* = 1.46). There was no significant main effect of leadership/authority, *B* = 0.27, *t*(245) = 1.13, *p* = .260, *r* = .07, 95%CI [-0.20, 0.75]. However, the two-way interaction between actor’s behavior and leadership/authority was significant, *B* = -1.28, *t*(245) = -2.64, *p* = .009, *r* = .17, 95%CI [-2.24, -0.32]. Results of simple slope analysis showed that both participants with low, *B* = 1.92, *t*(245) = 8.09, *p* ˂ .001, *r* = .46, 95%CI [1.45, 2.38], and high leadership/authority, *B* = 1.03, *t*(245) = 4.35, *p* ˂ .001, *r* = .27, 95%CI [0.57, 1.50], rated the actor in the prosocial condition as more moral than the one in the control condition, but the difference was smaller for those with high leadership/authority.

**Effects of Grandiose Exhibitionism on** Moral Character Evaluation**.** We used Model 1 in PROCESS to test the effects of actor’s behavior and grandiose exhibitionism on moral character evaluation. The results yielded a significant main effect of actor’s behavior, *B* = 1.47, *t*(245) = 8.82, *p* ˂ .001, *r* = .49, 95%CI [1.14, 1.80], specifically, participants in the prosocial condition (*M* = 5.68, *SD* = 1.20) rated the actor to be more moral than those in the control condition (*M* = 4.21, *SD* = 1.46). There was a marginally significant main effect of grandiose exhibitionism, *B* = 0.50, *t*(245) = 1.97, *p* = .050, *r* = .12, 95%CI [0, 1.01]. However, the two-way interaction between actor’s behavior and grandiose exhibitionism was significant, *B* = -1.31, *t*(245) = -2.55, *p* = .012, *r* = .16, 95%CI [-2.31, -0.30]. Results of simple slope analysis showed that both participants with low, *B* = 1.89, *t*(245) = 8.01, *p* ˂ .001, *r* = .46, 95%CI [1.43, 2.36], and high grandiose exhibitionism, *B* = 1.04, *t*(245) = 4.41, *p* ˂ .001, *r* = .27, 95%CI [0.58, 1.51], rated the actor in the prosocial condition as more moral than the one in the control condition, but the difference was smaller for those with high grandiose exhibitionism

**Effects of Entitlement/Exploitativeness on** Moral Character Evaluation**.** We used Model 1 in PROCESS to test the effects of actor’s behavior and entitlement/exploitativeness on moral character evaluation. The results yielded a significant main effect of actor’s behavior, *B* = 1.47, *t*(245) = 8.85, *p* ˂ .001, *r* = .49, 95%CI [1.15, 1.80], specifically, participants in the prosocial condition (*M* = 5.68, *SD* = 1.20) rated the actor to be more moral than those in the control condition (*M* = 4.21, *SD* = 1.46). There was no significant main effect of entitlement/exploitativeness, *B* = 0.14, *t*(245) = 0.58, *p* = .560, *r* = .04, 95%CI [-0.32, 0.59]. However, the two-way interaction between actor’s behavior and entitlement/exploitativeness was significant, *B* = -1.51, *t*(245) = -3.24, *p* = .001, *r* = .20, 95%CI [-2.42, -0.59]. Results of simple slope analysis showed that both participants with low, *B* = 2.02, *t*(245) = 8.55, *p* ˂ .001, *r* = .48, 95%CI [1.55, 2.48], and high entitlement/exploitativeness, *B* = 0.93, *t*(245) = 3.94, *p* ˂ .001, *r* = .24, 95%CI [0.46, 1.10], rated the actor in the prosocial condition as more moral than the one in the control condition, but the difference was smaller for those with high entitlement/exploitativeness.

## Study 3

### Moral Character Evaluation

Effects of Leadership/Authority on Moral Character Evaluation. We used Model 1 in PROCESS to test the effects the co-participant’s behavioral tendencies and leadership/authority on moral character evaluation. The results yielded a significant main effect of the behavioral tendencies, *B* = 3.06, *t*(245) = 15.98, *p* ˂ .001, *r* = .71, 95%CI [2.69, 3.44], specifically, participants in the prosocial condition (*M* = 5.93, *SD* = 1.09) rated their co-participant to be more moral than those in the antisocial condition (*M* = 2.85, *SD* = 1.68). There was no significant main effect of leadership/authority, *B* = 0.53, *t*(245) = 1.90, *p* = .095, *r* = .12, 95%CI [-0.02, 1.08]. However, the two-way interaction between behavioral tendencies and leadership/authority was significant, *B* = -1.42, *t*(245) = -2.53, *p* = .012, *r* = .16, 95%CI [-2.53, -0.32]. Results of simple slope analysis showed that both participants with low, *B* = 3.55, *t*(245) = 13.07, *p* ˂ .001, *r* = .64, 95%CI [3.01, 4.08], and high leadership/authority, *B* = 2.58, *t*(245) = 9.50, *p* ˂ .001, *r* = .52, 95%CI [2.04, 3.11], rated their co-participant in the prosocial condition as more moral than the one in the antisocial condition, but the difference was smaller for those with high leadership/authority.

**Effects of Grandiose Exhibitionism on** Moral Character Evaluation**.** We used Model 1 in PROCESS to test the effects the co-participant’s behavioral tendencies and grandiose exhibitionism on moral character evaluation. The results yielded a significant main effect of the behavioral tendencies, *B* = 3.01, *t*(245) = 16.95, *p* ˂ .001, *r* = .73, 95%CI [2.66, 3.36], specifically, participants in the prosocial condition (*M* = 5.93, *SD* = 1.09) rated their co-participant to be more moral than those in the antisocial condition (*M* = 2.85, *SD* = 1.68). There main effect of grandiose exhibitionism was significant, *B* = 1.36, *t*(245) = 4.61, *p* ˂ .001, *r* = .28, 95%CI [0.78, 1.94]. The two-way interaction between behavioral tendencies and grandiose exhibitionism was also significant, *B* = -3.58, *t*(245) = -6.07, *p* ˂ .001, *r* = .36, 95%CI [-4.74, -2.42]. Results of simple slope analysis showed that both participants with low, *B* = 4.10, *t*(245) = 16.29, *p* ˂ .001, *r* = .72, 95%CI [3.60, 4.59], and high grandiose exhibitionism, *B* = 1.93, *t*(245) = 7.66, *p* ˂ .001, *r* = .44, 95%CI [1.43, 2.43], rated their co-participant in the prosocial condition as more moral than the one in the antisocial condition, but the difference was smaller for those with high grandiose exhibitionism.

**Effects of Entitlement/Exploitativeness on** Moral Character Evaluation**.** We used Model 1 in PROCESS to test the effects the co-participant’s behavioral tendencies and entitlement/exploitativeness on moral character evaluation. The results yielded a significant main effect of the behavioral tendencies, *B* = 3.07, *t*(245) = 16.14, *p* ˂ .001, *r* = .72, 95%CI [2.70, 3.45], specifically, participants in the prosocial condition (*M* = 5.93, *SD* = 1.09) rated their co-participant to be more moral than those in the antisocial condition (*M* = 2.85, *SD* = 1.68). There was no significant main effect of entitlement/exploitativeness, *B* = 0.40, *t*(245) = 1.46, *p* = .146, *r* = .09, 95%CI [-0.14, 0.94]. However, the two-way interaction between behavioral tendencies and entitlement/exploitativeness was significant, *B* = -1.81, *t*(245) = -3.32, *p* = .001, *r* = .21, 95%CI [-2.89, -0.74]. Results of simple slope analysis showed that both participants with low, *B* = 3.71, *t*(245) = 13.75, *p* ˂ .001, *r* = .66, 95%CI [3.18, 4.24], and high entitlement/exploitativeness, *B* = 2.44, *t*(245) = 9.05, *p* ˂ .001, *r* = .50, 95%CI [1.91, 2.97], rated their co-participant in the prosocial condition as more moral than the one in the antisocial condition, but the difference was smaller for those with high entitlement/exploitativeness.

### Reward

**Effects of** Leadership/Authority **on Reward**. The results of Model 1 (PROCESS) revealed a significant main effect of behavioral tendencies on reward, such that participants in the prosocial condition (68.60%, *N* = 83 out of 121) were 4.61 times more likely to offer a high (vs. low) reward to the co-participant than those in the antisocial condition (32.81%, *N* = 42 out of 128), *B* = 1.53, *p* < .001, 95%CI [0.99, 2.09]. Leadership/authority was found to positively predict reward, *B* = 1.10, *p* = .008, *OR* (Odds Ratio) = 3.01, 95%CI [0.29, 1.90]. However, the interaction was not significant, *B* = -1.23, *p* = .133, *OR* = 0.29, 95%CI [-2.84, 0.37].

**Effects of Grandiose Exhibitionism on Reward.** The results of Model 1 (PROCESS) revealed a significant main effect of behavioral tendencies on reward, such that participants in the prosocial condition (68.60%, *N* = 83 out of 121) were 4.58 times more likely to offer a high (vs. low) reward to the co-participant than those in the antisocial condition (32.81%, *N* = 42 out of 128), *B* = 1.51, *p* < .001, 95%CI [0.96, 2.07]. Grandiose exhibitionism was found to positively predict reward, *B* = 1.88, *p* < .001, *OR* = 6.45, 95%CI [0.94, 2.83]. However, the interaction was not significant, *B* = -1.70, *p* = .077, *OR* = 0.18, 95%CI [-3.58, 0.18].

**Effects of Entitlement/Exploitativeness on Reward.** The results of Model 1 (PROCESS) revealed a significant main effect of behavioral tendencies on reward, such that participants in the prosocial condition (68.60%, *N* = 83 out of 121) were 4.54 times more likely to offer a high (vs. low) reward to the co-participant than those in the antisocial condition (32.81%, *N* = 42 out of 128), *B* = 1.54, *p* < .001, 95%CI [1.00, 2.08]. The main effect of entitlement/exploitativeness was not significant, *B* = 0.52, *p* = .194, *OR* = 1.69, 95%CI [-0.26, 1.28]. However, the interaction was significant, *B* = -1.65, *p* = .037, *OR* = 0.19, 95%CI [-3.19, -0.10]. Results of simple slope analysis showed that participants with low entitlement/exploitativeness were 8.28 times more likely to give high reward to their co-participants in the prosocial condition than the one in the antisocial condition, *B* = 2.11, *p* ˂ .001, 95%CI [1.31, 2.91], while those with high entitlement/exploitativeness were only 2.62 times more likely to give high reward to their co-participants in the prosocial condition than the one in the antisocial condition, *B* = 0.96, *p* =.010, 95%CI [0.23, 1.69].

**Mediated Moderation Models.** We examined the indirect effects on reward via recognized anti/prosociality using Model 8 in PROCESS with leadership/authority, grandiose exhibitionism, entitlement/exploitativeness as the moderators. The first step suggested that the interactions between behavioral tendencies and leadership/authority (*B* = -2.46, *t*(245) = -5.59, *p* <.001, *r* = .34, 95%CI [-3.31, -1.61]), and between behavioral tendencies and grandiose exhibitionism (*B* = -4.56, *t*(245) = -10.46, *p* <.001, *r* = .56, 95%CI [-5.42, -3.70]), and behavioral tendencies and entitlement/exploitativeness (*B* = -2.90, *t*(245) = -7.05, *p* <.001, *r* = .41, 95%CI [-3.71, -2.09]) were significant on recognized anti/prosociality. Specifically compared with participants with low leadership/authority (*B* = 3.96, *t*(245) = 18.90, *p* < .001, *r* = .77, 95%CI [2.54, 4.37]), low grandiose exhibitionism (*B* = 4.48, *t*(245) = 24.12, *p* < .001, *r* = .84, 95%CI [2.12, 4.85]), or low entitlement/exploitativeness (*B* = 4.12, *t*(245) = 20.29, *p* < .001, *r* = .79, 95%CI [3.72, 4.52]), those with high leadership/authority (*B* = 2.27, *t*(245) = 10.88, *p* < .001, *r* = .57, 95%CI [1.86, 2.68]), high grandiose exhibitionism (*B* = 1.73, *t*(245) = 9.26, *p* < .001, *r* = .51, 95%CI [1.36, 2.09]), or high entitlement/exploitativeness (*B* = 2.09, *t*(245) = 10.30, *p* < .001, *r* = .55, 95%CI [1.69, 2.49]) displayed a smaller difference in recognized anti/prosociality between the two conditions.

The second step showed that recognized anti/prosociality positively predicted reward with leadership/authority (*B* = 0.40, *p* = .001, 95%CI [0.16, 0.64]), grandiose exhibitionism (*B* = 0.40, *p* = .005, 95%CI [0.12, 0.67]), entitlement/exploitativeness (*B* = 0.35, *p* = .004, 95%CI [0.11, 0.29]) when controlling for behavioral tendencies, leadership/authority, or grandiose exhibitionism, or entitlement/exploitativeness, and their interaction. The third step, examining the indirect effect of behavioral tendencies on reward through recognized anti/prosociality as a function of three dimensions of the NPI, revealed a significant index with leadership/authority (*B* = -0.99, 95%CI [-1.87, -0.37]), grandiose exhibitionism (*B* = -1.81, 95%CI [-3.42, -0.45]), and entitlement/exploitativeness (*B* = -1.01, 95%CI [-2.00, -0.29]), supporting indirect effects.

### Punishment

**Effects of Leadership/Authority on Punishment.** We ran the ZINB regression in R with the behavioral tendencies condition, leadership/authority, and their interaction as the predictors and punishment as the outcome in both the BL and NB regression models. The BL regression revealed a significant main effect of the co-participant's behavioral tendencies, *B* = -0.72, *p* = .011, 95%CI [-1.28, -0.16], with participants in the antisocial condition (46.09%, *N* = 59 out of 128) being 2.06 times more likely to punish the co-participant than participants in the prosocial condition (32.23%, *N* = 39 out of 121). The main effect of leadership/authority was also significant, *B* = 2.39, *p* < .001, *OR* = 10.99, 95%CI [1.54, 3.25]. However, the interaction was not significant, *B* = 1.44, *p* = .118, *OR* = 4.10, 95%CI [-0.36, 3.18].

The NB regression model revealed that leadership/authority positively predicted punishment, *B* = 0.89, *p* = .009, 95%CI [0.22, 1.56], with participants with high leadership/authority showing a higher degree of punishment in general. The main effect of behavioral tendencies, *B* = -0.09, *p* = .668, 95%CI [-0.51, 0.33], and the interaction effect, *B* = 0.34, *p* = .682, 95%CI [-1.28, 1.96], were not significant.

**Effects of Grandiose Exhibitionism on Punishment.** The BL regression revealed a significant main effect of the co-participant's behavioral tendencies, *B* = -0.96, *p* = .002, 95%CI [-1.57, -0.35], with participants in the antisocial condition (46.09%, *N* = 59 out of 128) being 2.60 times more likely to punish the co-participant than participants in the prosocial condition (32.23%, *N* = 39 out of 121). The main effect of grandiose exhibitionism was also significant, *B* = 3.65, *p* < .001, *OR* = 38.46, 95%CI [2.60, 4.70], however, this was again qualified by a significant interaction. *B* = 3.72, *p* = .002, *OR* = 41.13, 95%CI [1.41, 6.02]. Participants with low grandiose exhibitionism were 10.00 times more likely to punish the co-participant in the antisocial condition than in the prosocial condition, *B* = -2.30, *p* < .001, 95%CI [-3.41, -1.19]; while those with high grandiose exhibitionism displayed no difference (*OR* = 1.05) in punishment likelihood between the two conditions, *B* = -0.05, *p* = .904, 95%CI [-0.88, 0.77].

The NB regression model revealed that the main effects of grandiose exhibitionism, *B* = 0.72, *p* = .051, 95%CI [-0.005, 1.45], and behavioral tendencies, *B* = 0.01, *p* = .980, 95%CI [-0.44, 0.46], and the interaction effect, *B* = 0.25, *p* = .767, 95%CI [-1.40, 1.90], were not significant.

**Effects of Entitlement/Exploitativeness on Punishment.** The BL regression revealed a significant main effect of the co-participant's behavioral tendencies, *B* = -0.67, *p* = .019, 95%CI [-1.24, -0.11], with participants in the antisocial condition (46.09%, *N* = 59 out of 128) being 1.96 times more likely to punish the co-participant than participants in the prosocial condition (32.23%, *N* = 39 out of 121). The main effect of entitlement/exploitativeness was also significant, *B* = 2.49, *p* < .001, *OR* = 12.05, 95%CI [1.66, 3.33], however, this was again qualified by a significant interaction. *B* = 1.87, *p* = .037, *OR* = 6.53, 95%CI [0.11, 3.64]. Participants with low entitlement/exploitativeness were 4.24 times more likely to punish the co-participant in the antisocial condition than in the prosocial condition, *B* = -1.45, *p* = .003, 95%CI [-2.39, -0.50]; while those with high entitlement/exploitativeness displayed no difference in punishment likelihood between the two conditions, *B* = -0.13, *p* = .727, 95%CI [-0.89, 0.62].

The NB regression model revealed that the main effects of entitlement/exploitativeness, *B* = 0.53, *p* = .080, 95%CI [-0.06, 1.13], and behavioral tendencies, *B* = -0.08, *p* = .716, 95%CI [-0.50, 0.35], and the interaction effect, *B* = -0.44, *p* = .539, 95%CI [-1.86, 0.97], were not significant.

**Mediated Moderation Models.** We examined the indirect effects on punishment via recognized anti/prosociality using Model 8 in PROCESS with leadership/authority, grandiose exhibitionism, entitlement/exploitativeness as the moderators. The first step suggested that the interactions between behavioral tendencies and leadership/authority, between behavioral tendencies and grandiose, and behavioral tendencies and entitlement/exploitativeness were significant on recognized anti/prosociality (see above mediated moderation models with reward as the outcome).

The second step showed that recognized anti/prosociality negatively predicted punishment with leadership/authority (*B* = -0.53, *p* ˂ .001, 95%CI [-0.79, -0.28]), grandiose exhibitionism (*B* = -0.65, *p* ˂ .001, 95%CI [-0.96, -0.34]), entitlement/exploitativeness (*B* = -0.54, *p* ˂ .001, 95%CI [-0.80, -0.28]) when controlling for behavioral tendencies, leadership/authority, or grandiose exhibitionism, or entitlement/exploitativeness, and their interaction. The third step, examining the indirect effect of behavioral tendencies on punishment through recognized anti/prosociality as a function of three dimensions of the NPI, revealed a significant index with leadership/authority (*B* = 1.31, 95%CI [0.65, 2.22]), grandiose exhibitionism (*B* = 2.95, 95%CI [1.34, 5.12]), and entitlement/exploitativeness (*B* = 1.57, 95%CI [0.71, 2.79]), supporting indirect effects.

## Study 4

### Moral Character Evaluation

Effects of Leadership/Authority on Moral Character Evaluation. We used Model 1 in PROCESS to test the effects the co-participant’s behavioral tendencies and leadership/authority on moral character evaluation. The results yielded a significant main effect of the behavioral tendencies, *B* = 1.18, *t*(238) = 6.94, *p* ˂ .001, *r* = .41, 95%CI [0.85, 1.52], specifically, participants in the prosocial condition (*M* = 5.73, *SD* = 1.00) rated their co-participant to be more moral than those in the antisocial condition (*M* = 4.56, *SD* = 1.74). The main effect of leadership/authority was also significant, *B* = 1.17, *t*(238) = 4.39, *p* ˂ .001, *r* = .27, 95%CI [0.64, 1.69]. The two-way interaction between behavioral tendencies and leadership/authority was also significant, *B* = -2.39, *t*(238) = -4.49, *p* ˂ .001, *r* = .28, 95%CI [-3.43, -1.34]. Results of simple slope analysis showed that participants with low leadership/authority rated their co-participant in the prosocial condition as more moral than the one in the antisocial condition, *B* = 1.95, *t*(238) = 8.07, *p* ˂ .001, *r* = .46, 95%CI [1.48, 2.43], while the difference was not significant for those with high leadership/authority, *B* = 0.41, *t*(238) = 1.71, *p* = .088, *r* = .11, 95%CI [-0.06, 0.89].

**Effects of Grandiose Exhibitionism on** Moral Character Evaluation**.** We used Model 1 in PROCESS to test the effects the co-participant’s behavioral tendencies and grandiose exhibitionism on moral character evaluation. The results yielded a significant main effect of the behavioral tendencies, *B* = 1.18, *t*(238) = 7.19, *p* ˂ .001, *r* = .42, 95%CI [0.86, 1.50], specifically, participants in the prosocial condition (*M* = 5.73, *SD* = 1.00) rated their co-participant to be more moral than those in the antisocial condition (*M* = 4.56, *SD* = 1.74). The main effect of grandiose exhibitionism was also significant, *B* = 1.40, *t*(238) = 5.77, *p* ˂ .001, *r* = .35, 95%CI [0.92, 1.88]. The two-way interaction between behavioral tendencies and grandiose exhibitionism was also significant, *B* = -2.60, *t*(238) = -5.36, *p* ˂ .001, *r* = .33, 95%CI [-3.56, -1.65]. Results of simple slope analysis showed that participants with low grandiose exhibitionism rated their co-participant in the prosocial condition as more moral than the one in the antisocial condition, *B* = 2.06, *t*(238) = 8.87, *p* ˂ .001, *r* = .50, 95%CI [1.61, 2.52], while the difference was not significant for those with high grandiose exhibitionism, *B* = 0.30, *t*(238) = 1.27, *p* = .204, *r* = .08, 95%CI [-0.16, 0.75].

**Effects of Entitlement/Exploitativeness on** Moral Character Evaluation**.** We used Model 1 in PROCESS to test the effects the co-participant’s behavioral tendencies and entitlement/exploitativeness on moral character evaluation. The results yielded a significant main effect of the behavioral tendencies, *B* = 1.17, *t*(238) = 7.15, *p* ˂ .001, *r* = .42, 95%CI [0.85, 1.49], specifically, participants in the prosocial condition (*M* = 5.73, *SD* = 1.00) rated their co-participant to be more moral than those in the antisocial condition (*M* = 4.56, *SD* = 1.74). The main effect of entitlement/exploitativeness was also significant, *B* = 1.12, *t*(238) = 5.36, *p* ˂ .001, *r* = .33, 95%CI [0.77, 1.67]. The two-way interaction between behavioral tendencies and entitlement/exploitativeness was also significant, *B* = -2.65, *t*(238) = -5.82, *p* ˂ .001, *r* = .35, 95%CI [-3.55, -1.75]. Results of simple slope analysis showed that participants with low entitlement/exploitativeness rated their co-participant in the prosocial condition as more moral than the one in the antisocial condition, *B* = 2.12, *t*(238) = 9.17, *p* ˂ .001, *r* = .51, 95%CI [1.67, 2.58], while the difference was not significant for those with high entitlement/exploitativeness, *B* = 0.21, *t*(238) = 0.93, *p* = .356, *r* = .06, 95%CI [-0.24, 0.67].

### Reward

**Effects of** Leadership/Authority **on Reward**. The results of Model 1 (PROCESS) revealed a significant main effect of behavioral tendencies on reward, such that participants in the prosocial condition (78.51%, *N* = 95 out of 121) were 1.81 times more likely to offer a high (vs. low) reward to the co-participant than those in the antisocial condition (66.94%, *N* = 81 out of 121), *B* = 0.59, *p* = .044, 95%CI [0.02, 1.17]. The main effect of leadership/authority, *B* = 0.15, *p* = .747, *OR* = 1.16, 95%CI [-0.74, 1.03,], and the interaction were not significant, *B* = -0.78, *p* = .397, *OR* = 0.46, 95%CI [-2.57, 1.02].

**Effects of Grandiose Exhibitionism on Reward.** The results of Model 1 (PROCESS) revealed a significant main effect of behavioral tendencies on reward, such that participants in the prosocial condition (78.51%, *N* = 95 out of 121) were 1.80 times more likely to offer a high (vs. low) reward to the co-participant than those in the antisocial condition (66.94%, *N* = 81 out of 121), *B* = 0.59, *p* = .045, 95%CI [0.01, 1.17]. The main effect of grandiose exhibitionism, *B* = -0.20, *p* = .645, *OR* = 0.82, 95%CI [-1.05, 0.65], and the interaction were not significant, *B* = -0.71, *p* = .413, *OR* = 0.49, 95%CI [-2.42, 1.00].

**Effects of Entitlement/Exploitativeness on Reward.** The results of Model 1 (PROCESS) revealed a significant main effect of behavioral tendencies on reward, such that participants in the prosocial condition (78.51%, *N* = 95 out of 121) were 1.81 times more likely to offer a high (vs. low) reward to the co-participant than those in the antisocial condition (66.94%, *N* = 81 out of 121), *B* = 0.59, *p* = .044, 95%CI [0.02, 1.17]. The main effect of entitlement/exploitativeness, *B* = 0.55, *p* = .176, *OR* = 1.73, 95%CI [-0.24, 1.34,], and the interaction were not significant, *B* = -1.02, *p* = .216, *OR* = 0.36, 95%CI [-2.63, 0.59].

**Mediated Moderation Models.** We examined the indirect effects on reward via recognized anti/prosociality using Model 8 in PROCESS with leadership/authority, grandiose exhibitionism, entitlement/exploitativeness as the moderators. The first step suggested that the interactions between behavioral tendencies and leadership/authority (*B* = -3.49, *t*(238) = -8.85, *p* <.001, *r* = .50, 95%CI [-4.27, -2.72]), and between behavioral tendencies and grandiose exhibitionism (*B* = -3.76, *t*(238) = -10.58, *p* <.001, *r* = .57, 95%CI [-4.46, -3.06]), and behavioral tendencies and entitlement/exploitativeness (*B* = -3.53, *t*(238) = -10.50, *p* <.001, *r* = .56, 95%CI [-4.20, -2.87]) were significant on recognized anti/prosociality. Specifically compared with participants with low leadership/authority (*B* = 2.80, *t*(238) = 15.56, *p* < .001, *r* = .71, 95%CI [2.44, 3.15]), low grandiose exhibitionism (*B* = 2.95, *t*(238) = 17.29, *p* < .001, *r* = .75, 95%CI [2.61, 3.28]), or low entitlement/exploitativeness (*B* = 2.94, *t*(238) = 17.20, *p* < .001, *r* = .74, 95%CI [2.61, 3.08]), those with high leadership/authority (*B* = 0.55, *t*(238) = 3.04, *p* = .003, *r* = .19, 95%CI [0.19, 0.90]), high grandiose exhibitionism (*B* = 0.39, *t*(238) = 2.30, *p* = .022, *r* = .15, 95%CI [0.06, 0.73]), or high entitlement/exploitativeness (*B* = 0.40, *t*(238) = 2.34, *p* = .020, *r* = .15, 95%CI [0.06, 0.74]) displayed a smaller difference in recognized anti/prosociality between the two conditions.

The second step showed that recognized anti/prosociality positively predicted reward with leadership/authority (*B* = 0.35, *p* = .020, 95%CI [0.05, 0.65]), grandiose exhibitionism (*B* = 0.39, *p* = .015, 95%CI [0.08, 0.71]), entitlement/exploitativeness (*B* = 0.32, *p* = .046, 95%CI [0.006, 0.63]) when controlling for behavioral tendencies, leadership/authority, or grandiose exhibitionism, or entitlement/exploitativeness, and their interaction. The third step, examining the indirect effect of behavioral tendencies on reward through recognized anti/prosociality as a function of three dimensions of the NPI, revealed a significant index with leadership/authority (*B* = -1.24, 95%CI [-2.57, -0.11]), grandiose exhibitionism (*B* = -1.48, 95%CI [-2.94, -0.18]), and entitlement/exploitativeness (*B* = -1.13, 95%CI [-2.39, -0.14]), supporting indirect effects.

### Punishment

**Effects of Leadership/Authority on Punishment.** We ran the ZINB regression in R with the behavioral tendencies condition, leadership/authority, and their interaction as the predictors and punishment as the outcome in both the BL and NB regression models. The BL regression revealed a significant main effect of the co-participant's behavioral tendencies, *B* = -0.84, *p* = .010, 95%CI [-1.48, -0.20], with participants in the antisocial condition (76.03%, *N* = 92 out of 121) being 2.31 times more likely to punish the co-participant than participants in the prosocial condition (61.16%, *N* = 74 out of 121). The main effect of leadership/authority was also significant, *B* = 3.56, *p* < .001, *OR* = 35.71, 95%CI [2.52, 4.61]. However, the interaction was not significant, *B* = 0.99, *p* = .359, *OR* = 2.68, 95%CI [-1.12, 3.09].

The NB regression model revealed that the main effects of leadership/authority, *B* = 0.25, *p* = .425, 95%CI [-0.37, 0.88], and behavioral tendencies, *B* = 0.02, *p* = .883, 95%CI [-0.29, 0.34], and the interaction effect, *B* = 0.17, *p* = .789, 95%CI [-1.08, 1.42], were not significant.

**Effects of Grandiose Exhibitionism on Punishment.** The BL regression revealed a significant main effect of the co-participant's behavioral tendencies, *B* = -1.00, *p* = .005, 95%CI [-1.70, -0.30], with participants in the antisocial condition (76.03%, *N* = 92 out of 121) being 2.72 times more likely to punish the co-participant than participants in the prosocial condition (61.16%, *N* = 74 out of 121). The main effect of grandiose exhibitionism was also significant, *B* = 4.88, *p* < .001, *OR* = 125.00, 95%CI [3.63, 6.13]. However, the interaction was not significant, *B* = 1.11, *p* = .384, *OR* = 3.03, 95%CI [-1.69, 3.61].

The NB regression model revealed that the main effects of grandiose exhibitionism, *B* = 0.21, *p* = .482, 95%CI [-0.37, 0.78], and behavioral tendencies, *B* = 0.02, *p* = .883, 95%CI [-0.29, 0.34], and the interaction effect, *B* = 0.28, *p* = .638, 95%CI [-0.88, 1.43], were not significant.

**Effects of Entitlement/Exploitativeness on Punishment.** The BL regression revealed a significant main effect of the co-participant's behavioral tendencies, *B* = -1.04, *p* = .003, 95%CI [-1.73, 0.35], with participants in the antisocial condition (76.03%, *N* = 92 out of 121) being 2.82 times more likely to punish the co-participant than participants in the prosocial condition (61.16%, *N* = 74 out of 121). The main effect of entitlement/exploitativeness was also significant, *B* = 4.04, *p* < .001, *OR* = 56.76, 95%CI [2.98, 5.10]. However, the interaction was not significant, *B* = 0.05, *p* = .963, *OR* = 1.05, 95%CI [-2.08, 2.18].

The NB regression model revealed that the main effects of entitlement/exploitativeness, *B* = 0.13, *p* = .647, 95%CI [-0.42, 0.67], and behavioral tendencies, *B* = 0.02, *p* = .900, 95%CI [-0.29, 0.34], and the interaction effect, *B* = -0.17, *p* = .767, 95%CI [-1.28, 0.94], were not significant.

**Mediated Moderation Models.** We examined the indirect effects on punishment via recognized anti/prosociality using Model 8 in PROCESS with leadership/authority, grandiose exhibitionism, entitlement/exploitativeness as the moderators. The first step suggested that the interactions between behavioral tendencies and leadership/authority, between behavioral tendencies and grandiose exhibitionism, and behavioral tendencies and entitlement/exploitativeness were significant on recognized anti/prosociality (see the mediated moderation models with reward as the outcome).

The second step showed that recognized anti/prosociality negatively predicted punishment with leadership/authority (*B* = -0.57, *p* < .001, 95%CI [-0.90, -0.24]), grandiose exhibitionism (*B* = -0.56, *p* = .002, 95%CI [-0.92, -0.21]), entitlement/exploitativeness (*B* = -0.81, *p* < .001, 95%CI [-1.19, -0.44]) when controlling for behavioral tendencies, leadership/authority, or grandiose exhibitionism, or entitlement/exploitativeness, and their interaction. The third step, examining the indirect effect of behavioral tendencies on punishment through recognized anti/prosociality as a function of three dimensions of the NPI, revealed a significant index with leadership/authority (*B* = 2.00, 95%CI [0.43, 4.25]), grandiose exhibitionism (*B* = 2.12, 95%CI [0.36, 4.45]), and entitlement/exploitativeness (*B* = 2.87, 95%CI [1.17, 5.48]), supporting indirect effects.

## Conclusion

The three subscales of the NPI showed similar effects as the global NPI on individuals’ responsiveness to others’ anti/prosocialitys regarding moral character evaluations. As for reward and punishment, while the overall moderation effects of leadership/authority and grandiose exhibitionism on reward, and the overall moderation effect of leadership/authority on punishment were not significant in Study 3, these three subscales showed consistent effects as the global NPI on all the other results, including the indirect effects on reward and punishment through recognized anti/prosociality.

# Effects of the NARQ (Admiration, Rivalry) on Participants’ Responsiveness

The NARQ consists of two dimensions of grandiose narcissism each with 9 items, namely admiration (α = .91, in Study 1; α = .93, in Study 4) and rivalry (α = .87, in Study 1; α = .95, in Study 4). Participants rated whether each of 18 items applied to them (e.g., “I am great”; 1 = *Strongly disagree*, 6 = *Strongly agree*). We included rivalry as a covariate when examining the effects of admiration and vice versa.

## Study 1

### Moral Character Evaluation

**Effects of Admiration on Moral Character Evaluation**. We used Model 3 in PROCESS to test the effects of actor’s behavior, admiration, and self-relevance on moral character evaluation while controlling for rivalry. The results yielded a significant main effect of actor’s behavior, *B* = -2.56, *t*(435) = -22.39, *p* ˂ .001, *r* = .73, 95%CI [-2.79, -2.34], specifically, participants in the antisocial condition (*M* = 2.07, *SD* = 1.36) rated the actor to be less moral than those in the control condition (*M* = 4.62, *SD* = 1.08). The main effect of admiration was not significant, *B* = 0.11, *t*(435) = 1.72, *p* = .085, *r* = .08, 95%CI [-0.01, 0.22], nor of self-relevance, *B* = 0.07, *t*(435) = 0.57, *p* = .566, *r* = .03, 95%CI [-0.16, 0.29]. The three-way interaction, *B* = 0.24, *t*(435) = 1.11, *p* = .267, *r* = .05, 95%CI [-0.19, 0.67], and the two-way interactions between self-relevance and actor’s behavior, *B* = 0.17, *t*(435) = 0.73, *p* = .463, *r* = .03, 95%CI [-0.28, 0.62], and between self-relevance and admiration, *B* = 0.0004, *t*(435) = 0.003, *p* = .997, *r* = .0001, 95%CI [-0.21, 0.21], were not significant. However, the two-way interaction between actor’s behavior and admiration was significant, *B* = 0.49, *t*(435) = 4.47, *p* ˂ .001, *r* = .21, 95%CI [0.27, 0.70]. Results of simple slope analysis showed that both participants with low, *B* = -3.08, *t*(439) = -19.18, *p* ˂ .001, *r* = .68, 95%CI [-3.40, -2.77], and high admiration, *B* = -2.02, *t*(439) = -12.59, *p* ˂ .001, *r* = .52, 95%CI [-2.34, -1.71], rated the actor in the control condition as more moral than the one in the antisocial condition, but the difference was smaller for those with high admiration.

**Effects of Rivalry on Moral Character Evaluation**. We used Model 3 in PROCESS to test the effects of actor’s behavior, rivalry, and self-relevance on moral character evaluation while controlling for admiration. The results yielded a significant main effect of actor’s behavior, *B* = -2.56, *t*(435) = -22.84, *p* ˂ .001, *r* = .74, 95%CI [-2.78, -2.34], specifically, participants in the antisocial condition (*M* = 2.07, *SD* = 1.36) rated the actor to be less moral than those in the control condition (*M* = 4.62, *SD* = 1.08). There was no significant main effect of rivalry, *B* = 0.05, *t*(435) = 0.70, *p* = .119, *r* = .03, 95%CI [-0.09, 0.18], nor of self-relevance, *B* = 0.09, *t*(435) = 0.82, *p* = .410, *r* = .04, 95%CI [-0.13, 0.31]. The three-way interaction, *B* = 0.32, *t*(435) = 1.30, *p* = .195, *r* = .06, 95%CI [-0.16, 0.80], and the two-way interactions between self-relevance and actor’s behavior, *B* = 0.13, *t*(435) = 0.58, *p* = .559, *r* = .03, 95%CI [-0.31, 0.57], and between self-relevance and rivalry, *B* = -0.004, *t*(435) = -0.03, *p* = 0.977, *r* = .001, 95%CI [-0.24, 0.24], were not significant. However, the two-way interaction between actor’s behavior and rivalry was significant, *B* = 0.74, *t*(435) = 6.07, *p* ˂ .001, *r* = .28, 95%CI [0.50, 0.98]. Results of simple slope analysis showed that both participants with low, *B* = -3.25, *t*(439) = -20.61, *p* ˂ .001, *r* = .70, 95%CI [-3.56, -2.94], and high rivalry, *B* = -1.85, *t*(439) = -11.72, *p* ˂ .001, *r* = .49, 95%CI [-2.16, -1.54], rated the actor in the control condition as more moral than the one in the antisocial condition, but the difference was smaller for those with high rivalry.

## Study 4

### Moral Character Evaluation

**Effects of Admiration on Moral Character Evaluation**. We used Model 1 in PROCESS to test the effects the co-participant’s behavioral tendencies and admiration on moral character evaluation while controlling for rivalry. The results yielded a significant main effect of the behavioral tendencies, *B* = 1.28, *t*(237) = 8.28, *p* ˂ .001, *r* = .47, 95%CI [0.98, 1.59], specifically, participants in the prosocial condition (*M* = 5.73, *SD* = 1.00) rated their co-participant to be more moral than those in the antisocial condition (*M* = 4.56, *SD* = 1.74). The main effect of admiration was also significant, *B* = 0.44, *t*(237) = 3.95, *p* ˂ .001, *r* = .25, 95%CI [0.22, 0.65]. The two-way interaction between behavioral tendencies and admiration was also significant, *B* = -0.83, *t*(237) = -6.39, *p* ˂ .001, *r* = .38, 95%CI [-1.08, -0.57]. Results of simple slope analysis showed that participants with low admiration rated their co-participant in the prosocial condition as more moral than the one in the antisocial condition, *B* = 2.28, *t*(237) = 10.33, *p* ˂ .001, *r* = .56, 95%CI [1.84, 2.71], while the difference was not significant for those with high admiration, *B* = 0.29, *t*(237) = 1.30, *p* = .194, *r* = .08, 95%CI [-0.15, 0.72].

**Effects of Rivalry on Moral Character Evaluation**. We used Model 1 in PROCESS to test the effects the co-participant’s behavioral tendencies and rivalry on moral character evaluation while controlling for admiration. The results yielded a significant main effect of the behavioral tendencies, *B* = 1.28, *t*(237) = 8.57, *p* ˂ .001, *r* = .49, 95%CI [0.98, 1.57], specifically, participants in the prosocial condition (*M* = 5.73, *SD* = 1.00) rated their co-participant to be more moral than those in the antisocial condition (*M* = 4.56, *SD* = 1.74). The main effect of rivalry was not significant, *B* = 0.13, *t*(237) = 1.39, *p* = .167, *r* = .09, 95%CI [-0.05, 0.31]. The two-way interaction between behavioral tendencies and rivalry was also significant, *B* = -0.84, *t*(237) = -7.82, *p* ˂ .001, *r* = .45, 95%CI [-1.04, -0.62]. Results of simple slope analysis showed that participants with low rivalry rated their co-participant in the prosocial condition as more moral than the one in the antisocial condition, *B* = 2.45, *t*(237) = 11.55, *p* ˂ .001, *r* = .60, 95%CI [2.03, 2.87], while the difference was not significant for those with high rivalry, *B* = 0.11, *t*(237) = 0.51, *p* = .609, *r* = .03, 95%CI [-0.31, 0.52].

### Reward

**Effects of Admiration on Reward.** We employed Model 1 in PROCESS to examine the effects of co-participant’s behavioral tendencies and admiration on reward while controlling for rivalry. The results revealed a significant main effect of behavioral tendencies, *B* = 0.63, *p* = .037, 95%CI [0.04, 1.22], such that participants in the prosocial condition (78.51%, *N* = 95 out of 121) were 1.98 times more likely to offer a high (vs. low) reward to their co-participant than those in the antisocial condition (66.94%, *N* = 81 out of 121). The main effect of admiration, *B* = -0.18, *p* = .381, *OR* = 0.84, 95%CI [-0.58, 0.22], and interaction effect, *B* = -0.43, *p* = .087, *OR* = 0.65, 95%CI [-0.93, 0.06] were both not significant.

**Effects of Rivalry on Reward.** We employed Model 1 in PROCESS to examine the effects of co-participant’s behavioral tendencies and rivalry on reward while controlling for admiration. The results revealed a significant main effect of behavioral tendencies, *B* = 0.61, *p* = .044, 95%CI [0.02, 1.21], such that participants in the prosocial condition (78.51%, *N* = 95 out of 121) were 1.98 times more likely to offer a high (vs. low) reward to their co-participant than those in the antisocial condition (66.94%, *N* = 81 out of 121). The main effect of rivalry was also significant, *B* = 0.41, *p* = .020, *OR* = 153, 95%CI [0.07, 0.76]. However, the interaction effect was not significant, *B* = -0.31, *p* = .149, *OR* = 0.73, 95%CI [-0.73, 0.11].

**Mediated Moderation Models.** Despite that the interaction between behavioral tendencies and admiration, and between behavioral tendencies and rivalry did not show significantly total effects on reward, we examined the indirect effects via recognized anti/prosociality using Model 8 in PROCESS while controlling for rivalry when analyzing the admiration and vice versa. The first step suggested that the interactions between behavioral tendencies and admiration (*B* = -1.18, *t*(237) = -12.43, *p* <.001, *r* = .63, 95%CI [-1.36, -0.99]), and between behavioral tendencies and rivalry (*B* = -1.12, *t*(237) = -15.25, *p* <.001, *r* = .70, 95%CI [-1.27, -0.98]), were significant on recognized anti/prosociality. Specifically participants with low admiration (*B* = 3.08, *t*(237) = 19.14, *p* < .001, *r* = .78, 95%CI [2.76, 3.40]), or low rivalry (*B* = 3.24, *t*(237) = 22.13, *p* < .001, *r* = .82, 95%CI [2.95, 3.53]) were more likely to offer high reward to the co-participant in the prosocial condition than in the antisocial condition, whereas those with high admiration (*B* = 0.25, *t*(237) = 1.57, *p* = .117, *r* = .10, 95%CI [-0.06, 0.57]), or high rivalry (*B* = 0.09, *t*(237) = 0.59, *p* = .559, *r* = .04, 95%CI [-0.20, 0.37]) did not show significant difference in rewarding in the two conditions.

The second step showed that recognized anti/prosociality significantly predicted reward with admiration (*B* = 0.34, *p* = .041, 95%CI [0.01, 0.67]), and with rivalry (*B* = 0.43, *p* = .022, 95%CI [0.06, 0.81]) when controlling for behavioral tendencies, admiration or rivalry, and their interaction. The third step, examining the indirect effect of behavioral tendencies on reward through recognized anti/prosociality as a function of admiration or rivalry, revealed a non-significant index with admiration (*B* = -0.41, 95%CI [-0.90, 0.01]) but a significant index with rivalry (*B* = -0.49, 95%CI [-1.04, -0.04]), therefore only providing support for indirect effects with rivalry.

### Punishment

**Effects of Admiration on Punishment.** We ran the ZINB regression model in R while controlling for rivalry. The results of BL regression model revealed a significant main effect of behavioral tendencies, *B* = -1.04, *p* = .039, 95%CI [-2.02, -0.05], with participants in the antisocial condition (76.03%, *N* = 92 out of 121) being 2.83 times more likely to punish their co-participant than those in the prosocial condition (61.16%, *N* = 74 out of 121). The main effect of admiration and the interaction were not significant, *B* = 0.27, *p* = .278, *OR* = 1.32, 95%CI [-0.22, 0.77], *B* = 0.003, *p* = .995, *OR* = 1.00, 95%CI [-0.84, 0.84].

The results of NB regression model showed that the main effects of behavioral tendencies, *B* = -0.03, *p* = .861, 95%CI [-0.34, 0.29] and admiration, *B* = -0.18, *p* = .316, 95%CI [-0.53, 0.17], and the interaction effect, *B* = -0.06, *p* = .586, 95%CI [-0.45, 0.33] were all not significant.

**Effects of Rivalry on Punishment.** We ran the ZINB regression model in R while controlling for admiration. The results of BL regression model revealed a significant main effect of behavioral tendencies, *B* = -1.03, *p* = .019, 95%CI [-1.90,-0.17], with participants in the antisocial condition (76.03%, *N* = 92 out of 121) being 2.82 times more likely to punish the co-participant than participants in the prosocial condition (61.16%, *N* = 74 out of 121). The main effect of rivalry was also significant, *B* = 1.68, *p* < .001, *OR* =5.34, 95%CI [[1.15, 2.22]. However the interaction effect was not significant, *B* = 0.49, *p* = .287, *OR* = 1.63, 95%CI [-0.41, 1.40].

The results of NB regression model showed that the main effects of behavioral tendencies, *B* = -0.03, *p* = .861, 95%CI [-0.34, 0.29] and rivalry, *B* = 0.26, *p* = .084, 95%CI [-0.04, 0.56], and the interaction effect, *B* = -0.06, *p* = .728, 95%CI [-0.41, 0.29] were all not significant.

**Mediated Moderation Models.** Despite that the interaction between behavioral tendencies and admiration, and between behavioral tendencies and rivalry did not show significantly total effects on punishment, we examined the indirect effects via recognized anti/prosociality using Model 8 in PROCESS. The first step suggested that the interactions between behavioral tendencies and admiration, and between behavioral tendencies and rivalry were significant on recognized anti/prosociality (see above mediated moderation models with the reward as the outcome).

The second step showed that recognized anti/prosociality significantly predicted punishment with admiration (*B* = -0.67, *p* = .004, 95%CI [-1.13, -0.21]), and with rivalry (*B* = -0.61, *p* = .018, 95%CI [-1.12, -0.10]) when controlling for behavioral tendencies, admiration, or rivalry, and their interaction. The third step, examining the indirect effect of behavioral tendencies on punishment through recognized anti/prosociality as a function of admiration and rivalry respectively, revealed a significant index for admiration (*B* = 0.78, 95%CI [0.14, 1.71]), but a non-significant index for rivalry (*B* = 0.69, 95%CI [-0.06, 1.82]), therefore providing support for indirect effects with admiration, but not with rivalry.

## Conclusion

Admiration and rivalry dimensions of the NARQ showed similar effects as the global NPI on individuals’ responsiveness to others’ social behaviors regarding moral character evaluations and similar overall effects on reward, and punishment. However, in terms of the indirect effects of recognized anti/prosociality on reward and punishment, recognized anti/prosociality could only explain the interaction effect between behavioral tendencies and admiration on punishment.

# Results When Controlling for Participants’ Big Five Personalities

In order to disentangle the effects of participants’ big five personalities, especially agreeableness and extraversion, on their responsiveness to others’ social behaviors, we reanalyzed the data when controlling for agreeableness and extraversion in Study 1 and 4, and agreeableness in Study 2 and 3. The reliability of agreeableness was as follows in each study: α = .82, in Study 1; α = .84, in Study 2; α = .84, in Study 3; α = .10, in Study 4. The reliability of extraversion: α = .85, in Study 1; α = .20, in Study 4. Please note that the reliability of agreeableness and extraversion in Study 4 were very low because we measured participants’ big five personality traits with the Ten-Item Personality Inventory (TIPI; TIPI; Gosling, Rentfrow, & Swann, 2003) in Study 4, with each trait being measured with only two items. The TIPI did not aim to optimize the typical internal consistency, but rather emphasize validity considerations, resulting in lower inter-item correlations than that of typical scales (Gosling, Rentfrow, & Swann, 2003). However, the TIPI has demonstrated adequate test-retest reliabilities and substantial convergent validity with longer Big Five measures (Azkhosh, Sahaf, Rostami, & Ahmadi, 2019). In Study 4, narcissism was strongly correlated with agreeableness (*r* = -.29, *p* ˂ .01) and extraversion (*r* = .39, *p* ˂ .01), which was in the expected direction and in line with previous studies and thus lends further support to the construct validity of these two measures.

## Study 1

### Moral Character Evaluation

We used Model 3 in PROCESS to test the effects of actor’s behavior, narcissism, and self-relevance on moral character evaluation with agreeableness and extraversion as the covariates. The results yielded a significant main effect of actor’s behavior, *B* = -2.55, *t*(434) = -22.30, *p* ˂ .001, *r* = .73, 95%CI [-2.78, -2.33], specifically, participants in the antisocial condition (*M* = 2.07, *SD* = 1.36) rated the actor to be less moral than those in the control condition (*M* = 4.62, *SD* = 1.08). There was no significant main effect of narcissism, *B* = 0.16, *t*(434) = 0.52, *p* = .606, *r* = .02, 95%CI [-0.46, 0.78], nor of self-relevance, *B* = 0.07, *t*(434) = 0.65, *p* = .514, *r* = .03, 95%CI [-0.15, 0.30]. The three-way interaction, *B* = 0.17, *t*(434) = 1.63, *p* = .104, *r* = .08, 95%CI [-0.03, 0.37], and the two-way interactions between self-relevance and actor’s behavior, *B* = 0.15, *t*(434) = 0.65, *p* = .518, *r* = .03, 95%CI [-0.30, 0.60], and between self-relevance and narcissism, *B* = 0.14, *t*(434) = 0.30, *p* = .762, *r* = .01, 95%CI [-0.77, 1.05], were not significant. However, the two-way interaction between actor’s behavior and narcissism was significant, *B* = 2.16, *t*(434) = 4.69, *p* ˂ .001, *r* = .22, 95%CI [1.26, 3.07]. Results of simple slope analysis showed that both participants with low, *B* = -3.10, *t*(438) = -19.25, *p* ˂ .001, *r* = .68, 95%CI [-3.42, -2.78], and high narcissism, *B* = -1.99, *t*(438) = -12.43, *p* ˂ .001, *r* = .51, 95%CI [-2.30, -1.68], rated the actor in the control condition as more moral than the one in the antisocial condition, but the difference was smaller for those with high narcissism.

## Study 2

### Moral Character Evaluation

We used Model 1 in PROCESS to test the effects of actor’s behavior and narcissism on moral character evaluation with agreeableness as the covariate. The results yielded a significant main effect of actor’s behavior, *B* = 1.48, *t*(244) = 8.85, *p* ˂ .001, *r* = .49, 95%CI [1.15, 1.81], specifically, participants in the prosocial condition (*M* = 5.68, *SD* = 1.20) rated the actor to be more moral than those in the control condition (*M* = 4.21, *SD* = 1.46). There was no significant main effect of narcissism, *B* = 0.57, *t*(244) = 1.71, *p* = .088, *r* = .11, 95%CI [-0.09, 1.22]. However, the two-way interaction between actor’s behavior and narcissism was significant, *B* = -1.59, *t*(244) = -2.77, *p* = .006, *r* = .17, 95%CI [-2.71, -0.46]. Results of simple slope analysis showed that both participants with low, *B* = 1.94, *t*(244) = 8.21, *p* ˂ .001, *r* = .47, 95%CI [1.48, 2.41], and high narcissism, *B* = 1.01, *t*(244) = 4.28, *p* ˂ .001, *r* = .26, 95%CI [0.55, 1.48], rated the actor in the prosocial condition as more moral than the one in the control condition, but the difference was smaller for those with high narcissism.

## Study 3

### Moral Character Evaluation

We used Model 1 in PROCESS to test the effects the co-participant’s behavioral tendencies and narcissism on moral character evaluation with agreeableness as the covariate. The results yielded a significant main effect of the behavioral tendencies, *B* = 3.05, *t*(244) = 16.44, *p* ˂ .001, *r* = .72, 95%CI [2.68, 3.42], specifically, participants in the prosocial condition (*M* = 5.93, *SD* = 1.09) rated their co-participant to be more moral than those in the antisocial condition (*M* = 2.85, *SD* = 1.68). There was no significant main effect of narcissism, *B* = 0.66, *t*(244) = 1.73, *p* = .085, *r* = .11, 95%CI [-0.09, 1.41]. However, the two-way interaction between behavioral tendencies and narcissism was significant, *B* = -3.04, *t*(244) = -4.46, *p* ˂ .001, *r* = .27, 95%CI [-4.38, -1.70]. Results of simple slope analysis showed that both participants with low, *B* = 3.89, *t*(244) = 14.65, *p* ˂ .001, *r* = .68, 95%CI [3.37, 4.42], and high narcissism, *B* = 2.21, *t*(244) = 8.35, *p* ˂ .001, *r* = .47, 95%CI [1.68, 2.73], rated their co-participant in the prosocial condition as more moral than the one in the antisocial condition, but the difference was smaller for those with high narcissism.

### Reward

When controlling for agreeableness, the results of Model 1 (PROCESS) revealed a significant main effect of behavioral tendencies on reward, such that participants in the prosocial condition (68.60%, *N* = 83 out of 121) were 4.53 times more likely to offer a high (vs. low) reward to the co-participant than those in the antisocial condition (32.81%, *N* = 42 out of 128), *B* = 1.51, *p* < .001, 95%CI [0.97, 2.06]. The main effect of narcissism was also significant, *B* = 1.48, *p* = .194, *OR* = 4.41, 95%CI [0.35, 2.62]. The interaction was also significant, *B* = -2.19, *p* = .036, *OR* = 0.11, 95%CI [-4.23, -0.14]. Results of simple slope analysis showed that participants with low narcissism were 8.50 times more likely to give high reward to their co-participants in the prosocial condition than the one in the antisocial condition, *B* = 2.14, *p* ˂ .001, 95%CI [1.32, 2.97], while those with high narcissism were only 2.53 times more likely to give high reward to their co-participants in the prosocial condition than the one in the antisocial condition, *B* = 0.93, *p* =.016, 95%CI [0.18, 1.68].

### Punishment

When controlling for agreeableness, the BL regression revealed a significant main effect of the co-participant's behavioral tendencies, *B* = -0.98, *p* = .003, 95%CI [-1.61, -0.34], with participants in the antisocial condition (46.09%, *N* = 59 out of 128) being 2.65 times more likely to punish the co-participant than participants in the prosocial condition (32.23%, *N* = 39 out of 121). The main effect of narcissism was also significant, *B* = 2.91, *p* < .001, *OR* = 18.52, 95%CI [1.68, 4.14]. However, the interaction was not significant, *B* = 2.06, *p* = .115, *OR* = 7.87, 95%CI [-0.50, 4.63].

The NB regression model revealed that the main effects of narcissism, *B* = 0.88, *p* = .057, 95%CI [-0.03, 1.79], and behavioral tendencies, *B* = 0.06, *p* = .802, 95%CI [-0.39, 0.51], and the interaction effect, *B* = 0.10, *p* = .918, 95%CI [-1.82, 2.03], were not significant.

### Mediated Moderation Model

We investigated whether participants’ recognized anti/prosociality could still explain their reward and punishment responses using Model 8 in PROCESS when controlling for participants’ agreeableness. The first step indicated a significant interaction effect between behavioral tendencies and narcissism on recognized anti/prosociality, *B* = -4.24, *t*(244) = -8.28, *p* <.001, *r* = .47, 95%CI [-5.24, -3.23]. Specifically, compared with low narcissists, *B* = 4.29, *t*(244) = 21.52, *p* < .001, *r* = .81, 95%CI [3.90, 4.69], high narcissists displayed a smaller difference in recognized anti/prosociality between the two conditions, *B* = 1.94, *t*(244) = 9.80, *p* < .001, *r* = .53, 95%CI [1.55, 2.33].

The second step showed that recognized anti/prosociality positively predicted reward (*B* = 0.37, *p* = .004, 95%CI [0.12, 0.63]), and negatively predicted punishment (*B* = -0.68, *p* < .001, 95%CI [-0.99, -0.38]) respectively when controlling for co-participants’ behavioral tendencies, narcissism, and their interaction. The third step, examining the indirect effect of behavioral tendencies on reward and punishment through recognized anti/prosociality as a function of narcissism, revealed a significant index with reward (*B* = -1.58, 95%CI [-3.11, -0.43]), and punishment (*B* = 2.90, 95%CI [1.57, 4.79]), supporting indirect effects.

## Study 4

### Moral Character Evaluation

It should be noted that, we used the cognitive task as the buffer measure rather than the NEO-FFI. However, we also measured participants’ big five personalities with the Ten-Item Personality Inventory (TIPI; Gosling, Rentfrow, & Swann, 2003), which enabled us to control their agreeableness and extraversion when examining effects of narcissism on responsiveness. We used Model 1 in PROCESS to test the effects the co-participant’s behavioral tendencies and narcissism on moral character evaluation. The results yielded a significant main effect of the behavioral tendencies, *B* = 1.17, *t*(236) = 7.09, *p* ˂ .001, *r* = .42, 95%CI [0.85, 1.50], specifically, participants in the prosocial condition (*M* = 5.73, *SD* = 1.00) rated their co-participant to be more moral than those in the antisocial condition (*M* = 4.56, *SD* = 1.74). The main effect of narcissism was also significant, *B* = 1.40, *t*(236) = 4.28, *p* ˂ .001, *r* = .27, 95%CI [0.76, 2.04]. The two-way interaction between behavioral tendencies and narcissism was also significant, *B* = -3.00, *t*(236) = -5.22, *p* ˂ .001, *r* = .32, 95%CI [-4.13, -1.87]. Results of simple slope analysis showed that participants with low narcissism rated their co-participant in the prosocial condition as more moral than the one in the antisocial condition, *B* = 2.05, *t*(236) = 8.71, *p* ˂ .001, *r* = .49, 95%CI [159, 2.51], while the difference was not significant for those with high narcissism, *B* = 0.30, *t*(236) = 1.26, *p* = .209, *r* = .08, 95%CI [-0.17, 0.76].

### Reward

When controlling for agreeableness and extraversion, the results of Model 1 (PROCESS) revealed a significant main effect of behavioral tendencies on reward, such that participants in the prosocial condition (78.51%, *N* = 95 out of 121) were 1.82 times more likely to offer a high (vs. low) reward to the co-participant than those in the antisocial condition (66.94%, *N* = 81 out of 121), *B* = 0.60, *p* = .043, 95%CI [0.02, 1.18]. The main effect of narcissism, *B* = -0.02, *p* = .971, *OR* = 0.98, 95%CI [-1.16, 1.12], and the interaction were not significant, *B* = -1.10 *p* = .284, *OR* = 0.34, 95%CI [-3.10, 0.91].

### Punishment

When controlling for agreeableness and extraversion, the BL regression revealed a significant main effect of the co-participant's behavioral tendencies, *B* = -1.04, *p* = .005, 95%CI [-1.77, -0.11], with participants in the antisocial condition (76.03%, *N* = 92 out of 121) being 2.82 times more likely to punish the co-participant than participants in the prosocial condition (61.16%, *N* = 74 out of 121). The main effect of narcissism was also significant, *B* = 4.61, *p* < .001, *OR* = 100.00, 95%CI [3.11, 6.10]. However, the interaction was not significant, *B* = 0.53, *p* = .697, *OR* = 1.70, 95%CI [-2.15, 3.22].

The NB regression model revealed that the main effects of narcissism, *B* = 0.22, *p* = .529, 95%CI [-0.46, 0.90], and behavioral tendencies, *B* = 0.03, *p* = .836, 95%CI [-0.28, 0.35], and the interaction effect, *B* = 0.20, *p* = .775, 95%CI [-1.16, 1.55], were not significant.

### Mediated Moderation Model

We investigated whether participants’ recognized anti/prosociality could explain their reward and punishment responses using Model 8 in PROCESS when controlling for participants’ agreeableness and extraversion. The first step indicated a significant interaction effect between behavioral tendencies and narcissism on recognized anti/prosociality, *B* = -4.20, *t*(236) = -9.98, *p* <.001, *r* = .54, 95%CI [-5.02, -3.37]. Specifically, compared with low narcissists, *B* = 2.90, *t*(236) = 16.83, *p* < .001, *r* = .73, 95%CI [2.56, 3.24], high narcissists displayed a smaller difference in recognized anti/prosociality between the two conditions, *B* = 0.45, *t*(236) = 2.59, *p* = .010, *r* = .16, 95%CI [0.11, 0.79].

The second step showed that recognized anti/prosociality positively predicted reward (*B* = 0.38, *p* = .018, 95%CI [0.06, 0.69]), and negatively predicted punishment (*B* = -0.60, *p* = .002, 95%CI [-0.98, -0.23]) respectively when controlling for co-participants’ behavioral tendencies, narcissism, and their interaction. The third step, examining the indirect effect of behavioral tendencies on reward and punishment through recognized anti/prosociality as a function of narcissism, revealed a significant index with reward (*B* = -1.58, 95%CI [-3.30, -0.14]), and punishment (*B* = 2.53, 95%CI [0.54, 5.45]), supporting indirect effects.

## Conclusion

When controlling for participants’ s agreeableness and extraversion, the results on moral character evaluation and reward remained similar as before in four studies. As for punishment, only the overall interaction effect between behavioral tendencies and narcissism on punishment was not significant in Study 3, but the indirect interaction effect was significant and thus was consistent with previous results.

# Additional Simple Slopes: Interpretation of Interaction Effects on Responsiveness by Looking at the Effect of Narcissism Within Each of the Conditions

Below we report additional simple slopes results and interpret the interaction effects between the actor’s behavior (or the co-participant’s behavioral tendencies) and narcissism on moral character evaluation, reward, and, punishment from the perspective by looking at the relationship between narcissism and response measurements (i.e., moral character evaluation, reward and punishment) within each of the two behavior conditions separately.

## Study 1

### Moral Character Evaluation

The anticipated two-way interaction between actor's behavior and narcissism on moral character evaluation was significant, *B* = 2.19, *t*(436) = 4.75, *p* < .001, *r* = .22, 95%CI [1.29, 3.10]. Simple slopes results showed that high narcissists (vs. low narcissists) attributed higher moral character of the target in the antisocial condition, or to put it differently, they rated the target in the antisocial condition as less immoral, *B* = 1.55, *t*(440) = 4.63, *p* < .001, *r* = .22, 95%CI [0.89, 2.21]; while high narcissists (vs. low narcissists) in the control condition perceived the actor as less moral, *B* = -0.69, *t*(440) = -2.25, *p* = .025, *r* = .11, 95%CI [-1.30, -0.09].

## Study 2

### Moral Character Evaluation

The two-way interaction between actor's behavior and narcissism on moral character evaluation was significant, *B* = -1.56, *t*(245) = -2.73, *p* = .006, *r* = .17, 95%CI [-2.69, -0.44]. Simple slopes results showed that high narcissists (vs. low narcissists) in the prosocial condition showed the trend of rating the actor as less moral (not significant), *B* = -0.41, t(245) = -0.98, *p* = .326, r = .06, 95%CI [-1.24, 0.41], and low narcissists (vs. high narcissists) in the control condition rated the actor as less moral, *B* = 1.15, t(245) = 2.95, *p* = .004, r = .19, 95%CI [0.38, 1.92].

## Study 3

### Moral Character Evaluation

The two-way interaction between the co-participant's behavioral tendencies and narcissism on moral character evaluation was significant, *B* = 3.04, t(245) = 16.33, *p* < .001, r = .72, 95%CI [2.68, 3.41]. Simple slope results showed that high narcissists (vs. low narcissists) in the prosocial condition showed the trend of rating the co-participant as less moral (not significant), *B* = -0.46, t(245) = -0.96, *p* = .336, r = .06, 95%CI [-1.40, 0.48], and low narcissists (vs. high narcissists) in the antisocial condition evaluated the actor as less moral, *B* = 2.35, t(245) = 4.98, *p* < .001, r = .30, 95%CI [1.42, 3.28].

### Reward

The two-way interaction between the co-participant's behavioral tendencies and narcissism on reward was significant, *B* = -2.12, *p* = .042, 95%CI [-4.13, -0.10]. The simple slope results showed that in the antisocial condition, high narcissists (vs. low narcissists) were more likely to give high reward to their co-participant, *B* = 2.54, *p* < .001, 95%CI [1.08, 3.99]; while in the prosocial condition, high and low narcissists did not show significant differences in reward, *B* = 0.42, *p* = .555, 95%CI [-0.97, 1.81].

### Punishment

The two-way interaction between the co-participant's behavioral tendencies and narcissism on punishment (dichotomous; BL model) was significant, *B* = 2.89, *p* = .022, 95%CI [0.41, 5.37]. The simple slope results showed that high narcissists (vs. low narcissists) were more likely to punish their co-participant in both the antisocial, *B* = 2.73, *p* < .001, 95%CI [1.31, 4.15], and the prosocial conditions, *B* = 5.62, *p* < .001, 95%CI [3.58, 7.65], while the difference between high and low narcissists in punishment was larger in the prosocial condition.

## Study 4

### Moral Character Evaluation

The two-way interaction between the co-participant's behavioral tendencies and narcissism on moral character evaluation was significant, *B* = -3.05, t(238) = -5.36, *p* < .001, r = .33, 95%CI [-4.17, -1.93]. Simple slope results revealed that high and low narcissists showed no difference in the moral character evaluation of the target in the prosocial condition, *B* = 0.08, t(238) = 0.21, *p* = .834, r = .01, 95%CI [-0.67, 0.83], while low narcissists (vs. high narcissists) evaluated the target in the antisocial condition as less moral, *B* = 3.13, t(238) = 7.42, *p* < .001, r = .43, 95%CI [2.30, 3.96].

## Conclusion

According to the simple slope results in four studies, it appears that narcissists’ hypo-responsiveness regarding moral character evaluation was mainly driven by their relatively low sensitivity to antisocial and neutral behaviors.

# Results After Removing Participants Who Failed on the Manipulation Check

## Study 1

### Moral Character Evaluation

We used Model 3 in PROCESS to test the effects of actor’s behavior, narcissism, and self-relevance on moral character evaluation after removing 32 participants who failed on the manipulation check item. The results yielded a significant main effect of actor’s behavior, *B* = -2.71, *t*(404) = -24.17, *p* ˂ .001, *r* = .77, 95%CI [-2.94, -2.49], specifically, participants in the antisocial condition (*M* = 1.95, *SD* = 1.23) rated the actor to be less moral than those in the control condition (*M* = 4.65, *SD* = 1.05). There was no significant main effect of narcissism, *B* = 0.10, *t*(404) = 0.42, *p* = .672, *r* = .02, 95%CI [-0.36, 0.55], nor of self-relevance, *B* = 0.07, *t*(404) = 0.65, *p* = .514, *r* = .03, 95%CI [-0.15, 0.29]. The three-way interaction, *B* = 0.49, *t*(404) = -0.53, *p* = .598, *r* = .03, 95%CI [-1.33, 2.30], and the two-way interactions between self-relevance and actor’s behavior, *B* = 0.23, *t*(404) = 1.01, *p* = .314, *r* = .05, 95%CI [-0.22, 0.67], and between self-relevance and narcissism, *B* = 0.26, *t*(404) = 0.56, *p* = .578, *r* = .03, 95%CI [-0.65, 1.17], were not significant. However, the two-way interaction between actor’s behavior and narcissism was significant, *B* = 1.65, *t*(404) = 3.57, *p* ˂ .001, *r* = .17, 95%CI [0.74, 2.56]. Results of simple slope analysis showed that both participants with low, *B* = -3.13, *t*(408) = -19.82, *p* ˂ .001, *r* = .70, 95%CI [-3.44, -2.82], and high narcissism, *B* = -2.28, *t*(408) = -14.50, *p* ˂ .001, *r* = .58, 95%CI [-2.59, -1.97], rated the actor in the control condition as more moral than the one in the antisocial condition, but the difference was smaller for those with high narcissism.

### Mediated Moderation Model

We also examined whether the interaction effect between actor’s behavior and narcissism on moral character evaluation could be explained by recognized antisocialityafter dropping participants failed on the manipulation check. The same procedure was adopted as in the manuscript. The first step indicated that the interaction effect between actor’s behavior and narcissism on recognized antisociality was significant, *B* = -2.15, *t*(408) = -5.79, *p* ˂ .001, *r* = .28, 95%CI [-2.88, -1.42]. Specifically, compared with low narcissists, *B* = 5.02, *t*(408) = 38.88, *p* < .001, *r* = .89, 95%CI [4.76, 5.27], high narcissists displayed a smaller difference in recognized antisociality between the two conditions, *B* = 3.96, *t*(408) = 30.83, *p* < .001, *r* = .84, 95%CI [3.71, 4.21].

The second step revealed a significant relationship between recognized antisociality and moral character evaluation when controlling for actor’s behavior, narcissism and their interaction, *B* = -0.35, *t*(408) = -6.12, *p* ˂ .001, *r* = .29, 95%CI [-0.47, -0.24]. Third, we examined the indirect effect of actor’s behavior on moral character evaluation via recognized antisocial as a function of narcissism, which was supported as the index of the mediated moderation was significant, *B* = 0.77, 95%CI [0.37, 1.25].

## Study 2

### Moral Character Evaluation

We used Model 1 in PROCESS to test the effects of actor’s behavior and narcissism on moral character evaluation after removing 39 participants who failed on the manipulation check item. The results yielded a significant main effect of actor’s behavior, *B* = 1.98, *t*(206) = 11.33, *p* ˂ .001, *r* = .62, 95%CI [1.64, 2.33]. Specifically, participants in the prosocial condition (*M* = 5.78, *SD* = 1.12) rated the actor to be more moral than those in the control condition (*M* = 3.84, *SD* = 1.38). There was no significant main effect of narcissism, *B* = -0.48, *t*(206) = -1.54, *p* = .126, *r* = .11, 95%CI [-1.09, 0.14]. The two-way interaction between actor’s behavior and narcissism was not significant, *B* = -0.10, *t*(206) = -0.16, *p* = .876, *r* = .01, 95%CI [-1.34, 1.15].

### Mediated Moderation Model

Despite that the interaction effect was not significant directly on moral character evaluation, we still examined whether the interaction effect worked indirectly on moral character evaluation via recognized prosociality. The procedure was the same as in the manuscript. The results of the first step revealed a significant interaction effect between actor’s behavior and narcissism, *B* = -3.25, t(206) = -5.94, *p* <.001, r = .38, 95%CI [-4.32, -2.17]. Specifically, compared with low narcissists, *B* = 4.50, *t*(206) = 21.31, *p* < .001, *r* = .83, 95%CI [4.08, 4.91], high narcissists displayed a smaller difference in recognized prosociality between the two conditions, *B* = 2.67, *t*(206) = 12.06, *p* < .001, *r* = .64, 95%CI [2.23, 3.10].

Testing the relationship between recognized prosociality and moral character evaluation when controlling for actor's behavior, narcissism and their interaction, showed a significant positive relationship, *B* = 0.37, *t*(206) = 4.82, *p* <.001, *r* = .32, 95%CI [0.22, 0.52]. Testing the indirect effect of actor's behavior on moral character evaluation through recognized prosociality as a function of narcissism, revealed a significant index, supporting the indirect effects, *B* = -1.20, 95%CI [-1.88, -0.60].

## Conclusion

The results remained consistent when dropping participants who failed on the manipulation check in Study 1. Although the overall moderation effect on moral character evaluation was not significant in Study 2, the indirect effect via recognized prosociality remained significant. All in all, the results remained similar as when all participants were included.

# Participants’ Difference in Perceived Similarity with Others

In the pre-registered Study 4, we measured perceived similarity for two reasons. First, we examined its potential mediating effect by examining whether narcissists perceived the target as being more or less similar depending on whether the other was described as an antisocial versus prosocial person. Second, we used perceived similarity as an index of sensitivity to external information to rule out the alternative explanation that narcissists are less sensitive to external information in general. To this end, in addition to the inclusion of the confirmatory manipulation in the design (i.e., communal condition: antisocial vs. prosocial, N = 242) for the first purpose, we also included two agentic conditions (i.e., unsuccessful vs. successful; N = 224) as an exploratory factor, by presenting the target as either unsuccessful or successful. For the second purpose, specifically, we compared narcissists’ perceived similarity with the other antisocial versus prosocial person and their perceived similarity with the other unsuccessful versus successful person.^[[1]](#footnote-1)^ We reported the results of perceived similarity in both confirmatory and exploratory conditions to present a holistic picture of narcissists’ perception of similarity in both contexts.

We measured perceived similarity in two ways. The first measure consisted of a three-item perceived similarity scale (Burton et al., 2017), which asked participants to indicate the extent to which they believed the co-participant was similar to them.^[[2]](#footnote-2)^ We also measured participants’ self-ratings on each of the big five personality dimensions as well as their ratings of their co-participant’s big five personality dimensions with the Ten-Item Personality Inventory (TIPI; Gosling et al., 2003). We then calculated the correlation between participants’ own scores and their ratings of the co-participant on each item of the TIPI to create a perceived similarity index (Gonzaga et al., 2007; Luo et al., 2008),^[[3]](#footnote-3)^ which ranged from -1 to1. Positive and negative correlations indicate that participants perceived themselves to be either similar to or dissimilar from the co-participant respectively regarding the big five personalities. The two measures of perceived similarity were strongly positively correlated with one another, *r* = .48, *p* < .001, lending support for their construct validity.

In the communal context, for the perceived similarity scale, the results showed that low narcissists perceived greater similarity with the prosocial co-participant than the antisocial co-participant, *B* = 4.01, *t*(238) = 12.47, *p* < .001, *r* = .63, 95%CI [3.38, 4.65], while high narcissists did not show a significant difference in perceived similarity when comparing the antisocial and prosocial co-participant, *B* = 0.48, *t*(238) = 1.48, *p* = .149, *r* = .10, 95%CI [-0.16, 1.11] (see Figure S3A). The results for the perceived similarity index were consistent with those using the scale, such that low narcissists perceived themselves to be more similar with the prosocial than with the antisocial co-participant, *B* = 0.66, *t*(233) = 6.95, *p* < .001, *r* = .41, 95%CI [0.47, 0.84], while high narcissists did not show a significant difference in perceived similarity when comparing the antisocial and prosocial co-participant, *B* = 0.12, *t*(233) = 1.26, *p* = .209, *r* = .08, 95%CI [-0.07, 0.31] (see Figure S3B).

In the agentic context, for the perceived similarity scale, the results showed that while low narcissists perceived less similarity with the successful than the successful co-participant, *B* = -1.30, *t*(220) = -3.74, *p* < .001, *r* = .24, 95%CI [-1.98, -0.62], high narcissists perceived significantly more similarity with the successful than with the unsuccessful co-participant, *B* = 1.30, *t*(220) = 3.77, *p* < .001, *r* = .25, 95%CI [0.62, 1.97] (see Figure S3C). The results with the perceived similarity index were consistent, showing that low narcissists perceived themselves to be more similar with the unsuccessful than the successful co-participant, *B* = -0.26, *t*(214) = -2.80, *p* = .006, *r* = .19, 95%CI [-0.44, -0.08], while again high narcissists perceived themselves to be more similar with the successful than the unsuccessful co-participant, *B* = 0.19, *t*(214) = 2.11, *p* = .036, *r* = .14, 95%CI [0.01, 0.37] (see Figure S3D).

In sum, in the communal context, perceived similarity measured in two different ways showed convergent results, suggesting that narcissists’ undifferentiated perception of similarity could just be another manifestation of their insensitivity to others’ antisocial and prosocial behaviors. Given the possibility that measured perceived similarity was an artefact of hypo-sensitivity, its capacity of capturing actual perceived similarity is limited. Therefore, we did not include perceived similarity in the mediation analyses and only focused on recognized anti/prosociality as the mediator.

In the agentic context, the results on the two types of perceived similarity were also convergent, albeit different from our findings in the communal context, suggesting that narcissists were sensitive to the success-related information since they perceived themselves to be more similar to the successful rather than the unsuccessful target, which is in line with their self-concept as successful and their tendency to self-enhance in the agentic rather than communal context (Morf & Rhodewalt, 2001). Thus, it appears that narcissists are not indiscriminately less sensitive to all contexts.

**Figure S3**

*Effects on Perceived Similarity*


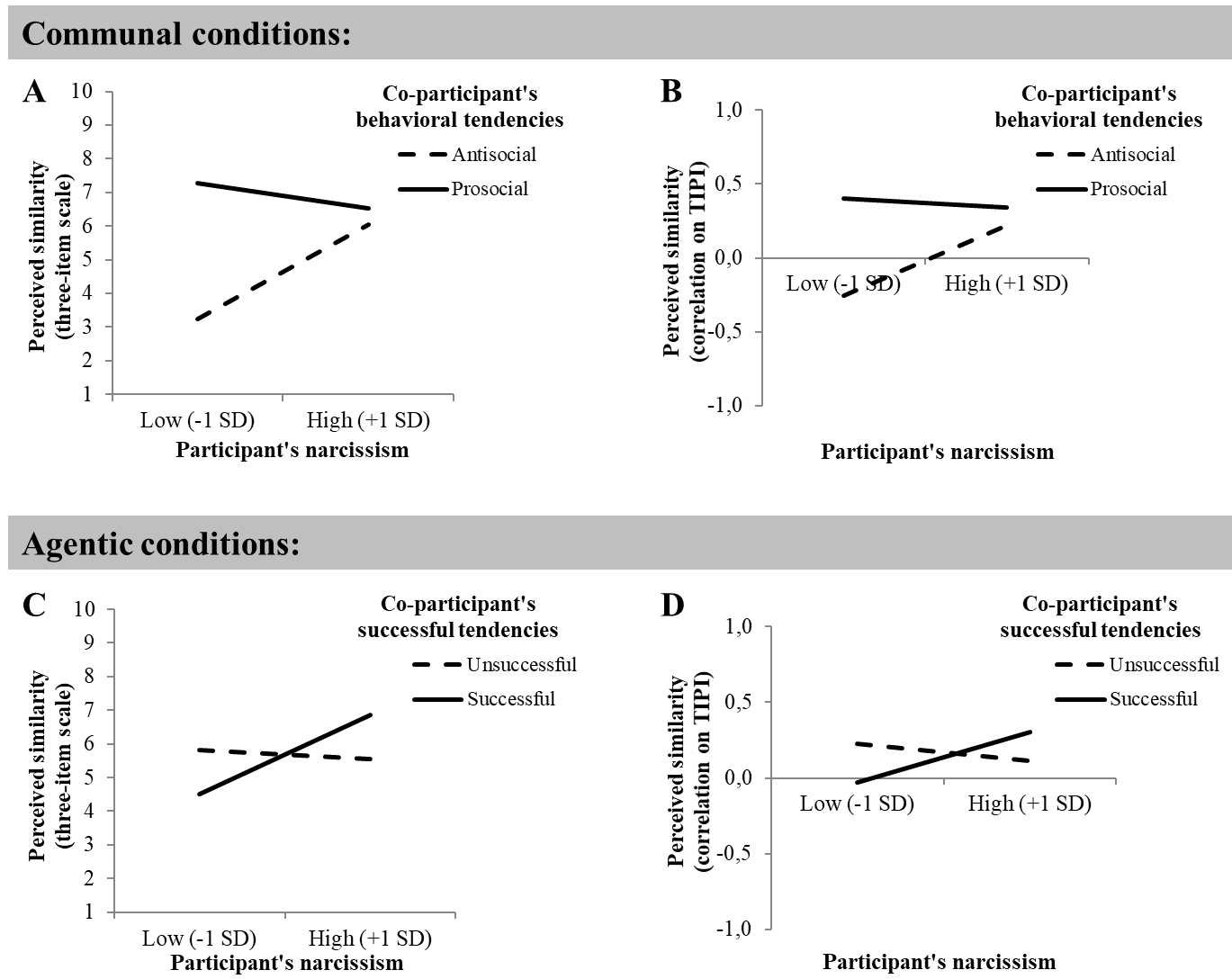


*Note*. Interaction between co-participants’ behavioral tendencies and participants’ narcissism on participants’ perceived similarity with the co-participant in the communal context (A and B); Interaction between co-participants’ successful tendencies and participants’ narcissism on participants’ perceived similarity with the co-participant in the agentic context (C and D). The perceived similarity in figures A and C was measured with the three-item perceived similarity scale (Burton et al., 2017). The perceived similarity in figures B and D was captured by the Pearson correlation representing an index of perceived similarity, calculated using the personality of the participant and the co-participant (TIPI; Golsing et al., 2003).

# Effects of Self-reported Anti/prosociality of Participants in Explaining Narcissists’ Responsiveness

In Study 4, all participants completed self-reported anti/prosociality questionnaire, which was later used to manipulate the co-participant’s behavioral tendencies (α = .64). We examined whether narcissists’ self-reported anti/prosociality could explain their hypo-responsiveness across two conditions regarding their moral character evaluation, reward, and punishment responses using Model 15 in PROCESS in both Study 3 and 4 (Figure S4).

In Study 3, the first step revealed that narcissism negatively predicted self-reported anti/prosociality, *B* = -0.75, *t*(247) = -5.95, *p* < .001, *r* = .35, 95%CI[-1.00, -0.50]. The second step examining the interaction effect between behavioral tendencies and self-reported anti/prosociality was significant on moral character evaluation, *B* = 2.14, *t*(243) = 6.75, *p* < .001, *r* = .40, 95%CI[1.51, 2.76], and reward, *B* = 1.62, *p* = .004, *OR* = 5.05, 95%CI[0.53, 2.70], but not on punishment, *B* = -1.08, *p* = .113, *OR* = 0.34, 95%CI[-2.42, 0.26] when controlling for narcissism, self-reported anti/prosociality, behavioral tendencies, and the interaction between narcissism and behavioral tendencies. Compared with participants higher on self-reported anti/prosociality, *B* = 4.84, *t*(243) = 14.89, *p* < .001, *r* = .80, 95%CI[4.21, 5.48], those lower on self-reported anti/prosociality displayed a smaller difference in moral character evaluation between the two conditions, *B* = 2.34, *t*(243) = 5.43, *p* < .001, *r* = .54, 95%CI[1.49, 3.18]. In terms of reward, participants with higher self-reported anti/prosociality were 16.85 times more likely to offer their co-participant a high (vs. low) reward in the prosocial condition than in the antisocial condition, *B* = 2.82, *p* < .001, 95%CI[1.68, 3.98], whereas those lower on self-reported anti/prosociality showed no difference in rewarding their co-participant in two conditions, *B* = 0.93, *p* = .208, *OR* = 2.53, 95%CI[-0.52, 2.38].

In the third step, the indirect effect of narcissism on three types of responses through self-reported anti/prosociality as a function of behavioral tendencies was significant for moral character evaluation (*B* = -1.60, 95%CI[-2.47, -0.88]), and for reward (*B* = -1.21, 95%CI[-2.53, -0.38]), supporting mediated moderation, but not for punishment (*B* = 0.81, 95%CI[-0.22, 2.55]). Therefore, narcissists’ lower self-reported prosociality (or higher self-reported antisociality) level was associated with their hypo-responsiveness in terms of moral character evaluation and reward but not in punishment.

The same analyses were conducted for Study 4. The first step revealed that narcissism negatively predicted self-reported anti/prosociality, *B* = -0.96, *t*(240) = -7.48, *p* < .001, *r* = .43, 95%CI[-1.21, -0.70]. The second step examining the interaction effect between behavioral tendencies and self-reported prosociality was significant on moral character evaluation, *B* = 1.48, *t*(236) = 5.52, *p* < .001, *r* = .34, 95%CI[0.95, 2.00], and reward, *B* = 1.05, *p* = .047, *OR* = 2.86, 95%CI[0.01, 2.09], but not on punishment, *B* = -0.50, *p* = .490, *OR* = 0.61, 95%CI[-1.94, 0.93] when controlling for narcissism, self-reported anti/prosociality, behavioral tendencies, and the interaction between narcissism and behavioral tendencies. Such that, compared with participants higher on self-reported anti/prosociality, *B* = 3.12, *t*(236) = 9.27, *p* < .001, *r* = .52, 95%CI[2.45, 3.77], those lower on self-reported anti/prosociality displayed a smaller difference in moral character evaluation between the two conditions, *B* = 1.21, *t*(236) = 2.65, *p* = .008, *r* = .17, 95%CI[0.31, 2.11]. As for reward, participants with higher self-reported anti/prosociality were 3.61 times (marginally) more likely to offer their co-participant a high (vs. low) reward in the prosocial condition than in the antisocial condition, *B* = 1.28, *p* = .052, 95%CI[1.30, 2.94], whereas those lower on self-reported anti/prosociality showed no difference in rewarding their co-participant in two conditions, *B* = -0.07, *p* = .940, *OR* = 0.93, 95%CI[-1.85, 1.71].

In the third step, the indirect effect of narcissism on three types of responses through self-reported anti/prosociality as a function of behavioral tendencies was significant for moral character evaluation (*B* = -1.41, 95%CI[-2.11, -0.83]), supporting mediated moderation, but not for reward (*B* = -1.00, 95%CI[-2.33, 0.02]) and punishment (*B* = 0.48, 95%CI[-1.21, 2.94]). Therefore, in Study 4, narcissists’ lower self-reported prosociality (or higher self-reported antisociality) level was associated with their hypo-responsiveness only in terms of moral character evaluation and did not generalize to reward and punishment.

**Figure S4**

*Mediated Moderation Model*


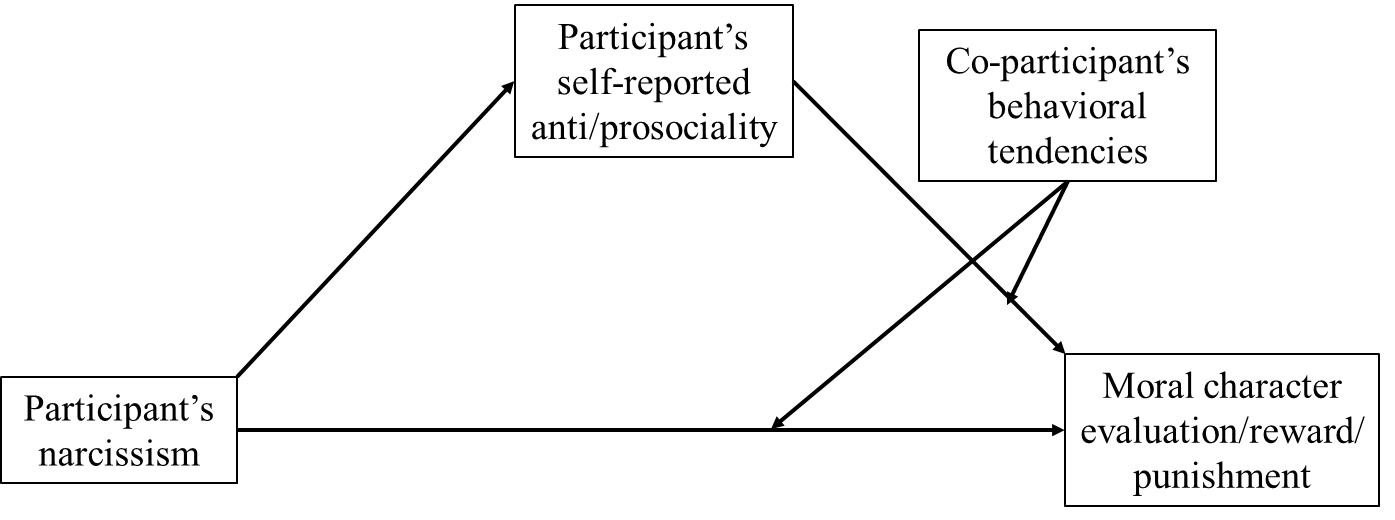


*Note.* Proposed mediated moderation model in Study 3 and 4. Co-participant’s behavioral tendencies were either antisocial or prosocial.

1. Similarly to the communal condition manipulation, participants were presented with their co-participant’s answers to a ten-item questionnaire related to success (e.g., “I am often the leader in social groups that I am a member of (e.g., sports, work, college, friends)”). The successful target scored high on five successful items and low on five unsuccessful items, whereas the unsuccessful target had the opposite scoring trend. [↑](#footnote-ref-1)
2. Three items were used to assess the degree to which participants agreed with the statements about their feelings of similarity with their co-participant (e.g., "I think my co-participant and I are similar in a lot of ways"; α = .71; Burton et al., 2017) on a 10-point scale (1 (*Strongly disagree*) to 10 (*Strongly agree*). [↑](#footnote-ref-2)
3. Note that eleven participants (five in the communal context, six in the agentic context) had no similarity value based on the correlation, because their self-ratings on personalities and/or ratings on their co-participant’s personalities measured with the TIPI were constant, which made the denominator of the Pearson correlation equation equal to zero and thus impossible to yield a correlation value. Therefore, we excluded these participants when doing the following analyses. [↑](#footnote-ref-3)
